# Supplementary material for: Feasibility of linking universal child and family healthcare and financial counselling: findings from the Australian Healthier Wealthier Families (HWF) mixed-methods study
Source: BMJ Open. 2023 Nov 22;13(11):e075651. doi: 10.1136/bmjopen-2023-075651 (PMC10668198; doi:10.1136/bmjopen-2023-075651)
Supplement: Supplementary data [file bmjopen-2023-075651supp001.pdf]

LIST OF SUPPLEMENTARY CONTENT

Supplementary Material 1: Australia's public health restrictions and economic responses during the COVID-19 pandemic.
Supplementary Figure 1. Participant flow of caregivers (CONSORT).
Supplementary Figure 2. Healthier Wealthier Families key elements, enabling ingredients, and benefits.
Supplementary Table 1: Core and subthemes identified by the implementation evaluation, with descriptions and illustrative quotes.
Supplementary Table 2: Enrolment characteristics of caregivers described by n (Sites 1-4) and n (%) (Site 5) unless specified.
Supplementary Table 3: Secondary outcome measures.
Supplementary References

Supplementary Material 1: Australia's public health restrictions and economic responses during the COVID-19 pandemic

Australia's public health restrictions during the COVID-19 pandemic 2020-21: In Australia, stay-at-home orders ('lockdown') were the main measure of disease control until late 2021 when double-vaccination levels reached 70%-80%. Victoria and NSW were the states with the highest infection rates and the longest and most severe lockdowns. Initially, all Australian states and territories experienced a national, 10-week lockdown from 23 March to 1 June 2020, which disrupted the health and social service systems and required radical change from in-person to virtual service delivery within weeks. Many healthcare workforces, including CFH, were seconded to responsibilities such as COVID-19 testing clinics and, from early 2021, vaccination efforts. Victoria experienced a second, extended, and more severe, 20-week lockdown from 8 July-23 November 2020. There were several short (less than a week) lockdowns in the intervening months, before NSW experienced its second, extended period of lockdown, which began incrementally on 26 June 2021. Victoria experienced its fifth lockdown from 16-27 July 2021, before another extended lockdown (the sixth in total) began a week later on 5 August 2021. Lockdowns in both states ended in late October 2021.

Australia's economic response to the COVID-19 pandemic: To protect against the economic fallout of lockdown (including high un- and under-employment rates), the Australian federal government rapidly implemented a suite of short-term financial supports that became available by April 2020.[1,2] The supports included an unemployment supplement ('JobSeeker') which doubled recipients' social welfare benefits from $550 to $1100 a fortnight;[1] a wage supplement for eligible businesses to retain their workforce ('JobKeeper');[2] allowing early access to superannuation;[3] and free childcare for working families.[4] Banks and creditors also allowed loan repayments to be deferred for up to 10 months. These social policy changes represented some of the largest (albeit temporary) in Australia's history. Notably, the JobKeeper and JobSeeker supplements were so significant that, by September 2020, levels of poverty and housing stress in Australia were substantially lower than the levels directly preceding COVID-19.[5] However, by September 2021, as the financial supports were reduced and withdrawn, the proportions of Australians on the lowest income support payments (which are below the poverty line) were 25% higher than before the pandemic.[5]

Price AMH, *et al. BMJ Open* 2023; 13:e075651. doi: 10.1136/bmjopen-2023-075651

BMJ Publishing Group Limited (BMJ) disclaims all liability and responsibility arising from any reliance placed on this supplemental material which has been supplied by the author(s)

Supplementary Figure 1: Participant flow of caregivers (CONSORT).

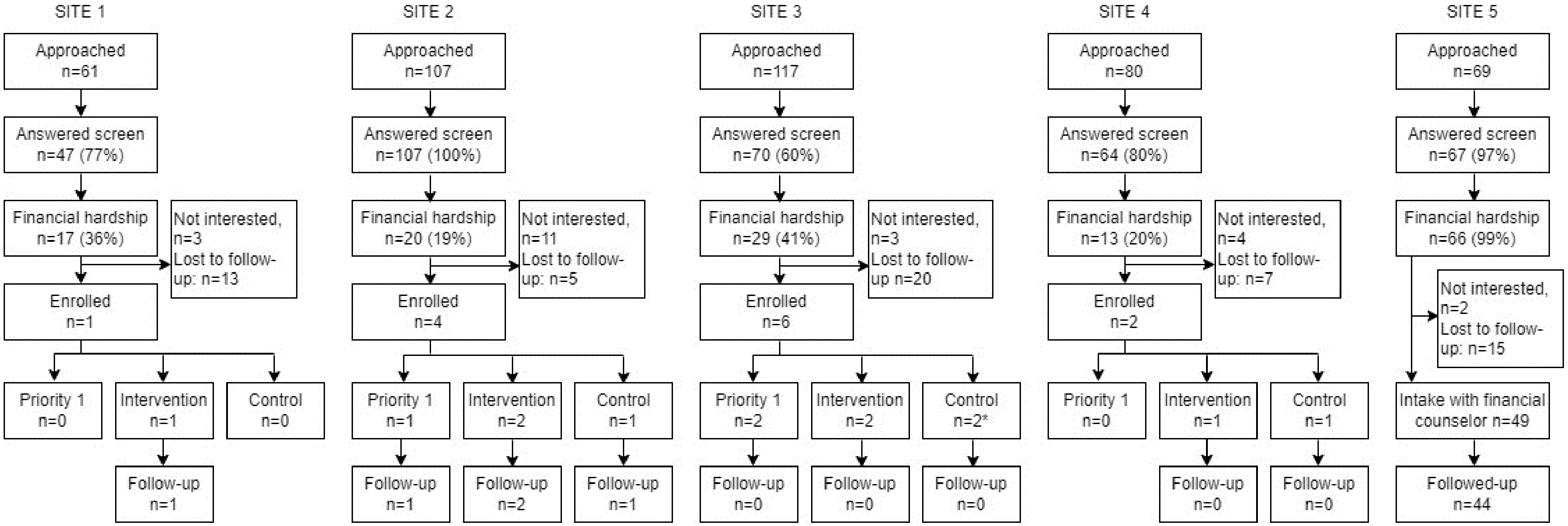

* In Site 3, the n=2 caregivers allocated to the Control arm were subsequently identified as high priority and referred to financial counselling.

Supplementary Figure 2: Healthier Wealthier Families key elements, enabling ingredients, and benefits.

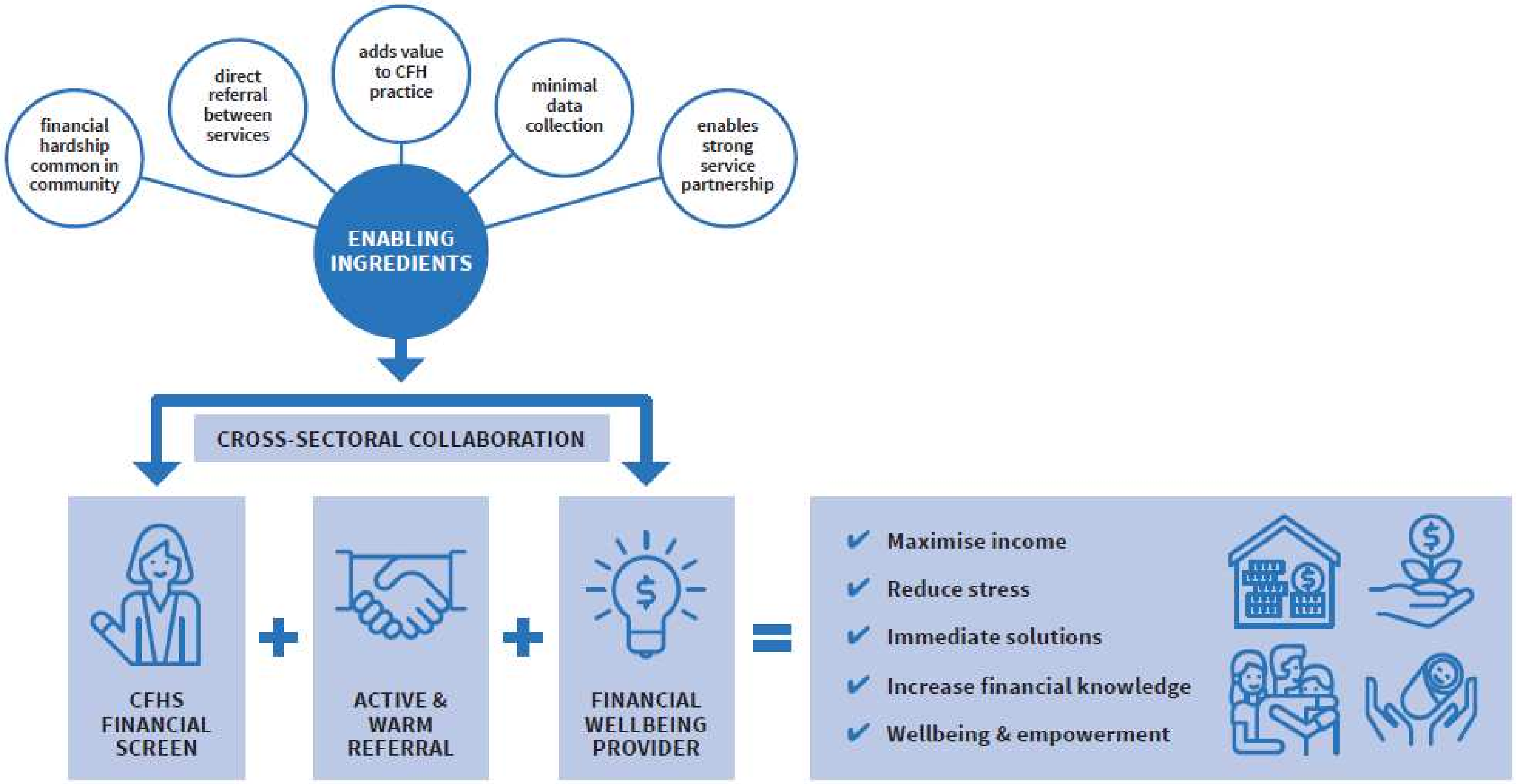

Price AMH, *et al. BMJ Open* 2023; 13:e075651. doi: 10.1136/bmjopen-2023-075651

Supplementary Table 1: Core and sub themes identified by the implementation evaluation, with descriptions and illustrative quotes.

**Core and sub themes**	**Description of subthemes with illustrative quotes**
Theme 1. Experiences and perceptions of screening and referral processes	
1.1 Child and Family Health (CFH) providers had differing opinions about the usefulness of financial screening and relevance to their own practice, which were related to delivery of universal or higher intensity services.	Some CFH providers delivering universal care did not have time to use an additional screening tool in practice.<br>*"We only get 45 minutes to see the client…then whatever research project is at the end, and it's not wanting to do it, but it's the added extra work that we have to do on top of everything else that's crammed into that little small time frame…… And obviously these projects just go out the door for us, it's just not a priority."* [*CFH PROVIDER, FOCUS GROUP PARTICIPANT, SITE 3]*<br>Some CFH providers delivering universal services thought the screening tool was out of scope of practice and made it uncomfortable for them to use and made their clients feel uncomfortable.<br>*"…it's not the question that we as child family health bring up, like how are you financially, the financial word does not pop up during our screening to be honest…I find that a little bit uncomfortable to bring up during our clinics, which is very limited because… we have the normal check and the zillion questionaries just for mum and baby." [CFH PROVIDER, FOCUS GROUP PARTICIPANT, SITE 3]*<br>Some CFH providers of universal services viewed financial screening as part of providing holistic care.<br>"*…but it is our business if that's actually causing distress for the more, you know, impacting on their ability to parent their children. So I guess that comes back to the education part again, you know, about linking, you know, the fact that financial disadvantage is at the base of a lot of concerns that families may have, although it may not be the initial identifier." [CFH PROVIDER, PARTICIPANT 21, SITE 3]*<br>CFH providers offering higher intensity services viewed the identification and response to financial hardship as usual practice.<br>*"….. financial stress for a lot of my families is the biggest component of their day-to-day psychological health. I mean, other things are barriers definitely, but when it's around the financial counselling, it's definitely one of the biggest concerns. Just how we gonna get food into my child's mouth next week……You know, some of these fines people have, are in the thousands of dollars and if they can be reduced because of support of a financial counsellor … you know, it's basic necessities … food is back into the mouths of the children, and they are able to pay another bill. And so the mental health [difficulties] decreases, and their capacity rises." [CFH PROVIDER, PARTICIPANT 11, SITE 5].*
1.2. There were issues with engagement due to the research processes.	The research processes [RCT designs] were burdensome.<br>*"I guess the motivation, again, for doing it that way was that the researcher had more information to share with the clients to then go through and see…if they did meet the criteria. But again, knowing the community,…it was difficult to engage with some people because they don't answer their telephone." [CFH PROVIDER, PARTICIPANT 21, SITE 3]*<br>Eligibility screening added complexity for clients.<br>"*the family thought they would get some incentive some money support and would say, oh, yes, I would. And you go through all that and say, oh no you're not eligible and all this and thank you very much. It is like some of them they can come back what was the point of that." [CFH PROVIDER, FOCUS GROUP PARTICIPANT, SITE 3]*
1.3 The COVID-19 pandemic affected service provision and levels of financial hardship.	Financial hardship was reduced for many families due to the government supplements in 2020.<br>*"I found particularly in my area…that a lot of people have been very cashed up, which I wouldn't normally see…Well there's been a lot of extra payments - so there was the COVID payment. And now there's a lot more services coming along who have*

**Core and sub themes**	**Description of subthemes with illustrative quotes**
	a lot of money, are actually using that money to work with families which I hadn't seen before." [CFH PROVIDER, PARTICIPANT 19, SITE 2]<br><br>There were additional pressures on CFH Providers during the COVID-19 pandemic, which exacerbated the challenges of screening.<br>*"…being short staffed and feeling under pressure. A new thing will often get forgotten and left…It was a timing thing. For us, it wasn't great…Doing phone consults during COVID-19 as well, which was new to them and took up a lot of Nurses bandwidth. Using new things then was limited." [CFH PROVIDER, PARTICIPANT 17, SITE 4]*
Theme 2. Barriers to identifying financial hardship and accepting help	
2.1 Stigma surrounding financial hardship and accepting a referral to a Financial counsellor	Service providers identified a myriad of barriers surrounding why clients may not disclose financial hardship or accept support. The most frequently identified barrier was stigma regarding disclosure of financial hardship and a fear of being judged.<br>*"It's a big shame for clients to come forward and mention that they are not able to be in charge of their money, it's a huge stigma. So they won't be very open, there will be a lot of resistance." [CFH PROVIDER, PARTICIPANT 23, SITE 3]*<br><br>Service providers noted that building trust in new services or accepting a new referral can take time with clients. Referrals may not be readily received, even though significant need is identified.<br>*"…that building up of that rapport and that gradual. Some people you need to go gently gently with and – they may not accept it then and there but further down the track they may be more receptive to it". [SERVICE PROVIDER, PARTICIPANT 15, SITE 4]*<br><br>Providers delivering higher intensity (outreach) services noted that it may be more comfortable for clients to have conversations about financial difficulties at home.<br>*"I think there's a bit of stigma. I don't know, I find it works if you actually go out and see people. Yeah, yeah, yeah, they're more comfortable, they're probably going to tell you a little bit more, as well." [CFH PROVIDER, PARTICIPANT 19, SITE 2]*
2.2 Financial counselling was not well understood by CFH providers or families.	CFH providers noted their workforce's levels of understanding; not understanding could lead some providers to de-prioritise financial screening.<br>*"I don't think the CFHN had a full understand[ing]." [CFH PROVIDER, PARTICIPANT 22, SITE 3]*<br><br>Some service providers noted that clients lacked understanding about financial counselling, and the importance of building community awareness.<br>*"… I just don't think people have enough awareness of it to realize what a difference it can make. So I think just lack of knowledge is a barrier." [CFH PROVIDER, PARTICIPANT 17, SITE 4]*<br><br>Knowledge is important for CFH providers to be able to explain financial counselling to help clients engage with services.<br>*"It takes a very very very long time to be able to capture the audience and to get the audience to understand what kind of support it is that you want to give them. And there's a lot of confusion between financial counselling and financial advising and financial planners." [CFH PROVIDER, PARTICIPANT 23, SITE 3]*<br><br>Lack of understanding of how financial counselling can provide support is a significant barrier to accepting a referral. Without knowledge, clients may not have recognised that they could benefit from the service.<br>*"Unless it's money in my pocket, it's not help to me'. Can I access money out of this, or is it just … are they just looking at ways for me to try and not spend money?" [CFH PROVIDER, PARTICIPANT 15, SITE 4]*

Core and sub themes	Description of subthemes with illustrative quotes
2.3. Clients in crisis may have other priorities.	CFH providers noted that it can be difficult for clients in crisis to engage with services; however, when people do disclose, they usually need help immediately. Identifying need before crisis point (early intervention) can be highly beneficial as it is difficult for clients to engage in complex tasks like prioritising needs when under significant stress.<br>*“And the ones that are really in crisis often are so in crisis,… they can barely know what they're going to have for dinner, let alone plan.” CFH PROVIDER, PARTICIPANT 17, SITE 4]*
2.4 Financial literacy and cultural/family considerations.	Some service providers suggested that it is important to understand the culture of financial literacy within a community to understand how to engage a family about financial hardship.<br>“*I'm mindful in this area, people are often coming from different cultural backgrounds where, you wouldn't openly talk about your finances. There may be a certain apprehension or fear … and sometimes just engaging with us initially without even going into finances.” [CFH PROVIDER, PARTICIPANT 21, SITE 3]*<br>For some families, gender roles were relevant in discussions about finances.<br>*“…for some mothers … they have said before “I don't deal with the finances - that's my husband”. So, they don't actually have any financial control.” [CFH PROVIDER, PARTICIPANT 11, SITE 5]*
Theme 3. Factors that enabled engagement with families.	It is important that service providers are supported to understand the role of financial counselling, to build trust in a referral, to be able to explain the service to families, and support families to engage with financial counselling.<br>*“So I guess it's around how we make the link for families around that if they're in a non-judgemental, non-stigmatising way, that is that if there is some issues and we could help with some extra supports around finances, if we feel that that's actually impacting upon their ability to cope with life and cope with their baby, then, and we can help them make the link that I think they are more likely to do something about it. I just don't think that they're getting the value on the what's in it for them.” [CFH PROVIDER, PARTICIPANT 20, SITE 3]*<br>Provide resources to families about financial hardship and financial counselling.<br>*“…most of our parents do like to do a bit of research on their own so anything that is in written form - whether it be an email or website or whatever - I think that would be helpful. This helps to empower them.” [CFH PROVIDER, PARTICIPANT 18, SITE 4]*<br>Ensure accessible communication that is written in plain English and inclusive to all members of the community.<br>*“The community needs educating. Making it more open and more spoken about. we've got some resources too that fairly easily accessible, that people can go to and look at. And that's what I think with the financial struggles as well … that you need more community awareness. We need more .. like your Beyond Blue websites, like your PANDA websites.” [CFH PROVIDER, PARTICIPANT 15, SITE 4]*<br>It is important that financial counselling services provide client-centred care, not service led. This was noted for methods of communication, e.g. communicating by text rather than email and doing phone appointments rather than in-person.<br>*“Doing phone appointments works for them.” [FC PROVIDER, PARTICIPANT 9, SITE 5]*

Supplementary Table 2: Enrolment characteristics of caregivers described by n (Sites 1-4) and n (%) (Site 5) unless specified.

Caregiver characteristics	RCT allocation (Sites 1-4)			Pre-post evaluation (Site 5)	
	Priority 1	Intervention	Control	Engaged	Lost
	N=3	N=6	N=4	n=44	n=5
Age, in years					
Mean [range]	40.7 [34-46]	28.6 [23-39]	29.5 [21-42]	Age not collected	
Missing	0	1	0		
Main caregiver to children					
Yes	1	5	4	41 (93.2)	5 (100.0)
No	0	0	0	3 (6.8)	0
Missing	2	1	-	-	-
Number of children					
Mean [range]	3 [3-3]	1.4 [1-2]	1.7 [1-3]	2.2 [1-7]	2.4 [1-3]
Missing	2	1	1	-	-
Partnered					
Yes	0	3	3	26 (59.1)	4 (80.0)
No	1	2	1	2 (4.6)	1 (20.0)
Missing	2	1	-	16 (36.4)	-
Household member with disability or special health care needs					
Yes	1	0	1	21 (47.7)	2 (40.0)
No	0	5	3	23 (52.3)	3 (60.0)
Missing	2	1	-	-	-
Identifies as Aboriginal and/or Torres Strait Islander					
Yes	1	0	1	0	1 (20.0)
No	0	5	3	44 (100.0)	4 (80.0)
Missing	2	1	-	-	-
Born in Australia					
Yes	1	5	2	10 (22.7)	1 (20.0)
No	0	0	2	33 (75.0)	4 (80.0)
Missing	2	1	0	1 (2.3)	-
Speaks English at home					
Yes	2	5	3	20 (45.5)	1 (20.0)
No	0	0	1	24 (54.6)	4 (80.0)
Missing	1	1	-	-	-
Education status					
Primary school (Grade 6) or less	1	1	0	10 (22.7)	2 (40.0)
High school (Grade 12) or less	0	1	4	21 (47.7)	2 (40.0)
Diploma	1	2	0	5 (11.4)	0
Bachelor	0	1	0	8 (18.2)	1 (20.0)
Missing	1	1	-	-	-
In paid work					
Yes	0	0	2	1 (2.3)	0
Parental leave	0	1	2	2 (4.6)	0
No	3	4	0	41 (93.2)	5 (100.0)
Missing	-	1	-	-	-
Looked for work in last four weeks					
Yes	0	0	0	5 (11.4)	1 (20.0)
No	3	5	4	39 (88.6)	4 (80.0)
Missing	-	1	-	-	-
Would like more work					

Caregiver characteristics	RCT allocation (Sites 1-4)				Pre-post evaluation (Site 5)	
	Priority 1	Intervention	Control		Engaged	Lost
	N=3	N=6	N=4		n=44	n=5
Yes	2	0	1		13 (29.6)	1 (20.0)
No	-	4	2		24 (54.6)	4 (80.0)
Other	0	0	0		6 (13.6)[a]	0
Missing	1	2	1		1 (2.3)	-
Household income less than $50,000 p/annum						
Yes	2	2	2		36 (81.8)	3 (60.0)
No	1	1	0		7 (15.9)	2 (40.0)
Don’t know	0	2	2		0	0
Missing	-	1	-		1 (2.3)	-

[a] Of six caregivers who responded ‘other’: n=2 were studying with the intention to gain employment and n=1 was planning to do the same; n=1 wanted to sell a failing business to return to paid employment; n=1 wanted to work but their Visa status would not allow it; and n=1 answered that they were caring for a newborn.

Supplementary Table 1: Secondary outcome measures

Site	1	2	3	4	5
**Design & reporting framework**	Pilot RCT (as per published Protocol)[6] CONSORT			Pilot RCT (simplified processes) CONSORT	Pre-post evaluation STROBE
**State (metropolitan or regional)**	Victoria (metropolitan)	Victoria (regional)	New South Wales (metropolitan)	New South Wales (regional)	Victoria (metropolitan)
**Screening period**	3 March to 3 April 2020 (1 month)	26 August 2020 to 23 November 2021 (14 months)	2 September 2020 to 6 April 2021 (4 months)	18 Oct 2021 to 16 May 2022 (7 months)	21 June 2021 to 23 May 2022 (12 months)
**CFH service tier**	Universal	Higher intensity	Universal	Universal	Higher intensity
**Randomisation unit**	Individual			Individual	Not applicable
**Secondary outcomes [quantitative measures]: *Impact***	Caregiver-reported 3- and 6-months post-enrolment:<br>1. Household income, sources of income and types of loans [questions adapted from HILDA Survey[7], Longitudinal Study of Australian Children (LSAC)[8]].<br>2. Meeting household costs from HILDA,[7] Nest Survey,[9] Community Understanding Poverty & Social Exclusion (CUPSE) [10]].<br>3. Caregiver general health [General Health Questionnaire 1 (GHQ1)[11,12] and EuroQol five-dimensional questionnaire (EQ5D)[13,14]].<br>4. Caregiver mental health [Depression, Anxiety, Stress Scale short-form (DASS-21)[15]].<br>5. Parenting efficacy [questions from LSAC[8]].<br>6. Use of financial services [study-designed questions].			Caregiver-reported at 6-months post-enrolment:<br>1. Meeting household costs (from original RCT design).[7,9,10]<br>Emotional wellbeing:<br>2. 2-item Matthey Generic Mood Questionnaire (MGMQ),[16] dichotomised into emotional distress versus not.<br>3. 8-item Personal Wellbeing Index (PWI), scored on a 10-point scale ("no satisfaction at all" to "completely satisfied"), summed to generate a total score; higher scores indicate greater wellbeing.[17]	Financial-counsellor reported at case-close or 6 months post-referral (whichever was earlier):<br>1. 2-item Matthey Generic Mood Questionnaire (MGMQ)[16].<br>2. 8-item Personal Wellbeing Index (PWI)[17].

SUPPLEMENTARY REFRENCES

1. Phillips B, Gray M, Biddle N. COVID-19 JobKeeper and JobSeeker impacts on poverty and housing stress under current and alternative economic and policy scenarios. Acton, ACT: Australian National University Centre for Social Research and Methods; 2020.
2. Cassells R, Duncan A. JobKeepers and JobSeekers: How many workers will lose and how many will gain? Bentley, WA: Curtin University; 2020.
3. Gandy S. Care expands for moms, babies. Nursing & Allied Healthweek. 1996;1(13):1.
4. Klapdor M. Parliament of Australia. COVID-19 Economic response—free child care. https://www.aph.gov.au/About_Parliament/Parliamentary_Departments/Parliamentary_Library/FlagPost/2020/April/Coronavirus_response-Free_child_care [Accessed 26 March 2021]. 2020.
5. Davidson P. A tale of two pandemics: COVID, inequality and poverty in 2020 and 2021 ACOSS/UNSW Sydney Poverty and Inequality Partnership, Build Back Fairer Series, Report No. 3, Sydney. 2022.
6. Price AMH, Zhu A, Nguyen HNJ, Contreras-Suárez D, Schreurs N, Burley J, et al. Study protocol for the Healthier Wealthier Families (HWF) pilot randomised controlled trial: testing the feasibility of delivering financial counselling to families with young children who are identified as experiencing financial hardship by community-based nurses. BMJ Open. 2021;11(5):e044488.
7. Melbourne Institute: Applied Economics & Social Research. The Household, Income and Labour Dynamics In Australia Survey: Selected Findings from Waves 1 to 17. Report. Melbourne, Australia: The University of Melbourne, Melbourne Institute; 2019.
8. Australian Institute of Family Studies. (2018). Longitudinal Study of Australian Children Data User Guide – December 2018. Melbourne: Australian Institute of Family Studies. Accessed from https://growingupinaustralia.gov.au/data-and-documentation/data-user-guide.
9. Sollis K. Measuring child deprivation and opportunity in Australia: applying the Nest Framework to develop a measure of deprivation and opporutnity for children using the Longitudinal Study of Australian Children. Canberra; 2019 February.
10. Saunders P, Zhu A. Comparing Disadvantage and Well-being in Australian Families [online]. Australian Journal of Labour Economics. 2009;12(No. 1):21-39.
11. Raat H, Botterweck AM, Landgraf JM, Hoogeveen WC, Essink-Bot M-L. Reliability and validity of the short form of the child health questionnaire for parents (CHQ-PF28) in large random school based and general population samples. J Epidemiol Community Health. 2005;59(1):75-82.
12. Waters E, Salmon L, Wake M, Hesketh K, Wright M. The Child Health Questionnaire in Australia: reliability, validity and population means. Australian and New Zealand Journal of Public Health. 2000;24(2):207-10.
13. Group TE. EuroQol-a new facility for the measurement of health-related quality of life. Health policy. 1990;16(3):199-208.
14. Viney R, Norman R, King MT, Cronin P, Street DJ, Knox S, et al. Time trade-off derived EQ-5D weights for Australia. Value in Health. 2011;14(6):928-36.
15. Henry JD, Crawford JR. The short-form version of the Depression Anxiety Stress Scales (DASS-21): construct validity and normative data in a large non-clinical sample. British Journal of Clinical Psychology. 2005;44(2):227-39.
16. Price AMH, Middleton M, Matthey S, Goldfeld S, Kemp L, Orsini F. A comparison of two measures to screen for mental health symptoms in pregnancy and early postpartum: the Matthey Generic Mood Questionnaire and the Depression, Anxiety, Stress Scales short-form. Journal of Affective Disorders. 2021;281:824-33.
17. Cummins R. International Well Being Group: Personal Wellbeing Index. Melbourne: Australian Centre on Quality of Life, Deakin University. 2004.
