# Supplementary material for: Feasibility of linking universal child and family healthcare and financial counselling: findings from the Australian Healthier Wealthier Families (HWF) mixed-methods study
Source: BMJ Open. 2023 Nov 22;13(11):e075651. doi: 10.1136/bmjopen-2023-075651 (PMC10668198; doi:10.1136/bmjopen-2023-075651)
Supplement: Author's manuscript [file bmjopen-2023-075651.draft_revisions.pdf]

BMJ Open is committed to open peer review. As part of this commitment we make the peer review history of every article we publish publicly available.

When an article is published we post the peer reviewers' comments and the authors' responses online. We also post the versions of the paper that were used during peer review. These are the versions that the peer review comments apply to.

The versions of the paper that follow are the versions that were submitted during the peer review process. They are not the versions of record or the final published versions. They should not be cited or distributed as the published version of this manuscript.

BMJ Open is an open access journal and the full, final, typeset and author-corrected version of record of the manuscript is available on our site with no access controls, subscription charges or pay-per-view fees (http://bmjopen.bmj.com).

If you have any questions on BMJ Open's open peer review process please email info.bmjopen@bmj.com

BMJ Open

The feasibility of linking universal child and family healthcare and financial counselling: findings from the Australian Healthier Wealthier Families (HWF) mixed methods study

Journal:	*BMJ Open*
Manuscript ID	bmjopen-2023-075651
Article Type:	Original research
Date Submitted by the Author:	15-May-2023
Complete List of Authors:	Price, Anna; Murdoch Children's Research Institute, Centre for Community Child Health; The University of Melbourne, Department of Paediatrics<br>White, Natalie; Murdoch Children's Research Institute, Centre for Community Child Health<br>Burley, Jade; Sydney Children's Hospitals Network Randwick; University of New South Wales, Women's and Children's Health<br>Zhu, Anna; RMIT University, School of Economics, Marketing and Finance<br>Contreras-Suarez, Diana; The University of Melbourne, Melbourne Institute: Applied Economic & Social Research<br>Wang, Si; University of New South Wales, Psychiatry and Mental Health/ School of Clinical Medicine<br>Stone, Melissa; Uniting Vic.Tas<br>Trotter, Kellie; Hume City Council, Hume Enhanced Maternal and Child Health<br>Mrad, Mona; Uniting Vic.Tas<br>Caldwell, Jane; City of Wodonga, Wodonga Enhanced Maternal and Child Health Service<br>Bishop, Rebecca; Wesley Mission<br>Chota, Sumayya; Wesley Mission<br>Bui, Lien; Child and Family Health Services<br>Sanger, Debbie; Child and Family Health Services<br>Roles, Rob; Uniting Vic.Tas<br>Watts, Amy; Murdoch Children's Research Institute, Centre for Community Child Health<br>Samir, Nora; University of New South Wales - Randwick Campus, School of Women's and Children's Health<br>Grace, Rebekah; Western Sydney University, Centre for the Transformation of early Education and Child Health; Ingham Institute, BestSTART-South West<br>Raman, Shanti; Ingham Institute, BestSTART-SWS; South Western Sydney Local Health District, Community Paediatrics<br>Kemp, Lynn; Western Sydney University, Ingham Institute; Western Sydney University, Translational Research and Social Innovation (TReSI)<br>Lingam, Raghu; University of New South Wales, School of Women and Children’s Health; Sydney Children's Hospitals Network Randwick<br>Eapen, Valsamma; Ingham Institute, BestSTART-SWS; University of New South Wales, Psychiatry and Mental Health/ School of Clinical Medicine

Woolfenden, Susan ; Local Government and Shires Association of NSW; University of New South Wales,
Goldfeld, Sharon; Murdoch Children's Research Institute, Centre for Community Child Health; The University of Melbourne, Paediatrics

Keywords: MENTAL HEALTH, Community child health < PAEDIATRICS, Primary Health Care

SCHOLARONE™
Manuscripts

I, the Submitting Author has the right to grant and does grant on behalf of all authors of the Work (as defined in the below author licence), an exclusive licence and/or a non-exclusive licence for contributions from authors who are: i) UK Crown employees; ii) where BMJ has agreed a CC-BY licence shall apply, and/or iii) in accordance with the terms applicable for US Federal Government officers or employees acting as part of their official duties; on a worldwide, perpetual, irrevocable, royalty-free basis to BMJ Publishing Group Ltd ("BMJ") its licensees and where the relevant Journal is co-owned by BMJ to the co-owners of the Journal, to publish the Work in this journal and any other BMJ products and to exploit all rights, as set out in our licence.

The Submitting Author accepts and understands that any supply made under these terms is made by BMJ to the Submitting Author unless you are acting as an employee on behalf of your employer or a postgraduate student of an affiliated institution which is paying any applicable article publishing charge ("APC") for Open Access articles. Where the Submitting Author wishes to make the Work available on an Open Access basis (and intends to pay the relevant APC), the terms of reuse of such Open Access shall be governed by a Creative Commons licence – details of these licences and which Creative Commons licence will apply to this Work are set out in our licence referred to above.

Other than as permitted in any relevant BMJ Author's Self Archiving Policies, I confirm this Work has not been accepted for publication elsewhere, is not being considered for publication elsewhere and does not duplicate material already published. I confirm all authors consent to publication of this Work and authorise the granting of this licence.

For peer review only

The feasibility of linking universal child and family healthcare and financial counselling: findings from the Australian Healthier Wealthier Families (HWF) mixed methods study

Dr Anna Price*[1,2,3], Ms Natalie White[1,2], Dr Jade Burley[4,5,6,7], Dr Anna Zhu[8], Dr Diana Contreras-Suarez[9], Dr Si Wang[10], Melissa Stone[11], Kellie Trotter[12], Mona Mrad[11], Dr Jane Caldwell[13], Rebecca Bishop[14], Sumayya Chota[15], Lien Bui[16], Debbie Sanger[17], Rob Roles[18], Dr Amy Watts[1,2], Ms Nora Samir[4,5,6], Prof Rebekah Grace[6,19,21], A/Prof Shanti Raman[6,20], Prof Lynn Kemp[6,19,21,22], Prof Raghu Lingam[4,5,6], Prof Valsamma Eapen[6,10], **Prof Susan Woolfenden[5,6,23], **Prof Sharon Goldfeld[1,2,3]

1 Centre for Community Child Health, The Royal Children's Hospital, Parkville, VIC 3052, Australia
2 Population Health, Murdoch Children's Research Institute, Parkville, VIC 3052, Australia
3 Department of Paediatrics, The University of Melbourne, Parkville, VIC 3052, Australia
4 Sydney Children's Hospital Network, Sydney, NSW 2031, Australia
5 Population Child Health Research Group, School of Women and Children's Health, University of NSW, Randwick, NSW 2031, Australia
6 BestSTART-SWS, Ingham Institute for Allied Medical Research, Liverpool, NSW 2170, Australia.
7 Centre of Excellence for The Digital Child, The University of Wollongong, Wollongong, NSW 2522, Australia
8 School of Economics, Marketing and Finance, RMIT University, Melbourne, VIC 3000, Australia
9 Melbourne Institute, Applied Economic & Social Research, University of Melbourne, Parkville, VIC 3010, Australia
10 Academic Unit of Infant, Child and Adolescent Services (AUCS), Psychiatry and Mental Health/ School of Clinical Medicine, UNSW Medicine & Health, NSW 2170, Sydney, Australia
11 Uniting Vic.Tas, Epping VIC 3076, Australia
12 City of Hume Enhanced Maternal Child Health Service, Hume VIC 3064, Australia
13 City of Wodonga Enhanced Maternal and Child Health Service, Wodonga VIC 3689, Australia
14 Wesley Mission, Sydney, NSW 2000 Australia
15 Wesley Mission, Fairfield, NSW 2165 Australia
16 Child and Family Health, Fairfield NSW 2165 Australia
17 Child and Family Health, Albury Community Health, Albury NSW 2640, Australia
18 Uniting Vic.Tas, Broadmeadows VIC 3047, Australia
19 Transforming early Education and Child Health (TeEACH) Research Centre, Western Sydney University, Westmead, NSW 2560, Australia
20 Community Paediatrics, South Western Sydney Local Health District, Liverpool, NSW 2170, Australia
21 Translational Health Research Institute, Western Sydney University, Penrith South, NSW 2750, Australia
22 Translational Research and Social Innovation (TReSI), School of Nursing and Midwifery, Western Sydney University, Penrith South, NSW 2750, Australia
23 Sydney Medical School, University of Sydney, NSW 2006, Australia

*Corresponding author email: anna.price@mcri.edu.au
**Joint senior authors

For peer review only

ABSTRACT

Objectives: "Healthier Wealthier Families" (HWF) seeks to reduce financial hardship in the early years by embedding a referral pathway between Australia's universal child and family health (CFH) services and financial counselling. This pilot study investigated the feasibility and short-term impacts of HWF, adapted from a successful Scottish initiative.

Methods: Setting: CFH services in five sites across two states, coinciding with the COVID-19 pandemic. Participants: Caregivers of children aged 0-5 years experiencing financial hardship (study-designed screen). Design: Mixed methods. With limited progress using a randomised trial (RCT) design in Sites 1-3 (March 2020-November 2021), qualitative interviews with service providers identified implementation barriers including stigma, lack of knowledge of financial counselling, low financial literacy, research burden and pandemic disruption. This informed a simplified RCT protocol (Site 4) and direct referral model (no randomisation, pre-post evaluation, Site 5) (June 2021 to May 2022). Intervention: Financial counselling; Comparator: Usual care (Sites 1-4). Feasibility measures: proportions of caregivers screened, enrolled, followed-up, and who accessed financial counselling. Impact measures: finances (quantitative) and other (qualitative) to 6 months post-enrolment.

Results: 355/434 caregivers completed the screen (60%-100% across sites). In RCT sites (1-4), 79/365 (19%-41%) reported hardship but less than one-quarter enrolled. In Site 5, n=66/69 (96%) caregivers reported hardship and 44/66 (67%) engaged with financial counselling; common issues were utility debts (73%), and obtaining entitlements (43%) or material aid/emergency relief (27%). Per family, financial counselling increased income from government entitlements by an average $6,504 annually plus $784 from concessions, grants, brokerage, and debt waivers. Caregivers described benefits (qualitative) including reduced stress, practical help, increased knowledge, and empowerment.

Conclusions: Financial hardship screening via CFH was acceptable to caregivers, direct referral was feasible, but individual randomisation was infeasible. Larger-scale implementation will require careful, staged adaptations where CFH populations and the intervention are well-matched, and low burden evaluation.

Registration: ACTRN12620000154909.

For peer review only

Strengths and limitations of this study

- The iterative approach to piloting in five sites across two Australian states, and during the COVID-19 pandemic, enabled identification of design components that contributed to feasibility.
- The study prioritised families who are often excluded from research and used a pragmatic and high quality mixed methods approach.
- Limitations included small sample sizes, short-term follow-up, lack of control group, and lack of quantitative data on non-financial impacts.
- The lessons from this pilot can inform planning for implementation and evaluation at scale, to maximise the potential benefits generated by the model.

Original protocol: http://dx.doi.org/10.1136/bmjopen-2020-044488

Funding statement: The Healthier Wealthier Families (HWF) pilot was supported by funding from the Helen Macpherson Smith Trust (Impact Grant #9523), The Corella Fund, the Murdoch Children's Research Institute (MCRI), Health@Business and University of New South Wales (UNSW) Medicine Collaboration Seed Funds Grant, the Population Child Health Group at the UNSW, Sydney Partnership for Health, Education, Research and Enterprise (SPHERE), and the Best-START SW at the Ingham Institute. The MCRI administered the philanthropic grants and provided infrastructural support (as study sponsor) to its staff but played no role in the conduct or analysis of the trial. Research at the MCRI is supported by the Victorian Government's Operational Infrastructure Support Program. Author AP was supported by The Erdi Foundation Child Health Equity (COVID-19) Scholarship; Author SW was supported by a National Health and Medical Research Council (NHMRC) Career Development Fellowship (#1158954); Author SG was supported by a NHMRC Practitioner Fellowship (#1155290).

Competing interests: None declared.

Data sharing: Data are available upon reasonable request to hwf.study@mcri.edu.au (subject to ethical and legal approvals).

Keywords: child development, poverty, mental health, parenting, community child health.

For peer review only - http://bmjopen.bmj.com/site/about/guidelines.xhtml

1. INTRODUCTION

Strengthening financial security can buffer families from early adversity and enhance the environments that help children thrive.[1] During the first 1000 days of life (conception to 2 years), the human brain develops more rapidly than at any other time, laying the foundation for ongoing health and development.[2] Financial hardship can disrupt this foundational period.[1, 2] In Australia, a high-income country with universal health, education and social services, one in six children live below the poverty line, defined as 50% of median income using the traditional government measure.[3, 4] One in three children experience material deprivation, missing out on essential items such as food and healthcare due to cost.[3] Even by the time Australian children start school, developmental inequities due to adverse social conditions are evident.[5] Children living in the least advantaged suburbs have three times the developmental vulnerability of the most (19% versus 7%).[5] Early adversity translates to increased risks of poorer socioemotional functioning, school failure, chronic disease, mental illness, reduced economic opportunity, and intergenerational adversity.[2, 6] By adopting policies and practices that address early adverse conditions, it is estimated that Australia could reduce poor developmental outcomes in middle childhood by 50%-70%.[7]

While the mechanisms are complex, it is understood that increased household income benefits children directly through better food, stable housing, and healthcare (the investment model), as well as indirectly through improving parental mental health and thus parental capacity (the family stress model).[1] Healthcare offers avenues for addressing financial hardship; yet, this remains a perennial challenge for services that are designed to treat health symptoms but not their social causes.[8-10] In high-income countries with universal welfare platforms such as Australia and the United Kingdom (UK), government assistance is available to ease financial stress. However, low take-up of benefits is common, with an estimated 40%-80% of eligible recipients missing out.[11] 1.3 million Australians were found to be missing out on benefits in 2004,[12] when this issue was last publicly addressed. Common explanations for not receiving benefits include lack of awareness, system complexity, and stigma.[13]

Across many high-income countries, financial counselling services are freely available to help people overcome barriers to accessing entitlements and other financial supports, typically funded by governments. In Australia, however, services remain embedded in not-for-profit organisations and unconnected to the broader system.[14] Our systematic review of health-financial linkage models for families with children aged 0-5 years identified only four international models, with limited randomised trial-level evidence.[15] Substantial benefits were demonstrated by a Scottish referral model ("Healthier Wealthier Children"), which engaged health workers in early years services to identify and refer eligible caregivers to money advice workers. Two pre-post evaluations conducted between 2010 and 2013 reported average annual financial gains per caregiver of £1661 (n=2516) and

For peer review only - http://bmjopen.bmj.com/site/about/guidelines.xhtml

£1919 (n=2289).[16, 17] Positive spill-over effects included improved health, housing and quality of life. By 2020, 10 years after it began, it had generated 27,000 referrals and over £36 million in financial gain, and is now a requirement of Scotland's Child Poverty action plan.[18]

The current study, "Healthier Wealthier Families" (HWF), sought to adapt the Scottish model to the Australian service context and evaluate the model via a pilot randomised controlled trial (RCT).[14] Specific aims were to test the (1) feasibility of the HWF referral model and (2) short-term impacts on household finances, caregiver health, parenting efficacy and use of financial services. The overarching goal was to inform the methods and resourcing for a definitive, multisite effectiveness trial that would test whether the HWF model could reduce financial hardship for Australian families when delivered at scale.

2. METHODS AND ANALYSIS

2.1 Ethical approval

This research was approved by the Human Research Ethics Committees of The Royal Children's Hospital (HREC/57372/RCHM-2019; Sites 1, 2, 4-5), and South West Sydney Local Health District (2019/ETH13455; Site 3).

2.2 Setting

Australia's universal Child and Family Health (CFH) services offer free, community-based healthcare to families with children from birth to school entry. Available in all Australian jurisdictions, CFH services are delivered by nurses, often in partnership with allied health providers, and working with interpreters as needed. The services are mostly clinic-based and offer broad ranging support for children's health, development, and parental wellbeing. In some jurisdictions, CFH services offer higher intensity support (more, longer, and home-based appointments) for families experiencing increased health risks and social adversity. These service streams are differentiated with the labels 'universal' and 'higher intensity' throughout.

Australian CFH services already identify and respond to adverse experiences such as poor parental mental health and family violence. While the service frameworks recognise financial hardship as a social adversity affecting child and family outcomes, there is a gap in practice in systematically identifying and responding to this need. The HWF pilot was tested with CFH services in lower socioeconomic communities in Australia's two most populous states, Victoria and New South Wales (NSW). The study was planned before the COVID-19 pandemic, but implementation and evaluation occurred during the pandemic and were deeply impacted by Australia's public health restrictions and economic responses. In brief, stay-at-home orders (lockdown) were Australia's main measure of

For peer review only - http://bmjopen.bmj.com/site/about/guidelines.xhtml

disease control until November 2021, when vaccination levels reached 70-80%. This required CFH and financial counselling services to shift to telehealth within weeks, and saw many CFH services seconded to other pandemic-related duties. To offset the economic fallout of lockdown, the government implemented a range of financial supplements and supports in April 2020. By September 2020, this financial response had reduced poverty and housing stress beyond pre-pandemic levels; however, financial hardship had rebounded a year later after financial supports were withdrawn.[4] For more information on Australia's pandemic responses, please see the detailed description in Supplementary Material 1.

2.3 Design

When the HWF pilot study was registered with the Australian and New Zealand Controlled Trials Registry (ACTRN12620000154909, 13 February 2020), it was designed as a feasibility RCT in one Victorian site. However, implementation and evaluation during the COVID-19 pandemic required protocol iterations developed through continuous quality improvement. When recruitment ended on 30 June 2022, we had attempted iterations of HWF in five sites across two states. Overall, the model was infeasible in Sites 1-4 and feasible in Site 5. Figure 1 shows the progression and design elements of the study. Recruitment began in Site 1 in March 2020, before pausing five weeks later in response to Australia's national lockdown (and eventually ended in 2021 without recommencing). In early-2020, partnerships were formed with Sites 2 and 3, reflected by updates to the trial registry (2 March 2021) and the published protocol (http://dx.doi.org/10.1136/bmjopen-2020-044488, accepted 4 May 2021).[14] Table 1 describes the RCT methods as published, following the CONSORT statement.[14]

To understand barriers to feasibility encountered in 2020, a qualitative implementation evaluation commenced in February 2021 (Figure 1), informed by the Consolidated Framework for Implementation Research (CFIR).[19] The theoretical framework was reflexive thematic analysis, and the methods are detailed in Table 2. Interviews with nine service providers in Sites 2 and 3 revealed three core themes, summarised below. They are described in detail with illustrative quotes in Supplementary Table 1. We include these results in the methods section to explain why we had to alter the design approach.

1. Experiences and perceptions of the screening and referral processes:
   1.1. CFH providers had differing opinions about the usefulness of financial screening and relevance to their own practice, which were related to delivery of universal or higher intensity services.
   1.2. There were issues with engagement due to the research processes.
   1.3. The COVID-19 pandemic affected service provision and levels of financial hardship.
2. Barriers to identifying financial hardship and accepting help included:
   2.1. Stigma surrounding financial hardship and accepting a referral to a financial counsellor.

For peer review only - http://bmjopen.bmj.com/site/about/guidelines.xhtml

   2.2. Financial counselling was not well understood by CFH providers or families.
   2.3. Clients in crisis may have other priorities.
   2.4. Financial literacy and cultural and family considerations.
3. Factors that enable engagement with families with the HWF model (classified as suggestions rather than sub-themes):
   - Support service providers to understand the role of financial counselling;
   - Provide resources to families about financial hardship and financial counselling.
   - Ensure accessible and inclusive communication.
   - Financial counselling services provide client-centred care, not service-led.

These findings informed iterations to the HWF protocol that were trialled with two new sites (Sites 4 and 5, commencing mid-late 2021, see Figure 1), described next.

2.4 Design iterations

For Site 4, the RCT protocol was simplified. Research processes such as informed consent and questionnaires were shortened, and follow-up was reduced from two caregiver surveys at 3 and 6 months to one at 6 months (see Table 1).

More substantive design changes were implemented for Site 5. Instead of an RCT, the model employed direct referral with a pre-post evaluation (see Table 1). Researcher-led processes were replaced with short, CFH-led consent and evaluation using data collected by financial counsellors rather than surveys collected by the research team. The individual randomisation was removed so there was no control group. This meant that rather than the researcher doing the referral, it was done by the CFH provider at the time of the screening survey. This made it possible for the research team to support the CFH and financial counselling providers to build trusted, working relationships with one another. The design also reflected how the referral pathway would operate in the real-world context, i.e. without a researcher intervening between screening and referral.

In January-June 2022, we conducted a second phase of the implementation evaluation to understand barriers to and enablers of feasibility in Sites 4-5 (Figure 1, methods in Table 2). This included interviews with four CFH providers in Site 4, and one CFH provider and one financial counsellor in Site 5 (no financial counsellors in Site 4 were interviewed because no caregivers engaged, see Results). The themes regarding feasibility aligned with those identified in the first phase of the implementation evaluation and summarised above, so they are synthesised together in Supplementary Table 1. We conducted interviews with eight caregivers in Site 5 who engaged with financial counselling to understand perceived impacts of the HWF model (methods described in Table 2). The results are presented as main findings in the next section. Due to the low levels of enrolment in Sites 1-4, no caregiver interviews were conducted.

For peer review only - http://bmjopen.bmj.com/site/about/guidelines.xhtml

2.5 Patient and Public Involvement

Patients and service providers were involved in the design and conduct of this research. During the feasibility stage, the choice and acceptability of financial hardship items was informed by discussions with CFH providers and financial counsellors, and between CFH providers and their clients. During the implementation evaluation, the trial processes were reviewed with providers in Sites 2-5 and caregivers in Site 5. Once the trial is published, findings (in academic and lay outputs) will be available on the study website (https://www.rch.org.au/ccch/Healthier_wealthier_families/).

3. RESULTS

3.1 Feasibility (Aim 1)

3.1.1 Completion of the financial hardship screening survey

Table 3 summarises the feasibility measures by Site. The participant flow for caregivers is presented as a CONSORT diagram in Supplementary Figure 1. Across the five sites, CFH providers approached a total of n=434 caregivers. In the four sites where a small number of CFH providers did HWF screening each week, 75%-100% of caseloads were approached. In Site 4, where all 11 nurses did screening each week, 20% of the caseload was approached. Across the five sites, a total of n=355 caregivers completed the screening survey (ranging 60% to 100% of those approached). Reasons for declining screening were collected in Sites 1 and 5 and comprised: no interest or need for financial assistance (n=7), too personal (n=1) and no reason provided (n=8).

3.1.2 Disclosure of financial hardship (eligibility)

In the universal CFH service sites, Table 3 shows that n=17/47 (36%) caregivers in Site 1, 29/70 (41%) in Site 3, and 13/64 (20%) in Site 4 disclosed financial hardship. There was greater variation between the two sites delivering higher intensity CFH services: 20/107 (19%) in Site 2 compared with 66/67 (99%) in Site 5. The low proportion in Site 2 was at least partly due to the COVID-19 pandemic income supplements in 2020-21 reducing the levels of financial hardship (see CFH provider quotes in Supplementary Table 1 and Australian data published in [4]). Across the five sites, missing data on the financial screen were minimal, with only 8 missing values across the items and none missing for the hardship summary variable.

3.1.3 Consent/enrolment and follow-up in the study

Across the four RCT sites (1-4), 79 caregivers disclosed financial hardship and were thus eligible (Table 3, Supplementary Figure 1). Of these, 58 (73%) caregivers expressed interest in the study. Three caregivers were identified as high priority for asset loss or other social risk and immediately

For peer review only - http://bmjopen.bmj.com/site/about/guidelines.xhtml

referred (see methods in Table 1); ten caregivers were allocated (6 intervention and 4 control); and 45/58 (78%) were lost to follow-up. Of 13 enrolled caregivers, 5 completed a follow-up questionnaire (all from Sites 1 and 2). In the direct referral model in Site 5, 64/67 caregivers were referred, 49/64 (77%) were contacted by the financial counsellor and 44/64 (69%) engaged with the service, which included having service provision (follow-up) data recorded and provided by the financial counsellor.

3.1.4 Caregivers who accessed, attended, and completed the intervention

In Site 3, the two caregivers who were allocated to the Control group subsequently identified as high priority and were referred to financial counselling. All 6 caregivers enrolled in Site 3 engaged with the financial counsellor, but no follow-up data were provided. Across the other RCT Sites, no other intervention or Priority 1 caregivers engaged with the financial counsellor. In Site 5, where there were 44 attending caregivers, all appointments were conducted by phone due to the COVID-19 pandemic restrictions. Two-thirds of caregivers used one appointment; 14% used two; 16% used three and 2% used four. Four caregivers missed one appointment and two caregivers missed two, and all were rescheduled. The financial counsellor spent an average of 1.9 hours (SD 1.1) in meetings with attending caregivers [range 0.5-4.75 hours], and an average of 2.2 hours (SD 2.2) outside of meetings on each caregiver's case [range 0.5-11 hours]. By 6 months post-referral, cases were fully closed for 32/44 (72%) caregivers and partially closed (mostly due to disengagement) for 12/44 (27.3%).

In Site 5, the most common issues facing caregivers were utility debts (32/44, 73%), awaiting government entitlements ('Centrelink', 19/44, 43%) and needing material aid or emergency relief (12/44, 27%). Eighteen percent of caregivers had borrowed from friends and families; other problematic debts and loans were less frequent. While no caregivers faced bankruptcy, half the caregivers disclosed Priority 1 indicators, such as imminent eviction or disconnection of utilities, or being threatened with legal action (n=17/44 (38.6%) for each category).

3.1.5 Outcome of feasibility

Overall, the feasibility measures for Sites 1-4 met definite Stop criteria for progressing to a larger scale evaluation (defined in Table 1) whereas for Site 5 they met definite Go criteria, noting that these were defined for a pilot RCT and not a pre-post evaluation.

3.2 Caregiver participant characteristics

Supplementary Table 2 presents the caregiver participant characteristics, noting that numbers in the RCT sites (1-4) are small and limit interpretation. Across the five sites, approximately 60% of caregivers who enrolled were partnered, and the number of children in their care ranged 1-7. Caregivers tended to have low levels of education and low household income (noting $50,000 per annum approximated the poverty line for a family with two adults and two children). While most enrolled caregivers were not in paid work (n=7/12 in Sites 1-4 and n=41/44 in Site 5), some expressed

For peer review only - http://bmjopen.bmj.com/site/about/guidelines.xhtml

For peer review only

a desire for more employment (n=3/9 in Sites 1-4 and n=18/44 in Site 5). The enrolled caregivers in Sites 1-4 were more likely to have been born in Australia and speak English at home. In contrast, of the n=44 caregivers in Site 5 who engaged with financial counselling, three-quarters were born overseas and just over half spoke languages other than English at home. Just under half lived with a household member with a disability or special health care needs. In Site 5, the small number of caregivers who were referred to financial counselling but lost to follow-up (n=5) appeared broadly similar to those who engaged.

3.3 Short-term impacts identified by quantitative evaluation (Aim 2)

3.3.1 Financial impacts

The lack of follow-up data from caregivers and financial counsellors in the RCT Sites 1-4 precludes the analysis described in the published Protocol [14]. For the 44 caregivers who engaged in Site 5, Table 4 shows that financial counselling established gains to household income including: (i) government benefits (Centrelink) totalling $11,007 per fortnight: an average of $355 (SD $447) for the n=31 caregivers specified; (ii) concessions totalling $12,700: an average of $508 for the 25 caregivers with amounts specified; (iii) grants totalling $7,330: an average of $564 (SD $404) for the 13 caregivers with amounts specified; (iv) brokerage totalling $1,680: an average of $129 for the 13 caregivers specified, and (v) a total of $12,800 debt waived for 3 caregivers. When these financial gains were averaged across the 44 caregivers, income from government entitlements increased $6,504 annually and families received payments totalling $784 from concessions, grants, brokerage, and debt waivers. Table 4 shows that other frequent activities included financial literacy (66%) and referrals to other agencies (41%), most commonly housing, employment, and emergency relief (see Table footnote).

3.3.2 Other (non-financial) impacts

As above, the lack of follow-up data across the RCT sites (1-4) precluded the analysis of non-financial impacts described in the published Protocol [14] and Table 1. In Site 5, the Matthey Generic Mood Questionnaire (MGMQ) and Personal Wellbeing Index (PWI) were embedded into the financial counselling intake and follow-up surveys (described in Table 1). They were subsequently added to the CFH screening from October 2021 after the first 37/69 caregivers were screened. As there were high levels of missing data on these measures, we describe them in text (not tabulated). To provide a consistent analytic sample, they are summarised for the 49 caregivers who did intake with the financial counsellor (relevant for 28 caregivers at CFH screening).

- At CFH screening:
    - 11/28 (39%) caregivers completed the MGMQ, of which 8/11 (73%) disclosed emotional distress (versus not).

For peer review only - http://bmjopen.bmj.com/site/about/guidelines.xhtml

  - Only 4/28 (8%) caregivers completed the PWI, mean 42.3 (standard deviation (SD) 4.9).
- At intake with the financial counsellor:
  - 43/49 (88%) caregivers completed the MGMQ, of which 31/43 (72%) reported high emotional distress.
  - 38/49 (78%) caregivers completed the PWI; mean 41.9 (SD 9.7).
- The follow-up survey was mostly completed by the financial counsellor based on client records and not in-person with the caregiver. As such, completion rates were low:
  - 7/49 (14%) caregivers completed the MGMQ; none reported emotional distress.
  - 11/49 (22%) caregivers completed the PWI; mean 57.2 (SD 11.2).

3.4 Short-term impacts identified by qualitative evaluation (Aim 2)

3.4.1 Caregiver characteristics (Site 5)

In Site 5, at the final appointment, the financial counsellor invited 39 caregivers to take part in the qualitative interview on behalf of the research team. Ten caregivers agreed and eight took part including one with an interpreter. Of the two who were not interviewed, one was unavailable at their scheduled time, and one was lost due to difficulties in finding an interpreter in their first language. Demographic characteristics were not collected due to ethical approval being only available for de-identified data sharing.

3.4.2 Caregiver perspectives (Site 5)

Caregivers who engaged with financial counselling were unanimous in finding the intervention beneficial. Four core themes (benefits) emerged and are presented with illustrative quotes, noting some overlap between themes.

Theme 1: Reduced stress and gratitude for emotional support and advocacy received.

Caregivers described their relationship with the financial counsellor as being empathetic and respectful. The supportive nature of this relationship helped reduce the acute sense of burden and isolation caregivers were experiencing.

> *"My financial counsellor was very supportive. She said you are not alone, there are people out there who are experiencing the same thing. So, she had a good talk to me, and after talking to her, I was able to put myself together." [CAREGIVER 4]*

Caregivers were grateful for the opportunity to share their problems with a trusted and supportive service provider who listened, and for the strong advocacy they received. They experienced unexpected relief from the sense of pressure and hopelessness they had been feeling before referral to the financial counsellor.

For peer review only - http://bmjopen.bmj.com/site/about/guidelines.xhtml

"... it's like you're sharing your feelings with someone. So I was just ... I couldn't speak to anybody about it. So, she's the one who I am open with ... open myself and explain my situation. And I got a very positive response from that time, so it was easy for me after that." [CAREGIVER 13]

Theme 2: Immediate solutions: crisis management and practical changes to organisation of finances.

Caregivers described several ways in which the financial counsellor assisted them. These included acting as a buffer between the caregiver and creditors; helping caregivers manage debt and bills, access entitlements to maximise their income and prevent a debt spiral; and supporting caregivers to prioritise bill payments according to their goals and needs.

"She was talking to me in a way that made me feel that she would take care of everything. 'Don't worry, I will have the payments divided into instalments' ... She helped me organize myself. She showed me how to organize my bills and how to make payments." [CAREGIVER 2]

This proactive and practical support provided caregivers immediate reprieve.

"Because it was a big stress, for me, because always [Utility Company] send me notice – you're late to pay, you're late to pay. I can't pay anyway, I can't do it, and I was worry. You know, they send me always notice, notice ... if they cut electricity, it's hard because winter, cold, kids. So hard ... So, I was so happy when I get [help]." [CAREGIVER 8]

Theme 3: Increased knowledge and clarity.

Caregivers had increased awareness of the support and options available and that they were entitled to access. Caregivers described the personalised advice about finances provided by the financial counsellor.

"That's why it was really useful ... she really helped with things that we have no idea existed. Can't find information on. So it was very informative." [CAREGIVER 3]

Caregivers valued the opportunity to ask questions when they did not understand something, and have their questions answered by a trusted service provider who understood their circumstances.

"What I felt when they helped me with how to make payments and how to organise myself. I told you; my parents are overseas. I thought it was like a family. My father teaching me how to make payments and how to organize myself. That's my feeling." [CAREGIVER 2]

Theme 4: Wellbeing, self-efficacy, and empowerment

Caregivers described improvements in their confidence and outlook, including more capacity and efficacy as a parent.

For peer review only - http://bmjopen.bmj.com/site/about/guidelines.xhtml

"But my social worker and financial counsellor has been able to uplift me to make that priority along with my family's needs and open my mind and eyes to see that everything has to be on time, not to burden myself to make things worse. I know my daughter needs my attention, but I know now I have to do this first in order to be able to look after her the right way." [CAREGIVER 4]

Caregivers also described having increased control of their personal situation, more stability, and feeling less stranded and stressed.

"I just can say that it is very good that if woman like me will be aware that you can raise your voice and there is help … we should have more resources to have knowledge that we can go to this way. I think it's the best way if … we have more awareness for the woman who they are like me … that there is help. No matter what situation you are in, there is help." [CAREGIVER 5]

4. DISCUSSION

This Australian pilot study aimed to test the feasibility and short-term impacts of a systematic referral pathway between universal Child and Family Health (CFH) and financial counselling services. While financial hardship screening by CFH providers was broadly acceptable to caregivers of young children, a feasible referral model was established in only one of five sites. Challenges to implementation were exacerbated by the COVID-19 pandemic. Feasibility was enabled when screening was viewed by CFH providers as adding value to practice; direct referral was embedded between services; research activities were minimal; strong working relationships between services were enabled by the research team; CFH providers conducted longer, home-based visits; and financial hardship was highly prevalent. Where the feasible model was developed, financial counselling created average gains in household income from government benefits of $125 p/week, plus additional payments totalling $784. This is substantial given that 80% of households earned less than $1,000 per week (which approximated the Australian government's poverty line for a family with two adults and two children). While we were unable to collect quantitative data on non-financial benefits, in qualitative interviews caregivers described benefits including reduced stress and gratitude, accessing immediate solutions to their financial issues, increased knowledge and clarity, and wellbeing, self-efficacy, and empowerment.

Australian policies are increasingly recognising the role of healthcare in addressing early adversities;[20] however, there is a dearth of research in Australia and internationally on the implementation and benefits of asking about early adversity in healthcare.[9, 10] In the three pilot sites delivering universal CFH services, the 20%-41% of caregivers disclosing financial hardship was comparable with Australian population level data (also collected in 2020-21) showing that one in three families could not afford essential items.[3] In the feasible HWF model (Site 5), two-thirds of

For peer review only - http://bmjopen.bmj.com/site/about/guidelines.xhtml

eligible caregivers engaged with financial counselling and the financial gains and sources (e.g. predominately government benefits) were similar to those in the Scottish evaluations, where engagement ranged 45%-54%.[16, 17] The Scottish history offers a way forward for extending the potential of the Australian model, such as engaging other early years services (e.g. antenatal or early education and care) and modifying the delivery of financial counselling through co-located or outreach services.[16]

Strengths of this study included the iterative, dynamic approach to piloting in five sites across two Australian states. Adaptation of the protocol during the COVID-19 pandemic enabled identification of design components that contributed to feasibility. Recognised by the dynamic sustainability framework,[21] this approach is integral to achieving feasible, impactful implementation – and is core to the sustainability of the Scottish program. Our study prioritised families who are often excluded from research [22] and used a pragmatic and high quality mixed methods approach. The qualitative implementation evaluation was critical for understanding the experiences of caregivers and providers, and together with the costs and resourcing used for the pilot, can inform the development and implementation of financial screening via healthcare on a larger scale in Australia and other high-income countries with comparable service systems.

Limitations include the small sample sizes and limited demographic data for describing the qualitative cohorts; the short-term follow-up; lack of control group; and lack of quantitative data on non-financial impacts. In Site 5, the high levels of missing data on emotional wellbeing measures reflected the difficulty in embedding new measures into CFH and financial counselling practice. Indeed, the latter does not typically inquire about caregivers' mental health needs because it can require a therapeutic response that is outside the scope of practice. Of the available data, there were high levels of emotional distress (low wellbeing) at screening and intake. While it was reassuring that the available measures (MGMQ and PWI) demonstrated higher emotional wellbeing at follow-up than intake, the sample sizes were too small to draw conclusions. The substantive challenges encountered in developing a feasible model of HWF (Sites 1-4), and collecting quantitative data on non-financial impacts to enable evaluation of a feasible model (Site 5) highlight the challenges that are inherent in conducting a definitive multisite effectiveness trial of HWF. Any implementation at scale will require careful and staged adaptations that test how HWF can benefit communities in the 'post-COVID-19' landscape. This includes matching of population characteristics to the healthcare intervention, and low burden evaluation through routine data collection.

The intention of HWF is to use universal healthcare to screen and respond to financial hardship, to prevent financial crisis and its negative impacts on children and families. The sizeable proportion of caregivers disclosing financial hardship in the universal CFH services was commensurate with the low socioeconomic status of the participating sites. Even so, screening for financial hardship

For peer review only - http://bmjopen.bmj.com/site/about/guidelines.xhtml

challenged many providers' perception of their model of care. The levels of financial hardship did not outweigh other negatives such as stigma, lack of knowledge of financial counselling, the burden of the research processes, or the pandemic disruption. There are parallels between this study and evaluation of the roll-out of family violence screening by CFH services in Victoria, which found that CFH providers selectively screened women about family violence.[23] Nurses avoided screening all women – typically those with lower incomes who are more likely to experience family violence – because of ongoing individual and system practice barriers experienced when identifying and responding to needs.[23] Unsurprisingly, in our pilot, implementing HWF was more feasible for CFH providers already providing higher intensity, outreach support. It was in a site where almost all families were experiencing financial hardship that the HWF model added sufficient value to providers and caregivers for use. In sites where universal CFH services are provided, a tiered care model of those screening to have psychosocial adversity being supported via an integrated child and family program (e.g. a 'one stop shop' hub) incorporating HWF model might increase acceptability and engagement with the program.

5. CONCLUSION

The challenge for the Australian health system is that interventions to address financial hardship traditionally sit "outside" the sector. This results in substantial health system inefficiencies and increased health burdens and costs.[24] Health services alone cannot redress the negative health impacts of financial hardship. The complexity of this challenge can only be meaningfully addressed by integrated, multi-sectoral, and multidisciplinary approaches. As inflation and cost of living increases, we need sustainable and cost-effective responses to financial hardship and its effects on families in the early years. There is an argument for reorienting universal services such as CFH to address the adverse social conditions facing families and driving inequitable outcomes for children and parents.[25, 26]

For peer review only

LIST OF MAIN AND SUPPLEMENTARY FIGURES AND TABLES

MAIN

Figure 1. Healthier Wealthier Families data collection phases and research designs.
Table 1: Methods for the Healthier Wealthier Families implementation and quantitative evaluation**.**
Table 2: Methods for the Healthier Wealthier Families implementation and qualitative evaluation.
Table 3. Feasibility measures by Site.
Table 4: Activities and benefits of financial counselling in Site 5.

SUPPLEMENTARY

Supplementary Material 1: Australia's public health restrictions and economic responses during the COVID-19 pandemic.
Supplementary Figure 1. Participant flow of caregivers (CONSORT).
Supplementary Table 1: Core and subthemes identified by the implementation evaluation, with descriptions and illustrative quotes.
Supplementary Table 2: Enrolment characteristics of caregivers described by n (Sites 1-4) and n (%) (Site 5) unless specified.

For peer review only

Table 1: Methods for the Healthier Wealthier Families implementation and quantitative evaluation

Site	1	2	3	4	5
**Design & reporting framework**	Pilot RCT (as per published Protocol)[14] CONSORT			Pilot RCT (simplified processes) CONSORT	Pre-post evaluation STROBE
**State (metropolitan or regional)**	Victoria (metropolitan)	Victoria (regional)	New South Wales (metropolitan)	New South Wales (regional)	Victoria (metropolitan)
**Screening period**	3 March to 3 April 2020 (1 month)	26 August 2020 to 23 November 2021 (14 months)	2 September 2020 to 6 April 2021 (4 months)	18 Oct 2021 to 16 May 2022 (7 months)	21 June 2021 to 23 May 2022 (12 months)
**CFH service tier**	Universal	Higher intensity	Universal	Universal	Higher intensity
**CFH providers screening p/week**	1-3	1	2	11	1-2
**Randomisation unit**	Individual			Individual	Not applicable
**Sample size**	180 participants (60 per site) with an anticipated 135 (75%) providing 6-month data. As Teare et al., (2014) report, this sample size is sufficient for informing the critical parameters when designing a definitive RCT (i.e., n=70/120 for continuous/binary outcomes).[27]			The target was a minimum of 60 enrolled caregivers per site (30 per arm).	The target was a minimum of 30 caregivers enrolled and referred to financial counselling.
**Participants**	Caregivers attending the participating CFH services were eligible for the study if they were the caregiver of a young child aged 0-5 years old; lived within the study's geographical and service boundaries; and reported at least one risk factor for financial hardship as screened by the CFH provider (see below). The study-designed screening items were drawn from national and local datasets describing the characteristics most likely to identify families with young children who were at risk of or experiencing poverty and deprivation,[28-30] and co-designed with CFH and financial counselling partners from Site 1 (described in the Protocol)[14]. For Sites 4-5, they were adapted following implementation evaluation and CFH provider preferences. Caregivers were excluded if they:<br>• Could not comprehend the recruitment invitation (e.g. due to substantial cognitive disability).<br>• Were already enrolled and assigned a research participant ID.<br>• Had no mechanism for contact (telephone or email).<br>• Already an active client in a financial counselling service.<br>For the RCT study designs, caregivers identified as high-risk for high impact consequences were not randomised. This group (termed "Priority 1") was referred directly to financial counselling.				
**Financial hardship criteria**	1. In the last year, because of money pressure, caregiver missed or put off: (a) Mortgage or rent repayments, (b) Electricity, gas, water bills, (c) Buying prescription medicines, (d) Paying home or car insurance.<br>2. No one in household has a paid job.<br>3. Family does not have at least $500 in savings for an emergency.			As per Sites 1-3, except Item 3 replaced with:<br>3. Other financial pressure.	As per Sites 1-3, plus the addition of:<br>4. Other financial pressure, plus description.
**Informed consent**	After being sent a welcome email with links to the secure study database, consent could be completed online by the caregiver, or by phone with the researcher.				Conducted with the CFH provider when eligibility was

For peer review only

For peer review only - http://bmjopen.bmj.com/site/about/guidelines.xhtml

Table 1: Methods for the Healthier Wealthier Families implementation and quantitative evaluation

**Site**	**1**	**2**	**3**	**4**	**5**
					established, and recorded on study database.
**Intervention arm**	The purpose of financial counselling (the intervention) is to help clients maximise their income, reduce debt and help with budgeting. The intervention follows usual service delivery, which is tailored to a caregiver's personal and financial needs. For non-English speaking clients, it is usual practice to deliver the service via a multilingual counsellor, or with an independent telephone interpreter. Following allocation to the intervention, a referral with the participant's contact information was emailed to the financial counsellor intake address, and a financial counsellor assigned. The financial counsellor contacted the participant to arrange the first appointment, which included confirming contact and demographic details; ascertaining capacity to make financial decisions; discussing and prioritising financial needs; and planning for working together. Caregivers were asked to gather and send paperwork to financial counsellors or external agencies and assisted by the financial counsellor to complete forms in a timely and accurate manner. Financial counsellors often contact and negotiate with creditors on a caregiver's behalf. Each participant had as many appointments with the financial counsellor as they needed, over an unbounded period (unrelated to the study timeline).				
**Control arm**	The comparator received usual care plus money advice that was freely available on the federally-funded Australian Securities and Investments Commission MoneySmart website.				Not applicable.
**Randomisation procedure**	A statistician prepared the randomisation schedule using block randomisation. Participants were randomly assigned to either control or intervention arm with a 1:1 allocation following a computer-generated randomisation schedule stratified by site, using permuted blocks.				Not applicable
**Blinding (masking)**	Participants were asked not to disclose their randomisation status during data collection questionnaires. The research managers and participants were aware of randomisation to enable allocation and intervention delivery. Financial counsellors were informed of intervention participants but not control participants.				Not applicable.
**Primary outcome [quantitative measures]:** ***Feasibility***	Number/proportion of:<br>• Potential participants who agreed to complete screening survey [CFH report based on service records].<br>• Eligible caregivers who consented to participate [CFH and study records].<br>• Participants who accessed, attended, and completed the intervention [financial counsellor report, caregiver report].<br>• Caregivers who completed the follow-up questionnaire [study completion records].				
**Outcome of feasibility trial for progressing to large-scale trial**	Definite Go was defined as: (i) ≥50% of eligible caregivers consenting to pilot trial; (ii) ≥60% of those in the intervention arm receiving the intervention; (iii) ≥60% retention of consented caregivers to 6-month follow-up questionnaire.<br>Definite Stop was defined as: (i) <30% of eligible caregivers consenting to pilot trial; (ii) <30% of those in the intervention arm receiving the intervention; (iii) <30% retention of consented caregivers to 6-months follow-up questionnaire.				
**Fidelity measures**	Financial counsellors were invited to complete a questionnaire for each intervention caregiver, designed to match usual data collection. It included issues/goals; frequency of caregiver contact and compliance; activities and advice; and known changes in financial status.				

For peer review only

Table 1: Methods for the Healthier Wealthier Families implementation and quantitative evaluation

Site	1	2	3	4	5
**Secondary outcomes [quantitative measures]: *Impact***	Caregiver-reported 3- and 6-months post-enrolment:<br>1. Household income, sources of income and types of loans [questions adapted from Household, Income and Labour Dynamics in Australia (HILDA)[28] Survey, Longitudinal Study of Australian Children (LSAC)[29]].<br>2. Meeting household costs [questions adapted from HILDA,[28] Australian Research Alliance for Children and Youth Nest Survey,[31] Community Understanding of Poverty and Social Exclusion (CUPSE) Survey[30]].<br>3. Caregiver general health [General Health Questionnaire 1 (GHQ1)[32, 33] and EuroQol five-dimensional questionnaire (EQ5D)[34, 35]].<br>4. Caregiver mental health [Depression, Anxiety, Stress Scale short-form (DASS-21)[36]].<br>5. Parenting efficacy [questions from LSAC[29]].<br>6. Use of financial services [study-designed questions].			Caregiver-reported at 6-months post-enrolment:<br>1. Meeting household costs (retained from original RCT design).[28, 30, 31]<br>Emotional wellbeing:<br>2. 2-item Matthey Generic Mood Questionnaire (MGMQ),[37] dichotomised into emotional distress versus not.<br>3. 8-item Personal Wellbeing Index (PWI), scored on a 10-point scale ("no satisfaction at all" to "completely satisfied"), summed to generate a total score; higher scores indicate greater wellbeing.[38]	Financial-counsellor reported at case-close or 6 months post-referral (whichever was earlier):<br>1. 2-item Matthey Generic Mood Questionnaire (MGMQ)[37].<br>2. 8-item Personal Wellbeing Index (PWI)[38].
**Data collection procedures**	Researchers contacted caregivers to invite them to complete baseline and follow-up questionnaires. Financial counsellors were invited to complete a "fidelity" questionnaire for each intervention caregiver who engaged with the service, to measure the activities and advice delivered.				Financial counsellor reported demographic, intake, and follow-up data for the pilot.
**Analysis**	Feasibility, caregiver characteristics and impact measures were described using proportions for categorical data and mean (standard deviation (SD)) for continuous data. Due to the small sample sizes, no statistical analyses (described in the Protocol)[14] were conducted.				

CONSORT: Consolidated Standards of Reporting Trials. STROBE: Strengthening The Reporting of Observational Studies in Epidemiology.

For peer review only

For peer review only - http://bmjopen.bmj.com/site/about/guidelines.xhtml

Table 2: Methods for the Healthier Wealthier Families implementation and qualitative evaluation

**Reporting framework**	Consolidated Criteria for Reporting Qualitative Research (COREQ)[39]
**Purpose**	To understand barriers and enablers to feasibility in Sites 2-5, and the impacts for caregivers in Site 5. Service changes due to the pandemic meant it was not possible to conduct the implementation evaluation in Site 1.
**Research team & reflexivity**	Four researchers (denoted (R)) conducted the implementation evaluation.
Personal characteristics	
Education and occupation	R1: Master of Public Health, project coordinator for the Healthier Wealthier Families project in Victoria.<br>R2: PhD, Postdoctoral researcher at the Centre for Community Child Health, Victoria.<br>R3: PhD, Postdoctoral fellow and project coordinator for the Healthier Wealthier Families project in NSW.<br>R4: Graduate, Research assistant for the Healthier Wealthier Families project in NSW.
Gender	All researchers identified as female.
Experience and training	R1 was trained in qualitative methods and had experience on several projects conducting qualitative interviews.<br>R2 had previously conducted and published peer reviewed qualitative research.<br>R3 had previously conducted qualitative research and was in the process of publishing their research.<br>R4 had previously conducted qualitative research working with families and children.
**Relationship with participants**	
Relationship established, participant knowledge of interviewer	• In Sites 2 & 3, R3 & R4 conducted interviews with the service providers. R3 & R4 had established relationships with the team in Site 3 from regular project meetings but no previous relationship with the provider in Site 2.<br>• In Sites 4 & 5, R2 conducted interviews with the service providers; there were no existing relationships. As R1 had previous interactions they did not conduct these interviews.<br>• In Site 5, R1 & R2 conducted the caregiver interviews; there were no existing relationships.
Interviewer characteristics	• The involvement of R1 & R3 in the conceptualisation and implementation of the project meant there was the potential for bias related to emotional investment.<br>• Reflexive journals were kept by R1 & R3 and used to document reflections after their interviews/focus group.
**Study design**	
Theoretical framework	The methodological orientation was reflexive thematic analysis.
Participant selection	
Sampling	Purposeful, convenience samples of service provider and caregiver participants were invited due to the small number of potential participants. Caregivers were remunerated with gift cards for their time in interviews (AU$40 per hour).
Method of approach	In Sites 2 & 3:<br>• The CFH management team and providers were invited to take part in the focus group and interviews by R3 via phone call.<br>• The financial counsellor was invited to take part in the interview by R4 via phone call.<br>In Sites 4 & 5:

For peer review only - http://bmjopen.bmj.com/site/about/guidelines.xhtml

Table 2: Methods for the Healthier Wealthier Families implementation and qualitative evaluation

	• Caregiver participants were asked if they would be interested in participating in an interview at their last appointment with the financial counsellor. Those that had conversational English were approached to be interviewed. Those that did not have conversational English were approached with an interpreter in their first language.<br>• In Site 4, email addresses of the service providers who participated in the project were provided by the team leader. R2 emailed to invite participation in an interview.<br>• In Site 5, all service providers were invited to participate in an interview via email by R2.<br>In all Sites, participants (caregivers and service providers) were asked to complete a consent form before interview.
Sample size	• In Sites 2 & 3: R3 conducted a focus group with 5 CFH providers and 1 interview with a CFH provider.<br>• In Site 3, R4 conducted interviews with CFH providers and 1 financial counsellor.<br>• In Sites 4 & 5: R2 conducted interviews with 6 service providers 1 caregiver.<br>• In Site 5: R1 conducted interviews with 7 caregivers.<br>All service providers and clients identified as female. No other demographic information was collected.
Non-participation	• In Site 3, 5 service providers were invited to participate and none (0) declined.<br>• In Site 4, 10 service providers were invited to participate, 4 did not respond, 1 declined, 1 agreed but was sick on the day of the interview and was not rescheduled.<br>• In Site 5, 10 caregivers were interested in participating; however, two were not interviewed. One was unavailable at their scheduled interview time. One was not engaged due to difficulties in finding an interpreter in their first language.
Setting	
Setting of data collection, and recording	• Data in Sites 2 & 3 were collected by a focus group held face-to-face and individual interviews conducted by phone. These were recorded verbatim, transcribed by an external agency, cleaned, and saved as Word document.<br>• Data in Sites 4 & 5 were collected via phone or videoconferencing. These were electronically transcribed, cleaned, and saved as Word documents.
Presence of non-participants	None.
Description of sample	Site 2 was in regional Victoria, Site 3 was in metropolitan NSW, Site 4 was in regional NSW, Site 5 was in metropolitan Victoria (see Table 1).
Site Data collection	
Interview guide	Interview guides were developed for service provider and caregiver interviews using the Consolidated Framework for Implementation Research (CFIR)[19] and included prompts from the author/researchers. They were not piloted before use.
Repeat interviews	Not conducted.
Field notes	Some field notes were made by some of the research team after interviews and the focus group.
Duration	The focus group was 50 minutes long. Interviews with service providers and caregivers were 20-50 minutes long.
Data saturation	Due to the small number of participants in this pilot study, and who were subsequently interviewed, it is unclear whether data saturation occurred. We note that the concept of data saturation is contested in the literature.[40]

For peer review only - http://bmjopen.bmj.com/site/about/guidelines.xhtml

Table 2: Methods for the Healthier Wealthier Families implementation and qualitative evaluation

Transcripts returned	Transcripts were not returned to participants for comment.
**Data analysis**	
Number of data coders	The four researchers who conducted interviews and focus group also coded the data, in collaboration with the project lead who did not conduct any interviews or the focus group.
Description of coding tree	Data were systematically categorized into themes and codes during analysis.
Derivation of themes	Themes were derived from the data. R1, R2, R3 and the project lead met several times to discuss themes. Themes were then finalised by R1, R2 and the project lead.
Software	NVivo,[41] and Microsoft Word were used for analysis of data.
Participant checking	No.

For peer review only

Table 3. Feasibility measures by Site

**Caregiver screening, eligibility and follow-up, n (%)**	**Pilot randomised controlled trial (RCT)**				**Pre-post evaluation**
	**Original (as per Protocol)[14]**			**Simplified**	
	**Site 1**	**Site 2**	**Site 3**	**Site 4**	**Site 5**
Site characteristics	Metro Vic Universal	Regional Vic Higher intensity	Metro NSW Universal	Regional NSW Universal	Metro Vic Higher intensity
Potential sample size	n/a[a]	107	117	411	69
Number of caregivers approached by CFH providers	61 (74.6)[a]	107 (100)	117 (100)	80 (19.5)	69 (100)
Of caregivers approached, n (%) who agreed to screening	47 (77.1)	107 (100)	70 (59.8)	64 (80.0)	67 (97.1)
Of caregivers approached, n (%) requested/preferred interpreter	0	0	10 (8.6)	0	15 (21.7)
Of caregivers screened, n (%) eligible (financial hardship)	17 (36.2)	20 (18.7)	29 (41.4)	13 (20.3)	66 (98.5)
Of eligible caregivers, n (%) interested in enrolling	14 (82.4)	9 (45.0)	26 (89.7)	9 (69.2)	64 (97.0)
Of eligible caregivers, n (%) who enrolled	1 (5.9)	4 (20.0)[b]	6 (20.7)[b]	2 (15.4)	49 (77)[c]
Of enrolled caregivers, n (%) who provided follow-up data	1	4	0	0	44 (90)[c]
Breakdown of financial hardship items, n (%) who agreed to screening<br>1. Missed or put off:					
(a) Mortgage or rent repayments	6 (12.8)	0	15 (21.4)	2 (3.1)	26 (39.4)
(b) Electricity, gas, water bills	13 (27.7)	8 (7.5)	15 (21.4)	9 (14.1)	46 (68.7)
(c) Buying prescription medicines	5 (11.4)	5 (4.7)	12 (17.1)	1 (1.6)	20 (29.9)
(d) Paying home or car insurance	9 (20.0)	2 (1.9)	11 (15.7)	2 (3.1)	19 (28.4)
2. No one in household has a paid job	0	20 (18.7)	18 (25.7)	5 (7.8)	50 (74.6)
3. Does not have $500 in savings	8 (17.0)	10 (9.4)	14 (20.0)	Not collected	63 (95.5)
4. Other financial pressure	Not collected	Not collected	Not collected	4 (6.3)[d]	43 (81.1)[d]

CFH: Child and Family Health. Metro: metropolitan. NSW: New South Wales. Vic: Victoria.

[a] Data on potential caregivers provided for 3 of the 5 weeks of screening (44 screened of 59 approached).

[b] Enrolments included one caregiver in Site 2 and two caregivers in Site 3 who were immediately referred for priority indicators (not randomly allocated, see Methods in Table 1).

[c] Reported by the financial counsellor instead of caregivers.

[d] Asking about other financial pressures commenced in mid-July 2021, after 16 caregivers had been screened in Site 4 (i.e. denominator is n=64) and 14 caregivers had been screened in Site 5 (denominator is n=53). Other financial pressures included: affording essential items (predominately food, n=16); general financial hardship (e.g. low income, business strain, n=13); fines and debt (e.g. car fines, childcare and Centrelink debt, n=12); unpaid bills (e.g. medical, utilities, n=3); and not specified (n=3).

For peer review only - http://bmjopen.bmj.com/site/about/guidelines.xhtml

Table 4: Activities and benefits of financial counselling in Site 5

Actions and benefits recorded in routine data	No	Not applicable	Yes	If Yes, amount range where specified
Completion of an income & expenditure statement	29 (65.9)	12 (27.3)	3 (6.8)	p/fortnight: deficits of $103, $200, $240
Check caregiver is receiving full entitlements	6 (13.6)	0	38 (86.4)	p/fortnight: $0 to $1400
Accessed all concessions that caregiver is eligible for	3 (6.8)	13 (29.6)	28 (63.6)	$0 to $2200
Is caregiver aware of Centrepay	8 (18.2)	18 (40.9)	18 (40.9)	(n/a)
Negotiated moratorium	5 (11.4)	37 (84.1)	2 (4.6)	2 credit cards totalling $16,000, one unspecified
Established/negotiated payment plan/s with creditor/s	10 (22.7)	30 (68.2)	4 (9.1)	p/fortnight: $10, $180, $200
Negotiated debt reduction or waiver/s	3 (6.8)	38 (86.4)	3 (6.8)	$300, $500, $12000
Considered or accessed grant/s	14 (31.8)	15 (34.1)	15 (34.1)	$0 to $1330
Considered or accessed no interest loan	24 (54.6)	10 (22.7)	10 (22.7)	$0 to $5000
Accessed brokerage from with agency	26 (60.5)	4 (9.3)	13 (30.2)	$100 to $265
Considered or accessed superannuation	17 (38.6)	25 (56.8)	2 (5.6)	$0, $3000
Organised external referrals [a]	21 (47.7)	5 (11.4)	18 (40.9)	(n/a)
Conducted financial literacy activities [b]	10 (22.7)	5 (11.4)	29 (65.9)	(n/a)
**Achieved**				
Avoidance of bankruptcy	0	44 (100)	0	
Avoidance or curtailment of legal action	0	39 (88.6)	5 (11.4)	
Stabilised caregiver's housing situation	6 (14.0)	31 (72.1)	6 (14.0)	
Avoided loss of utilities	0	21 (47.7)	23 (52.3)	
Avoided loss of mobile phone and internet access	0	42 (97.7)	1 (2.3)	
Anything else the caregiver wanted to change [c]	10 (22.7)	1 (2.3)	33 (75.0)	

Centrelink is a Government service which provides support to Australians who face financial hardship. Centrepay is a free bill paying service that makes regular deductions from Centrelink payments. Fortnight: biweekly (14 days). (n/a): not applicable.

[a] Top 3: housing/rent assistance (n=7); employment assistance (n=6); emergency relief services (n=3).

[b] Top 3: financial wellbeing information (n=19, noting n=8 in language other than English); government MoneySmart website (n=18); budgeting with template (n=7).

[c] Top 3: Centrelink entitlements (n=11); material aid and emergency relief (both n=7).

For peer review only

Supplementary Material 1: Australia's public health restrictions and economic responses during the COVID-19 pandemic

Australia's public health restrictions during the COVID-19 pandemic 2020-21: In Australia, stay-at-home orders ('lockdown') were the main measure of disease control until late 2021 when double-vaccination levels reached 70%-80%. Victoria and NSW were the states with the highest infection rates and the longest and most severe lockdowns. Initially, all Australian states and territories experienced a national, 10-week lockdown from 23 March to 1 June 2020, which disrupted the health and social service systems and required radical change from in-person to virtual service delivery within weeks. Many healthcare workforces, including CFH, were seconded to responsibilities such as COVID-19 testing clinics and, from early 2021, vaccination efforts. Victoria experienced a second, extended, and more severe, 20-week lockdown from 8 July-23 November 2020. There were several short (less than a week) lockdowns in the intervening months, before NSW experienced its second, extended period of lockdown, which began incrementally on 26 June 2021. Victoria experienced its fifth lockdown from 16-27 July 2021, before another extended lockdown (the sixth in total) began a week later on 5 August 2021. Lockdowns in both states ended in late October 2021.

Australia's economic response to the COVID-19 pandemic: To protect against the economic fallout of lockdown (including high un- and under-employment rates), the Australian federal government rapidly implemented a suite of short-term financial supports that became available by April 2020.[42, 43] The supports included an unemployment supplement ('JobSeeker') which doubled recipients' social welfare benefits from $550 to $1100 a fortnight;[42] a wage supplement for eligible businesses to retain their workforce ('JobKeeper');[43] allowing early access to superannuation;[44] and free childcare for working families.[45] Banks and creditors also allowed loan repayments to be deferred for up to 10 months. These social policy changes represented some of the largest (albeit temporary) in Australia's history. Notably, the JobKeeper and JobSeeker supplements were so significant that, by September 2020, levels of poverty and housing stress in Australia were substantially lower than the levels directly preceding COVID-19.[46] However, by September 2021, as the financial supports were reduced and withdrawn, the proportions of Australians on the lowest income support payments (which are below the poverty line) were 25% higher than before the pandemic.[46]

For peer review only

Supplementary Table 1: Core and sub themes identified by the implementation evaluation, with descriptions and illustrative quotes.

Core and sub themes	Description of subthemes with illustrative quotes
Theme 1. Experiences and perceptions of screening and referral processes	
1.1 Child and Family Health (CFH) providers had differing opinions about the usefulness of financial screening and relevance to their own practice, which were related to delivery of universal or higher intensity services.	Some CFH providers delivering universal care did not have time to use an additional screening tool in practice.<br>*"We only get 45 minutes to see the client…then whatever research project is at the end, and it's not wanting to do it, but it's the added extra work that we have to do on top of everything else that's crammed into that little small time frame…… And obviously these projects just go out the door for us, it's just not a priority." [CFH PROVIDER, FOCUS GROUP PARTICIPANT, SITE 3]*<br>Some CFH providers delivering universal services thought the screening tool was out of scope of practice and made it uncomfortable for them to use and made their clients feel uncomfortable.<br>*"…it's not the question that we as child family health bring up, like how are you financially, the financial word does not pop up during our screening to be honest…I find that a little bit uncomfortable to bring up during our clinics, which is very limited because… we have the normal check and the zillion questionaries just for mum and baby." [CFH PROVIDER, FOCUS GROUP PARTICIPANT, SITE 3]*<br>Some CFH providers of universal services viewed financial screening as part of providing holistic care.<br>*"…but it is our business if that's actually causing distress for the more, you know, impacting on their ability to parent their children. So I guess that comes back to the education part again, you know, about linking, you know, the fact that financial disadvantage is at the base of a lot of concerns that families may have, although it may not be the initial identifier." [CFH PROVIDER, PARTICIPANT 21, SITE 3]*<br>CFH providers offering higher intensity services viewed the identification and response to financial hardship as usual practice.<br>*"….. financial stress for a lot of my families is the biggest component of their day-to-day psychological health. I mean, other things are barriers definitely, but when it's around the financial counselling, it's definitely one of the biggest concerns. Just how we gonna get food into my child's mouth next week……You know, some of these fines people have, are in the thousands of dollars and if they can be reduced because of support of a financial counsellor … you know, it's basic necessities … food is back into the mouths of the children, and they are able to pay another bill. And so the mental health [difficulties] decreases, and their capacity rises." [CFH PROVIDER, PARTICIPANT 11, SITE 5].*
1.2. There were issues with engagement due to the research processes.	The research processes [RCT designs] were burdensome.<br>*"I guess the motivation, again, for doing it that way was that the researcher had more information to share with the clients to then go through and see…if they did meet the criteria. But again, knowing the community,…it was difficult to engage with some people because they don't answer their telephone." [CFH PROVIDER, PARTICIPANT 21, SITE 3]*<br>Eligibility screening added complexity for clients.<br>*"the family thought they would get some incentive some money support and would say, oh, yes, I would. And you go through all that and say, oh no you're not eligible and all this and thank you very much. It is like some of them they can come back what was the point of that." [CFH PROVIDER, FOCUS GROUP PARTICIPANT, SITE 3]*
1.3 The COVID-19 pandemic affected service	Financial hardship was reduced for many families due to the government supplements in 2020.<br>*"I found particularly in my area…that a lot of people have been very cashed up, which I wouldn't normally see…Well there's been a lot of extra payments - so there was the COVID payment. And now there's a lot more services coming along who have*

For peer review only - http://bmjopen.bmj.com/site/about/guidelines.xhtml

Core and sub themes	Description of subthemes with illustrative quotes
provision and levels of financial hardship.	*a lot of money, are actually using that money to work with families which I hadn't seen before." [CFH PROVIDER, PARTICIPANT 19, SITE 2]*<br>There were additional pressures on CFH Providers during the COVID-19 pandemic, which exacerbated the challenges of screening.<br>*"…being short staffed and feeling under pressure. A new thing will often get forgotten and left…It was a timing thing. For us, it wasn't great…Doing phone consults during COVID-19 as well, which was new to them and took up a lot of Nurses bandwidth. Using new things then was limited." [CFH PROVIDER, PARTICIPANT 17, SITE 4]*
Theme 2. Barriers to identifying financial hardship and accepting help	
2.1 Stigma surrounding financial hardship and accepting a referral to a Financial counsellor	Service providers identified a myriad of barriers surrounding why clients may not disclose financial hardship or accept support. The most frequently identified barrier was stigma regarding disclosure of financial hardship and a fear of being judged.<br>*"It's a big shame for clients to come forward and mention that they are not able to be in charge of their money, it's a huge stigma. So they won't be very open, there will be a lot of resistance." [CFH PROVIDER, PARTICIPANT 23, SITE 3]*<br>Service providers noted that building trust in new services or accepting a new referral can take time with clients. Referrals may not be readily received, even though significant need is identified.<br>*"…that building up of that rapport and that gradual. Some people you need to go gently gently with and – they may not accept it then and there but further down the track they may be more receptive to it". [SERVICE PROVIDER, PARTICIPANT 15, SITE 4]*<br>Providers delivering higher intensity (outreach) services noted that it may be more comfortable for clients to have conversations about financial difficulties at home.<br>*"I think there's a bit of stigma. I don't know, I find it works if you actually go out and see people. Yeah, yeah, yeah, they're more comfortable, they're probably going to tell you a little bit more, as well." [CFH PROVIDER, PARTICIPANT 19, SITE 2]*
2.2 Financial counselling was not well understood by CFH providers or families.	CFH providers noted their workforce's levels of understanding; not understanding could lead some providers to de-prioritise financial screening.<br>*"I don't think the CFHN had a full understand[ing]." [CFH PROVIDER, PARTICIPANT 22, SITE 3]*<br>Some service providers noted that clients lacked understanding about financial counselling, and the importance of building community awareness.<br>*"… I just don't think people have enough awareness of it to realize what a difference it can make. So I think just lack of knowledge is a barrier." [CFH PROVIDER, PARTICIPANT 17, SITE 4]*<br>Knowledge is important for CFH providers to be able to explain financial counselling to help clients engage with services.<br>*"It takes a very very very long time to be able to capture the audience and to get the audience to understand what kind of support it is that you want to give them. And there's a lot of confusion between financial counselling and financial advising and financial planners." [CFH PROVIDER, PARTICIPANT 23, SITE 3]*<br>Lack of understanding of how financial counselling can provide support is a significant barrier to accepting a referral. Without knowledge, clients may not have recognised that they could benefit from the service.

For peer review only - http://bmjopen.bmj.com/site/about/guidelines.xhtml

Core and sub themes	Description of subthemes with illustrative quotes
	"Unless it's money in my pocket, it's not help to me'. Can I access money out of this, or is it just … are they just looking at ways for me to try and not spend money?" [CFH PROVIDER, PARTICIPANT 15, SITE 4]
2.3. Clients in crisis may have other priorities.	CFH providers noted that it can be difficult for clients in crisis to engage with services; however, when people do disclose, they usually need help immediately. Identifying need before crisis point (early intervention) can be highly beneficial as it is difficult for clients to engage in complex tasks like prioritising needs when under significant stress.<br>*"And the ones that are really in crisis often are so in crisis,… they can barely know what they're going to have for dinner, let alone plan." CFH PROVIDER, PARTICIPANT 17, SITE 4]*
2.4 Financial literacy and cultural/family considerations.	Some service providers suggested that it is important to understand the culture of financial literacy within a community to understand how to engage a family about financial hardship.<br>*"I'm mindful in this area, people are often coming from different cultural backgrounds where, you wouldn't openly talk about your finances. There may be a certain apprehension or fear … and sometimes just engaging with us initially without even going into finances." [CFH PROVIDER, PARTICIPANT 21, SITE 3]*<br>For some families, gender roles were relevant in discussions about finances.<br>*"…for some mothers … they have said before "I don't deal with the finances - that's my husband". So, they don't actually have any financial control." [CFH PROVIDER, PARTICIPANT 11, SITE 5]*
Theme 3. Factors that enabled engagement with families.	It is important that service providers are supported to understand the role of financial counselling, to build trust in a referral, to be able to explain the service to families, and support families to engage with financial counselling.<br>*"So I guess it's around how we make the link for families around that if they're in a non-judgemental, non-stigmatising way, that is that if there is some issues and we could help with some extra supports around finances, if we feel that that's actually impacting upon their ability to cope with life and cope with their baby, then, and we can help them make the link that I think they are more likely to do something about it. I just don't think that they're getting the value on the what's in it for them." [CFH PROVIDER, PARTICIPANT 20, SITE 3]*<br>Provide resources to families about financial hardship and financial counselling.<br>*"…most of our parents do like to do a bit of research on their own so anything that is in written form - whether it be an email or website or whatever - I think that would be helpful. This helps to empower them." [CFH PROVIDER, PARTICIPANT 18, SITE 4]*<br>Ensure accessible communication that is written in plain English and inclusive to all members of the community.<br>*"The community needs educating. Making it more open and more spoken about. we've got some resources too that fairly easily accessible, that people can go to and look at. And that's what I think with the financial struggles as well … that you need more community awareness. We need more .. like your Beyond Blue websites, like your PANDA websites." [CFH PROVIDER, PARTICIPANT 15, SITE 4]*<br>It is important that financial counselling services provide client-centred care, not service led. This was noted for methods of communication, e.g. communicating by text rather than email and doing phone appointments rather than in-person.<br>*"Doing phone appointments works for them." [FC PROVIDER, PARTICIPANT 9, SITE 5]*

For peer review only

Supplementary Table 2: Enrolment characteristics of caregivers described by n (Sites 1-4) and n (%) (Site 5) unless specified.

Caregiver characteristics	RCT allocation (Sites 1-4)			Pre-post evaluation (Site 5)	
	Priority 1	Intervention	Control	Engaged	Lost
	N=3	N=6	N=4	n=44	n=5
Age, in years					
Mean [range]	40.7 [34-46]	28.6 [23-39]	29.5 [21-42]	Age not collected	
Missing	0	1	0		
Main caregiver to children					
Yes	1	5	4	41 (93.2)	5 (100.0)
No	0	0	0	3 (6.8)	0
Missing	2	1	-	-	-
Number of children					
Mean [range]	3 [3-3]	1.4 [1-2]	1.7 [1-3]	2.2 [1-7]	2.4 [1-3]
Missing	2	1	1	-	-
Partnered					
Yes	0	3	3	26 (59.1)	4 (80.0)
No	1	2	1	2 (4.6)	1 (20.0)
Missing	2	1	-	16 (36.4)	-
Household member with disability or special health care needs					
Yes	1	0	1	21 (47.7)	2 (40.0)
No	0	5	3	23 (52.3)	3 (60.0)
Missing	2	1	-	-	-
Identifies as Aboriginal and/or Torres Strait Islander					
Yes	1	0	1	0	1 (20.0)
No	0	5	3	44 (100.0)	4 (80.0)
Missing	2	1	-	-	-
Born in Australia					
Yes	1	5	2	10 (22.7)	1 (20.0)
No	0	0	2	33 (75.0)	4 (80.0)
Missing	2	1	0	1 (2.3)	-
Speaks English at home					
Yes	2	5	3	20 (45.5)	1 (20.0)
No	0	0	1	24 (54.6)	4 (80.0)
Missing	1	1	-	-	-
Education status					
Primary school (Grade 6) or less	1	1	0	10 (22.7)	2 (40.0)
High school (Grade 12) or less	0	1	4	21 (47.7)	2 (40.0)
Diploma	1	2	0	5 (11.4)	0
Bachelor	0	1	0	8 (18.2)	1 (20.0)
Missing	1	1	-	-	-
In paid work					
Yes	0	0	2	1 (2.3)	0
Parental leave	0	1	2	2 (4.6)	0
No	3	4	0	41 (93.2)	5 (100.0)
Missing	-	1	-	-	-
Looked for work in last four weeks					
Yes	0	0	0	5 (11.4)	1 (20.0)
No	3	5	4	39 (88.6)	4 (80.0)
Missing	-	1	-	-	-
Would like more work					
Yes	2	0	1	13 (29.6)	1 (20.0)
No	-	4	2	24 (54.6)	4 (80.0)

For peer review only

Caregiver characteristics	RCT allocation (Sites 1-4)			Pre-post evaluation (Site 5)	
	Priority 1	Intervention	Control	Engaged	Lost
	N=3	N=6	N=4	n=44	n=5
Other	0	0	0	6 (13.6)[a]	0
Missing	1	2	1	1 (2.3)	-
Household income less than $50,000 p/annum					
Yes	2	2	2	36 (81.8)	3 (60.0)
No	1	1	0	7 (15.9)	2 (40.0)
Don't know	0	2	2	0	0
Missing	-	1	-	1 (2.3)	-

[a] Of six caregivers who responded 'other': n=2 were studying with the intention to gain employment and n=1 was planning to do the same; n=1 wanted to sell a failing business to return to paid employment; n=1 wanted to work but their Visa status would not allow it; and n=1 answered that they were caring for a newborn.

For peer review only

REFERENCES

1. Cooper K, Stewart K. Does Household Income Affect children's Outcomes? A Systematic Review of the Evidence. Child Indicators Research. 2020;14:981-1005.
2. Shonkoff JP, Garner AS, Committee on Psychosocial Aspects of Child and Family Health, Committee on Early Childhood, Adoption, and Dependent Care, and Section on Developmental and Behavioral Pediatrics, Siegel BS, Dobbins MI, Earls MF, Garner AS, McGuinn L, Pascoe J, Wood DL. The lifelong effects of early childhood adversity and toxic stress. Pediatrics. 2012 Jan;129(1):e232-46.
3. Price AMH, Measey M-A, Hoq M, et al. Child and caregiver mental health during 12 months of the COVID-19 pandemic in Australia: findings from national repeated cross-sectional surveys. BMJ Paediatrics Open. 2022;6(1):e001390.
4. Poverty in Australia 2022: A snapshot is published by the Australian Council of Social Service, in partnership with UNSW Sydney.
5. Australian Early Development Census. An Australian Government Initiative. https://www.aedc.gov.au/.
6. Brinkman S, Gregory T, Harris J, et al. Associations between the early development instrument at age 5, and reading and numeracy skills at ages 8, 10 and 12: a prospective linked data study. Child Indicators Research. 2013;6(4):695-708.
7. Goldfeld S, O'Connor M, Chong S, et al. The impact of multidimensional disadvantage over childhood on developmental outcomes in Australia. International Journal of Epidemiology. 2018;47(5):1485-96.
8. The Health Foundation (2017). Healthy lives for people in the UK. Strategic plan. https://www.health.org.uk/publications/healthy-lives-for-people-in-the-uk.
9. Garg A, Cull W, Olson L, et al. Screening and Referral for Low-Income Families' Social Determinants of Health by US Pediatricians. Acad Pediatr. 2019;19(8):875-83.
10. Loveday S, Hall T, Constable L, et al. Screening for Adverse Childhood Experiences in Children: A Systematic Review. Pediatrics. 2022;149(2).
11. Hernanz V, Malherbet F, Pellizzari M. Take-Up of Welfare Benefits in OECD Countries: A Review of the Evidence. Economics. 2004;https://doi.org/10.1787/525815265414.
12. Scutella R, Johnson DT. Understanding and Improving Data Quality Relating to Low-Income Households. FaHCSIA Policy Research Paper. 2005(24).
13. Wiggan J, Talbot C. The benefits of welfare rights advice: a review of the literature. National Association of Welfare Rights Advisors. 2006.
14. Price AMH, Zhu A, Nguyen HNJ, et al. Study protocol for the Healthier Wealthier Families (HWF) pilot randomised controlled trial: testing the feasibility of delivering financial counselling to families with young children who are identified as experiencing financial hardship by community-based nurses. BMJ Open. 2021;11(5):e044488.
15. Burley J, Samir N, Price A, et al. Connecting Healthcare with Income Maximisation Services: A Systematic Review on the Health, Wellbeing and Financial Impacts for Families with Young Children. Int J Environ Res Public Health. 2022;19(11).
16. Naven L, Egan J. Healthier, Wealthier Children: Learning from an Early Intervention Child Poverty Project; GCPH: Glasgow, UK. 2013.
17. Naven L, Withington R, Egan J. Maximising opportunities: final evaluation report of the Healthier, Wealthier Children (HWC) project. Glasgow: Glasgow Centre for Population Health.; 2012.
18. NHS Greater Glasgow and Clyde. Healthier Wealthier Children. https://www.nhsggc.scot/your-health/public-health/maternal-and-child-public-health/healthier-wealthier-children/.
19. The Consolidated Framework for Implementation Research (CFIR). Ann Arbor: CFIR, 2019. Available from the CFIR website https://cfirguide.org/constructs/.
20. Honisett S, Loftus H, Liu H, et al. Do Australian policies enable a primary health care system to identify family adversity and subsequently support these families—A scoping study. Health Promotion Journal of Australia. 2023;34(1):211-21.

For peer review only - http://bmjopen.bmj.com/site/about/guidelines.xhtml

21. Chambers DA, Glasgow RE, Stange KC. The dynamic sustainability framework: addressing the paradox of sustainment amid ongoing change. Implement Sci. 2013;8:117.
22. Bonevski B, Randell M, Paul C, et al. Reaching the hard-to-reach: a systematic review of strategies for improving health and medical research with socially disadvantaged groups. BMC medical research methodology. 2014;14(1):1-29.
23. Hooker L, Taft A. Who is Being Screened for Intimate Partner Violence in Primary Care Settings? Secondary Data Analysis of a Cluster Randomised Trial. Maternal and Child Health Journal. 2021;25(10):1554-61.
24. Teager W, Fox S, Stafford N. How Australia can invest early and return more: A new look at the $15b cost and opportunity. Early Intervention Foundation, The Front Project and CoLab at the Telethon Kids Institute, Australia.; 2019.
25. Singh G, Zhu H, Cheung CR. Public health for paediatricians: Fifteen-minute consultation on addressing child poverty in clinical practice. Archives of disease in childhood - Education & practice edition. 2021;106(6):326-32.
26. Axford N, Berry V. Money Matters: Time for Prevention and Early Intervention to Address Family Economic Circumstances. Journal of Prevention. 2023.
27. Teare MD, Dimairo M, Shephard N, et al. Sample size requirements to estimate key design parameters from external pilot randomised controlled trials: a simulation study. Trials. 2014;15(1):264.
28. Melbourne Institute: Applied Economics & Social Research. The Household, Income and Labour Dynamics In Australia Survey: Selected Findings from Waves 1 to 17. Report. Melbourne, Australia: The University of Melbourne, Melbourne Institute; 2019.
29. Australian Institute of Family Studies. (2018). Longitudinal Study of Australian Children Data User Guide – December 2018. Melbourne: Australian Institute of Family Studies. Accessed from https://growingupinaustralia.gov.au/data-and-documentation/data-user-guide.
30. Saunders P, Zhu A. Comparing Disadvantage and Well-being in Australian Families [online]. Australian Journal of Labour Economics. 2009;12(No. 1):21-39.
31. Sollis K. Measuring child deprivation and opportunity in Australia: applying the Nest Framework to develop a measure of deprivation and opporutnity for children using the Longitudinal Study of Australian Children. Canberra; 2019 February.
32. Raat H, Botterweck AM, Landgraf JM, et al. Reliability and validity of the short form of the child health questionnaire for parents (CHQ-PF28) in large random school based and general population samples. J Epidemiol Community Health. 2005;59(1):75-82.
33. Waters E, Salmon L, Wake M, et al. The Child Health Questionnaire in Australia: reliability, validity and population means. Australian and New Zealand Journal of Public Health. 2000;24(2):207-10.
34. Group TE. EuroQol-a new facility for the measurement of health-related quality of life. Health policy. 1990;16(3):199-208.
35. Viney R, Norman R, King MT, et al. Time trade-off derived EQ-5D weights for Australia. Value in Health. 2011;14(6):928-36.
36. Henry JD, Crawford JR. The short-form version of the Depression Anxiety Stress Scales (DASS-21): construct validity and normative data in a large non-clinical sample. British Journal of Clinical Psychology. 2005;44(2):227-39.
37. Price AMH, Middleton M, Matthey S, et al. A comparison of two measures to screen for mental health symptoms in pregnancy and early postpartum: the Matthey Generic Mood Questionnaire and the Depression, Anxiety, Stress Scales short-form. Journal of Affective Disorders. 2021;281:824-33.
38. Cummins R. International Well Being Group: Personal Wellbeing Index. Melbourne: Australian Centre on Quality of Life, Deakin University. 2004.
39. Tong A, Sainsbury P, Craig J. Consolidated criteria for reporting qualitative research (COREQ): a 32-item checklist for interviews and focus groups. International Journal for Quality in Health Care. 2007;19(6):349-57.
40. Braun V, Clarke V. To saturate or not to saturate? Questioning data saturation as a useful concept for thematic analysis and sample-size rationales. Qualitative Research in Sport, Exercise and Health. 2021;13(2):201-16.

For peer review only - http://bmjopen.bmj.com/site/about/guidelines.xhtml

41. QSR International Pty Ltd. (2018). NVivo (Version 12). [Computer Software]. https://www.qsrinternational.com/nvivo-qualitative-data-analysis-software/home [Internet].
42. Phillips B, Gray M, Biddle N. COVID-19 JobKeeper and JobSeeker impacts on poverty and housing stress under current and alternative economic and policy scenarios. Acton, ACT: Australian National University Centre for Social Research and Methods; 2020.
43. Cassells R, Duncan A. JobKeepers and JobSeekers: How many workers will lose and how many will gain? Bentley, WA: Curtin University; 2020.
44. Gandy S. Care expands for moms, babies. Nursing & Allied Healthweek. 1996;1(13):1.
45. Klapdor M. Parliament of Australia. COVID-19 Economic response—free child care. https://www.aph.gov.au/About_Parliament/Parliamentary_Departments/Parliamentary_Library/FlagPost/2020/April/Coronavirus_response-Free_child_care [Accessed 26 March 2021]. 2020.
46. Davidson P. A tale of two pandemics: COVID, inequality and poverty in 2020 and 2021 ACOSS/UNSW Sydney Poverty and Inequality Partnership, Build Back Fairer Series, Report No. 3, Sydney. 2022.

For peer review only

AUTHOR CONTRIBUTIONS

AP Conceptualization, Methodology, Validation, Formal analysis, Investigation, Resources, Data Curation, Writing - Original Draft Preparation, Writing - Review & Editing, Visualization, Supervision, Project administration, Funding acquisition

NW Conceptualization, Methodology, Software, Validation, Formal analysis, Investigation, Data Curation, Writing - Review & Editing, Supervision, Project administration

JB Methodology, Validation, Formal analysis, Investigation, Resources, Data Curation, Writing - Review & Editing, Project administration, Funding acquisition

AZ Conceptualization, Methodology, Writing - Review & Editing, Funding acquisition

DCS Conceptualization, Methodology, Writing - Review & Editing

SW Investigation, Writing - Review & Editing, Project administration

MS Methodology, Validation, Investigation, Resources, Data Curation, Writing - Review & Editing, Project administration

KT Methodology, Validation, Investigation, Resources, Data Curation, Writing - Review & Editing, Project administration

MM Methodology, Validation, Investigation, Resources, Writing - Review & Editing, Project administration

JC Methodology, Validation, Investigation, Resources, Data Curation, Writing - Review & Editing, Project administration

RB Methodology, Investigation, Resources, Data Curation, Writing - Review & Editing, Project administration

SC Methodology, Validation, Investigation, Resources, Data Curation, Writing - Review & Editing, Project administration

LB Methodology, Validation, Investigation, Resources, Data Curation, Writing - Review & Editing, Project administration

DS Methodology, Validation, Investigation, Resources, Data Curation, Writing - Review & Editing, Project administration

RR Methodology, Validation, Investigation, Resources, Data Curation, Writing - Review & Editing, Project administration

AW Methodology, Validation, Formal analysis, Investigation, Data Curation, Writing - Review & Editing

NS Validation, Formal analysis, Investigation, Writing - Review & Editing, Project administration

RG Resources, Writing - Review & Editing

SR Methodology, Resources, Writing - Review & Editing

LK Methodology, Writing - Review & Editing

RL Methodology, Resources, Writing - Review & Editing, Funding Acquisition

VE Resources, Writing - Review & Editing, Funding Acquisition

For peer review only
For peer review only - http://bmjopen.bmj.com/site/about/guidelines.xhtml

SW Conceptualization, Methodology, Resources, Writing - Review & Editing, Supervision, Funding acquisition

SG Conceptualization, Methodology, Resources, Writing - Review & Editing, Supervision, Funding acquisition

All authors made substantial contributions to the conception or design of the paper; drafted this paper or revised it critically for important intellectual content; approved the final version submitted; and agree to be accountable for all aspects of the research.

ACKNOWLEDGEMENTS

We thank all families, children, Child and Family Health providers, and financial wellbeing providers who collaborated on Healthier Wealthier Families (HWF). We thank members of the Australian and International HWF Advisory Groups for their oversight and guidance, especially the team at Glasgow Centre for Population Health. We thank contributors who donated time in-kind over and above the project resources which made the pilot possible, and to the funders for their flexibility around project deliverables during the COVID-19 pandemic. The HWF pilot collaboration is led by the Centre for Community Child Health and BEST START-SW, in partnership with Maternal and Child Health Services, Child and Family Health Services, Uniting Vic.Tas, Upper Murray Family Care, Wesley Mission, the Melbourne Institute: Applied Economic & Social Research, the University of Melbourne, RMIT University, Western Sydney University, and the University of New South Wales.

For peer review only

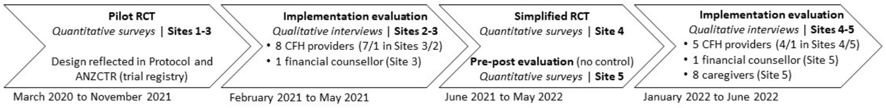

Figure 1. Healthier Wealthier Families data collection phases and research designs.

338x190mm (96 x 96 DPI)

Supplementary Figure 1. Participant flow for caregivers (CONSORT)

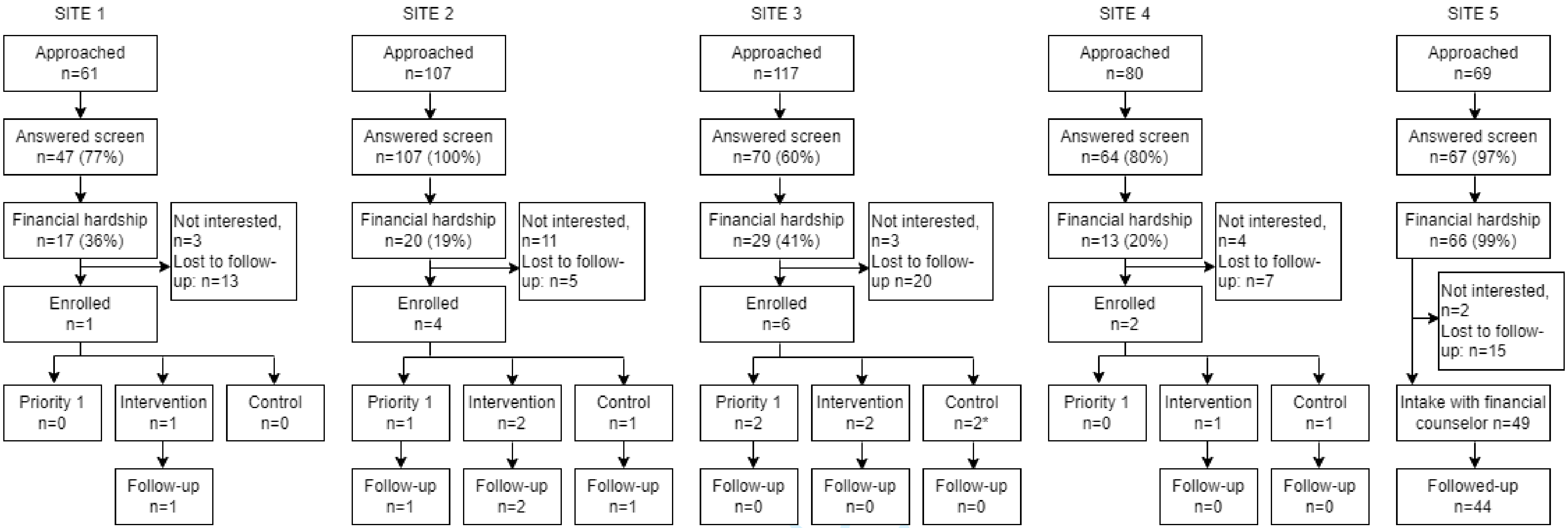

*In Site 3, the n=2 caregivers allocated to the Control arm were subsequently identified as high priority and referred to financial counselling.

Reporting checklist for randomised trial.

		Reporting Item	Page Number
**Title and Abstract**			
Title	#1a	Identification as a randomized trial in the title.	1[1]
Abstract	#1b	Structured summary of trial design, methods, results, and conclusions	2
**Introduction**			
Background and objectives	#2a	Scientific background and explanation of rationale	4
Background and objectives	#2b	Specific objectives or hypothesis	5
**Methods**			
Trial design	#3a	Description of trial design (such as parallel, factorial) including allocation ratio.	6, Table 1
Trial design	#3b	Important changes to methods after trial commencement (such as eligibility criteria), with reasons	7, Table 1
Participants	#4a	Eligibility criteria for participants	Table 1
Participants	#4b	Settings and locations where the data were collected	6
Interventions	#5	The experimental and control interventions for each group with sufficient details to allow replication, including how and when they were actually administered	Table 1
Outcomes	#6a	Completely defined prespecified primary and secondary outcome measures, including how and when they were assessed	Table 1
Outcomes	#6b	Any changes to trial outcomes after the trial commenced, with reasons	Table 1
Sample size	#7a	How sample size was determined.	Table 1
Sample size	#7b	When applicable, explanation of any interim analyses and stopping guidelines	n/a
Randomization - Sequence generation	#8a	Method used to generate the random allocation sequence.	Table 1
Randomization - Sequence generation	#8b	Type of randomization; details of any restriction (such as blocking and block size)	Table 1
Randomization - Allocation concealment mechanism	#9	Mechanism used to implement the random allocation sequence (such as sequentially numbered containers), describing any	Table 1

[1] NB, originally designed as an RCT, which was infeasible, the design changed throughout the study and the title reflects this with 'mixed methods'.

For peer review only

		Reporting Item	Page Number
		steps taken to conceal the sequence until interventions were assigned	
Randomization - Implementation	#10	Who generated the allocation sequence, who enrolled participants, and who assigned participants to interventions	Table 1
Blinding	#11a	If done, who was blinded after assignment to interventions (for example, participants, care providers, those assessing outcomes) and how.	Table 1
Blinding	#11b	If relevant, description of the similarity of interventions	Table 1
Statistical methods	#12a	Statistical methods used to compare groups for primary and secondary outcomes	Table 1
Statistical methods	#12b	Methods for additional analyses, such as subgroup analyses and adjusted analyses	n/a
**Results**			
Participant flow diagram (strongly recommended)	#13a	For each group, the numbers of participants who were randomly assigned, received intended treatment, and were analysed for the primary outcome	Supp Figure 1
Participant flow	#13b	For each group, losses and exclusions after randomization, together with reason	8, Table 3
Recruitment	#14a	Dates defining the periods of recruitment and follow-up	Table 1, Figure 1
Recruitment	#14b	Why the trial ended or was stopped	6 (Site 1)
Baseline data	#15	A table showing baseline demographic and clinical characteristics for each group	Supp Table 2
Numbers analysed	#16	For each group, number of participants (denominator) included in each analysis and whether the analysis was by original assigned groups	8, Supp Figure 1
Outcomes and estimation	#17a	For each primary and secondary outcome, results for each group, and the estimated effect size and its precision (such as 95% confidence interval)	n/a
Outcomes and estimation	#17b	For binary outcomes, presentation of both absolute and relative effect sizes is recommended	n/a
Ancillary analyses	#18	Results of any other analyses performed, including subgroup analyses and adjusted analyses, distinguishing pre-specified from exploratory	n/a
Harms	#19	All important harms or unintended effects in each group (For specific guidance see CONSORT for harms)	n/a
**Discussion**			
Limitations	#20	Trial limitations, addressing sources of potential bias, imprecision, and, if relevant, multiplicity of analyses	14

For peer review only

		Reporting Item	Page Number
Generalisability	#21	Generalisability (external validity, applicability) of the trial findings	14
Interpretation	#22	Interpretation consistent with results, balancing benefits and harms, and considering other relevant evidence	13-14
Registration	#23	Registration number and name of trial registry	2
**Other information**			
Interpretation	#22	Interpretation consistent with results, balancing benefits and harms, and considering other relevant evidence	13-15
Registration	#23	Registration number and name of trial registry	2
Protocol	#24	Where the full trial protocol can be accessed, if available	3
Funding	#25	Sources of funding and other support (such as supply of drugs), role of funders	3

None The CONSORT checklist is distributed under the terms of the Creative Commons Attribution License CC-BY. This checklist can be completed online using https://www.goodreports.org/, a tool made by the EQUATOR Network in collaboration with Penelope.ai

For peer review only

BMJ Open

The feasibility of linking universal child and family healthcare and financial counselling: findings from the Australian Healthier Wealthier Families (HWF) mixed methods study

Journal:	*BMJ Open*
Manuscript ID	bmjopen-2023-075651.R1
Article Type:	Original research
Date Submitted by the Author:	03-Oct-2023
Complete List of Authors:	Price, Anna; Murdoch Children's Research Institute, Centre for Community Child Health; The University of Melbourne, Department of Paediatrics<br>White, Natalie; Murdoch Children's Research Institute, Centre for Community Child Health<br>Burley, Jade; Sydney Children's Hospitals Network Randwick; University of New South Wales, Women's and Children's Health<br>Zhu, Anna; RMIT University, School of Economics, Marketing and Finance<br>Contreras-Suarez, Diana; The University of Melbourne, Melbourne Institute: Applied Economic & Social Research<br>Wang, Si; University of New South Wales, Psychiatry and Mental Health/ School of Clinical Medicine<br>Stone, Melissa; Uniting Vic.Tas<br>Trotter, Kellie; Hume City Council, Hume Enhanced Maternal and Child Health<br>Mrad, Mona; Uniting Vic.Tas<br>Caldwell, Jane; City of Wodonga, Wodonga Enhanced Maternal and Child Health Service<br>Bishop, Rebecca; Wesley Mission<br>Chota, Sumayya; Wesley Mission<br>Bui, Lien; Child and Family Health Services<br>Sanger, Debbie; Child and Family Health Services<br>Roles, Rob; Uniting Vic.Tas<br>Watts, Amy; Murdoch Children's Research Institute, Centre for Community Child Health<br>Samir, Nora; University of New South Wales - Randwick Campus, School of Women's and Children's Health<br>Grace, Rebekah; Western Sydney University, Centre for the Transformation of early Education and Child Health; Ingham Institute, BestSTART-South West<br>Raman, Shanti; Ingham Institute, BestSTART-SWS; South Western Sydney Local Health District, Community Paediatrics<br>Kemp, Lynn; Western Sydney University, Ingham Institute; Western Sydney University, Translational Research and Social Innovation (TReSI)<br>Lingam, Raghu; University of New South Wales, School of Women and Children’s Health; Sydney Children's Hospitals Network Randwick<br>Eapen, Valsamma; Ingham Institute, BestSTART-SWS; University of New South Wales, Psychiatry and Mental Health/ School of Clinical Medicine

	Woolfenden, Susan ; Local Government and Shires Association of NSW; University of New South Wales,<br>Goldfeld, Sharon; Murdoch Children's Research Institute, Centre for Community Child Health; The University of Melbourne, Paediatrics
**Primary Subject Heading**:	Health services research
Secondary Subject Heading:	Health services research, Public health
Keywords:	MENTAL HEALTH, Community child health < PAEDIATRICS, Primary Health Care

SCHOLARONE™
Manuscripts

I, the Submitting Author has the right to grant and does grant on behalf of all authors of the Work (as defined in the below author licence), an exclusive licence and/or a non-exclusive licence for contributions from authors who are: i) UK Crown employees; ii) where BMJ has agreed a CC-BY licence shall apply, and/or iii) in accordance with the terms applicable for US Federal Government officers or employees acting as part of their official duties; on a worldwide, perpetual, irrevocable, royalty-free basis to BMJ Publishing Group Ltd ("BMJ") its licensees and where the relevant Journal is co-owned by BMJ to the co-owners of the Journal, to publish the Work in this journal and any other BMJ products and to exploit all rights, as set out in our licence.

The Submitting Author accepts and understands that any supply made under these terms is made by BMJ to the Submitting Author unless you are acting as an employee on behalf of your employer or a postgraduate student of an affiliated institution which is paying any applicable article publishing charge ("APC") for Open Access articles. Where the Submitting Author wishes to make the Work available on an Open Access basis (and intends to pay the relevant APC), the terms of reuse of such Open Access shall be governed by a Creative Commons licence – details of these licences and which Creative Commons licence will apply to this Work are set out in our licence referred to above.

Other than as permitted in any relevant BMJ Author's Self Archiving Policies, I confirm this Work has not been accepted for publication elsewhere, is not being considered for publication elsewhere and does not duplicate material already published. I confirm all authors consent to publication of this Work and authorise the granting of this licence.

For peer review only

The feasibility of linking universal child and family healthcare and financial counselling: findings from the Australian Healthier Wealthier Families (HWF) mixed methods study

Dr Anna Price*[1,2,3], Ms Natalie White[1,2], Dr Jade Burley[4,5,6,7], Dr Anna Zhu[8], Dr Diana Contreras-Suarez[9], Dr Si Wang[10], Melissa Stone[11], Kellie Trotter[12], Mona Mrad[11], Dr Jane Caldwell[13], Rebecca Bishop[14], Sumayya Chota[15], Lien Bui[16], Debbie Sanger[17], Rob Roles[18], Dr Amy Watts[1,2], Ms Nora Samir[4,5,6], Prof Rebekah Grace[6,19,21], A/Prof Shanti Raman[6,20], Prof Lynn Kemp[6,19,21,22], Prof Raghu Lingam[4,5,6], Prof Valsamma Eapen[6,10], **Prof Susan Woolfenden[5,6,23], **Prof Sharon Goldfeld[1,2,3]

1 Centre for Community Child Health, The Royal Children's Hospital, Parkville, VIC 3052, Australia
2 Population Health, Murdoch Children's Research Institute, Parkville, VIC 3052, Australia
3 Department of Paediatrics, The University of Melbourne, Parkville, VIC 3052, Australia
4 Sydney Children's Hospital Network, Sydney, NSW 2031, Australia
5 Population Child Health Research Group, School of Women and Children's Health, University of NSW, Randwick, NSW 2031, Australia
6 BestSTART-SWS, Ingham Institute for Allied Medical Research, Liverpool, NSW 2170, Australia.
7 Centre of Excellence for The Digital Child, The University of Wollongong, Wollongong, NSW 2522, Australia
8 School of Economics, Marketing and Finance, RMIT University, Melbourne, VIC 3000, Australia
9 Melbourne Institute, Applied Economic & Social Research, University of Melbourne, Parkville, VIC 3010, Australia
10 Academic Unit of Infant, Child and Adolescent Services (AUCS), Psychiatry and Mental Health/ School of Clinical Medicine, UNSW Medicine & Health, NSW 2170, Sydney, Australia
11 Uniting Vic.Tas, Epping VIC 3076, Australia
12 City of Hume Enhanced Maternal Child Health Service, Hume VIC 3064, Australia
13 City of Wodonga Enhanced Maternal and Child Health Service, Wodonga VIC 3689, Australia
14 Wesley Mission, Sydney, NSW 2000 Australia
15 Wesley Mission, Fairfield, NSW 2165 Australia
16 Child and Family Health, Fairfield NSW 2165 Australia
17 Child and Family Health, Albury Community Health, Albury NSW 2640, Australia
18 Uniting Vic.Tas, Broadmeadows VIC 3047, Australia
19 Transforming early Education and Child Health (TeEACH) Research Centre, Western Sydney University, Westmead, NSW 2560, Australia
20 Community Paediatrics, South Western Sydney Local Health District, Liverpool, NSW 2170, Australia
21 Translational Health Research Institute, Western Sydney University, Penrith South, NSW 2750, Australia
22 Translational Research and Social Innovation (TReSI), School of Nursing and Midwifery, Western Sydney University, Penrith South, NSW 2750, Australia
23 Sydney Medical School, University of Sydney, NSW 2006, Australia

*Corresponding author email: anna.price@mcri.edu.au
**Joint senior authors

For peer review only - http://bmjopen.bmj.com/site/about/guidelines.xhtml

ABSTRACT

Objectives: "Healthier Wealthier Families" (HWF) seeks to reduce financial hardship in the early years by embedding a referral pathway between Australia's universal child and family health (CFH) services and financial counselling. This pilot study investigated the feasibility and short-term impacts of HWF, adapted from a successful Scottish initiative.

Methods: Setting: CFH services in five sites across two states, coinciding with the COVID-19 pandemic. Participants: Caregivers of children aged 0-5 years experiencing financial hardship (study-designed screen). Design: Mixed methods. With limited progress using a randomised trial (RCT) design in Sites 1-3 (March 2020-November 2021), qualitative interviews with service providers identified implementation barriers including stigma, lack of knowledge of financial counselling, low financial literacy, research burden and pandemic disruption. This informed a simplified RCT protocol (Site 4) and direct referral model (no randomisation, pre-post evaluation, Site 5) (June 2021 to May 2022). Intervention: Financial counselling; Comparator: Usual care (Sites 1-4). Feasibility measures: proportions of caregivers screened, enrolled, followed-up, and who accessed financial counselling. Impact measures: finances (quantitative) and other (qualitative) to 6 months post-enrolment.

Results: 355/434 caregivers completed the screen (60%-100% across sites). In RCT sites (1-4), 79/365 (19%-41%) reported hardship but less than one-quarter enrolled. In Site 5, n=66/69 (96%) caregivers reported hardship and 44/66 (67%) engaged with financial counselling; common issues were utility debts (73%), and obtaining entitlements (43%) or material aid/emergency relief (27%). Per family, financial counselling increased income from government entitlements by an average $6,504 annually plus $784 from concessions, grants, brokerage, and debt waivers. Caregivers described benefits (qualitative) including reduced stress, practical help, increased knowledge, and empowerment.

Conclusions: Financial hardship screening via CFH was acceptable to caregivers, direct referral was feasible, but individual randomisation was infeasible. Larger-scale implementation will require careful, staged adaptations where CFH populations and the intervention are well-matched, and low burden evaluation.

Registration: ACTRN12620000154909.

For peer review only

Strengths and limitations of this study

- The iterative approach to piloting in five sites across two Australian states, and during the COVID-19 pandemic, enabled identification of design components that contributed to feasibility.
- The study prioritised families who are often excluded from research and used a pragmatic and high quality mixed methods approach.
- Limitations included small sample sizes, short-term follow-up, lack of control group, and lack of quantitative data on non-financial impacts.
- The lessons from this pilot can inform planning for implementation and evaluation at scale, to maximise the potential benefits generated by the model.
- It is anticipated that the HWF model would be more feasible in the post-pandemic context, as financial supports ended, and cost-of-living pressures increased.

Original protocol: http://dx.doi.org/10.1136/bmjopen-2020-044488

Keywords: child development, poverty, mental health, parenting, community child health.

For peer review only

1. INTRODUCTION

Strengthening financial security can buffer families from early adversity and enhance the environments that help children thrive.[1] During the first 1000 days of life (conception to 2 years), the human brain develops more rapidly than at any other time, laying the foundation for ongoing health and development.[2] Financial hardship can disrupt this foundational period.[1,2] In Australia, a high-income country with universal health, education and social services, one in six children live below the poverty line, defined as 50% of median income using the traditional government measure.[3,4] One in three children experience material deprivation, missing out on essential items such as food and healthcare due to cost.[3] Even by the time Australian children start school, developmental inequities due to adverse social conditions are evident.[5] Children living in the least advantaged suburbs have three times the developmental vulnerability of the most (19% versus 7%).[5] Early adversity translates to increased risks of poorer socioemotional functioning, school failure, chronic disease, mental illness, reduced economic opportunity, and intergenerational adversity.[2,6] By adopting policies and practices that address early adverse conditions, it is estimated that Australia could reduce poor developmental outcomes in middle childhood by 50%-70%.[7]

While the mechanisms are complex, it is understood that increased household income benefits children directly through better food, stable housing, and healthcare (the investment model), as well as indirectly through improving parental mental health and thus parental capacity (the family stress model).[1] Healthcare offers avenues for addressing financial hardship; yet, this remains a perennial challenge for services that are designed to treat health symptoms but not their social causes.[8-10] In high-income countries with universal welfare platforms such as Australia and the United Kingdom (UK), government assistance is available to ease financial stress. However, low take-up of benefits is common, with an estimated 40%-80% of eligible recipients missing out.[11] 1.3 million Australians were found to be missing out on benefits in 2004,[12] when this issue was last publicly addressed. Common explanations for not receiving benefits include lack of awareness, system complexity, and stigma.[13]

Across many high-income countries, financial counselling services are freely available to help people overcome barriers to accessing entitlements and other financial supports, typically funded by governments. In Australia, however, services remain embedded in not-for-profit organisations and unconnected to the broader system.[14] Our systematic review of health-financial linkage models for families with children aged 0-5 years identified only four international models, with limited randomised trial-level evidence.[15] Substantial benefits were demonstrated by a Scottish referral model ("Healthier Wealthier Children"), which engaged health workers in early years services to identify and refer eligible caregivers to money advice workers. Two pre-post evaluations conducted between 2010 and 2013 reported average annual financial gains per caregiver of £1661 (n=2516) and

For peer review only - http://bmjopen.bmj.com/site/about/guidelines.xhtml

£1919 (n=2289).[16,17] Positive spill-over effects included improved health, housing and quality of life. By 2020, 10 years after it began, it had generated 27,000 referrals and over £36 million in financial gain, and is now a requirement of Scotland's Child Poverty action plan.[18]

The current study, "Healthier Wealthier Families" (HWF), sought to adapt the Scottish model to the Australian service context and evaluate the model via a pilot randomised controlled trial (RCT).[14] Specific aims were to test the (1) feasibility of the HWF referral model and (2) short-term impacts on household finances, caregiver health, parenting efficacy and use of financial services. The overarching goal was to inform the methods and resourcing for a definitive, multisite effectiveness trial that would test whether the HWF model could reduce financial hardship for Australian families when delivered at scale.

2. METHODS AND ANALYSIS

2.1 Setting

Australia's universal Child and Family Health (CFH) services offer free, community-based healthcare to families with children from birth to school entry. Available in all Australian jurisdictions, CFH services are delivered by nurses, often in partnership with allied health providers, and working with interpreters as needed. The services are mostly clinic-based and offer broad ranging support for children's health, development, and parental wellbeing. In some jurisdictions, CFH services offer higher intensity support (more, longer, and home-based appointments) for families experiencing increased health risks and social adversity. These service streams are differentiated with the labels 'universal' and 'higher intensity' throughout.

Australian CFH services already identify and respond to adverse experiences such as poor parental mental health and family violence. While the service frameworks recognise financial hardship as a social adversity affecting child and family outcomes, there is a gap in practice in systematically identifying and responding to this need. The HWF pilot was tested with CFH services in lower socioeconomic communities in Australia's two most populous states, Victoria and New South Wales (NSW). The study was planned before the COVID-19 pandemic, but implementation and evaluation occurred during the pandemic and were deeply impacted by Australia's public health restrictions and economic responses. In brief, stay-at-home orders (lockdown) were Australia's main measure of disease control until November 2021, when vaccination levels reached 70-80%. This required CFH and financial counselling services to shift to telehealth within weeks, and saw many CFH services seconded to other pandemic-related duties. To offset the economic fallout of lockdown, the government implemented a range of financial supplements and supports in April 2020. By September 2020, this financial response had reduced poverty and housing stress beyond pre-pandemic levels;

For peer review only - http://bmjopen.bmj.com/site/about/guidelines.xhtml

however, financial hardship had rebounded a year later after financial supports were withdrawn.[4] For more information on Australia's pandemic responses, please see the detailed description in Supplementary Material 1.

2.2 Design

When the HWF pilot study was registered with the Australian and New Zealand Controlled Trials Registry (ACTRN12620000154909, 13 February 2020), it was designed as a feasibility RCT in one Victorian site. However, implementation and evaluation during the COVID-19 pandemic required protocol iterations developed through continuous quality improvement, which incorporated quantitative and qualitative elements. When recruitment ended on 30 June 2022, we had attempted iterations of HWF in five sites across two states. Overall, the model was infeasible in Sites 1-4 and feasible in Site 5. Figure 1 shows the progression and design elements of the study. Recruitment began in Site 1 in March 2020, before pausing five weeks later in response to Australia's national lockdown (and eventually ended in 2021 without recommencing). In early-2020, partnerships were formed with Sites 2 and 3, reflected by updates to the trial registry (2 March 2021) and the published protocol (http://dx.doi.org/10.1136/bmjopen-2020-044488, accepted 4 May 2021).[14] Table 1 describes the RCT methods as published, following the CONSORT statement.[14]

To understand barriers to feasibility encountered in 2020, a qualitative implementation evaluation commenced in February 2021 (Figure 1), informed by the Consolidated Framework for Implementation Research (CFIR).[19] The theoretical framework was reflexive thematic analysis, and the methods are detailed in Table 2. Interviews with nine service providers in Sites 2 and 3 revealed three core themes, summarised below. They are described in detail with illustrative quotes in Supplementary Table 1. We include these results in the methods section to explain why we had to alter the design approach.

1. Experiences and perceptions of the screening and referral processes:
   - 1.1. CFH providers had differing opinions about the usefulness of financial screening and relevance to their own practice, which were related to delivery of universal or higher intensity services.
   - 1.2. There were issues with engagement due to the research processes.
   - 1.3. The COVID-19 pandemic affected service provision and levels of financial hardship.
2. Barriers to identifying financial hardship and accepting help included:
   - 2.1. Stigma surrounding financial hardship and accepting a referral to a financial counsellor.
   - 2.2. Financial counselling was not well understood by CFH providers or families.
   - 2.3. Clients in crisis may have other priorities.
   - 2.4. Financial literacy and cultural and family considerations.

For peer review only - http://bmjopen.bmj.com/site/about/guidelines.xhtml

3. Factors that enable engagement with families with the HWF model (classified as suggestions rather than sub-themes):
   - Support service providers to understand the role of financial counselling;
   - Provide resources to families about financial hardship and financial counselling.
   - Ensure accessible and inclusive communication.
   - Financial counselling services provide client-centred care, not service-led.

These findings informed iterations to the HWF protocol that were trialled with two new sites (Sites 4 and 5, commencing mid-late 2021, see Figure 1), described next.

2.3 Design iterations

For Site 4, the RCT protocol was simplified. Research processes such as informed consent and questionnaires were shortened, and follow-up was reduced from two caregiver surveys at 3 and 6 months to one at 6 months (see Table 1).

More substantive design changes were implemented for Site 5. Instead of an RCT, the model employed direct referral with a pre-post evaluation (see Table 1). Researcher-led processes were replaced with short, CFH-led consent and evaluation using data collected by financial counsellors rather than surveys collected by the research team. The individual randomisation was removed so there was no control group. This meant that rather than the researcher doing the referral, it was done by the CFH provider at the time of the screening survey. This made it possible for the research team to support the CFH and financial counselling providers to build trusted, working relationships with one another. The design also reflected how the referral pathway would operate in the real-world context, i.e. without a researcher intervening between screening and referral.

In January-June 2022, we conducted a second phase of the implementation evaluation to understand barriers to and enablers of feasibility in Sites 4-5 (Figure 1, methods in Table 2). This included interviews with four CFH providers in Site 4, and one CFH provider and one financial counsellor in Site 5 (no financial counsellors in Site 4 were interviewed because no caregivers engaged, see Results). The themes regarding feasibility aligned with those identified in the first phase of the implementation evaluation and summarised above, so they are synthesised together in Supplementary Table 1. We conducted interviews with eight caregivers in Site 5 who engaged with financial counselling to understand perceived impacts of the HWF model (methods described in Table 2). The results are presented as main findings in the next section. Due to the low levels of enrolment in Sites 1-4, no caregiver interviews were conducted.

2.4 Patient and Public Involvement

Patients and service providers were involved in the design and conduct of this research. During the feasibility stage, the choice and acceptability of financial hardship items was informed by discussions

For peer review only

with CFH providers and financial counsellors, and between CFH providers and their clients. During the implementation evaluation, the trial processes were reviewed with providers in Sites 2-5 and caregivers in Site 5. Once the trial is published, findings (in academic and lay outputs) will be available on the study website (https://www.rch.org.au/ccch/Healthier_wealthier_families/).

3. RESULTS

3.1 Feasibility (Aim 1)

3.1.1 Completion of the financial hardship screening survey

Table 3 summarises the feasibility measures by Site. The participant flow for caregivers is presented as a CONSORT diagram in Supplementary Figure 1. Across the five sites, CFH providers approached a total of n=434 caregivers. In the four sites where a small number of CFH providers did HWF screening each week, 75%-100% of caseloads were approached. In Site 4, where all 11 nurses did screening each week, 20% of the caseload was approached. Across the five sites, a total of n=355 caregivers completed the screening survey (ranging 60% to 100% of those approached). Reasons for declining screening were collected in Sites 1 and 5 and comprised: no interest or need for financial assistance (n=7), too personal (n=1) and no reason provided (n=8).

3.1.2 Disclosure of financial hardship (eligibility)

In the universal CFH service sites, Table 3 shows that n=17/47 (36%) caregivers in Site 1, 29/70 (41%) in Site 3, and 13/64 (20%) in Site 4 disclosed financial hardship. There was greater variation between the two sites delivering higher intensity CFH services: 20/107 (19%) in Site 2 compared with 66/67 (99%) in Site 5. The low proportion in Site 2 was at least partly due to the COVID-19 pandemic income supplements in 2020-21 reducing the levels of financial hardship (see CFH provider quotes in Supplementary Table 1 and Australian data published in [4]). Across the five sites, missing data on the financial screen were minimal, with only 8 missing values across the items and none missing for the hardship summary variable.

3.1.3 Consent/enrolment and follow-up in the study

Across the four RCT sites (1-4), 79 caregivers disclosed financial hardship and were thus eligible (Table 3, Supplementary Figure 1). Of these, 58 (73%) caregivers expressed interest in the study. Three caregivers were identified as high priority for asset loss or other social risk and immediately referred (see methods in Table 1); ten caregivers were allocated (6 intervention and 4 control); and 45/58 (78%) were lost to follow-up. Of 13 enrolled caregivers, 5 completed a follow-up questionnaire (all from Sites 1 and 2). In the direct referral model in Site 5, 64/67 caregivers were referred, 49/64

For peer review only - http://bmjopen.bmj.com/site/about/guidelines.xhtml

(77%) were contacted by the financial counsellor and 44/64 (69%) engaged with the service, which included having service provision (follow-up) data recorded and provided by the financial counsellor.

3.1.4 Caregivers who accessed, attended, and completed the intervention

In Site 3, the two caregivers who were allocated to the Control group subsequently identified as high priority and were referred to financial counselling. All 6 caregivers enrolled in Site 3 engaged with the financial counsellor, but no follow-up data were provided. Across the other RCT Sites, no other intervention or Priority 1 caregivers engaged with the financial counsellor. In Site 5, where there were 44 attending caregivers, all appointments were conducted by phone due to the COVID-19 pandemic restrictions. Two-thirds of caregivers used one appointment; 14% used two; 16% used three and 2% used four. Four caregivers missed one appointment and two caregivers missed two, and all were rescheduled. The financial counsellor spent an average of 1.9 hours (SD 1.1) in meetings with attending caregivers [range 0.5-4.75 hours], and an average of 2.2 hours (SD 2.2) outside of meetings on each caregiver's case [range 0.5-11 hours]. By 6 months post-referral, cases were fully closed for 32/44 (72%) caregivers and partially closed (mostly due to disengagement) for 12/44 (27.3%).

In Site 5, the most common issues facing caregivers were utility debts (32/44, 73%), awaiting government entitlements ('Centrelink', 19/44, 43%) and needing material aid or emergency relief (12/44, 27%). Eighteen percent of caregivers had borrowed from friends and families; other problematic debts and loans were less frequent. While no caregivers faced bankruptcy, half the caregivers disclosed Priority 1 indicators, such as imminent eviction or disconnection of utilities, or being threatened with legal action (n=17/44 (38.6%) for each category).

3.1.5 Outcome of feasibility

Overall, the feasibility measures for Sites 1-4 met definite Stop criteria for progressing to a larger scale evaluation (defined in Table 1) whereas for Site 5 they met definite Go criteria, noting that these were defined for a pilot RCT and not a pre-post evaluation.

3.2 Caregiver participant characteristics

Supplementary Table 2 presents the caregiver participant characteristics, noting that numbers in the RCT sites (1-4) are small and limit interpretation. Across the five sites, approximately 60% of caregivers who enrolled were partnered, and the number of children in their care ranged 1-7. Caregivers tended to have low levels of education and low household income (noting $50,000 per annum approximated the poverty line for a family with two adults and two children). While most enrolled caregivers were not in paid work (n=7/12 in Sites 1-4 and n=41/44 in Site 5), some expressed a desire for more employment (n=3/9 in Sites 1-4 and n=18/44 in Site 5). The enrolled caregivers in Sites 1-4 were more likely to have been born in Australia and speak English at home. In contrast, of the n=44 caregivers in Site 5 who engaged with financial counselling, three-quarters were born

For peer review only

overseas and just over half spoke languages other than English at home. Just under half lived with a household member with a disability or special health care needs. In Site 5, the small number of caregivers who were referred to financial counselling but lost to follow-up (n=5) appeared broadly similar to those who engaged.

3.3 Short-term impacts identified by quantitative evaluation (Aim 2)

3.3.1 Financial impacts

The lack of follow-up data from caregivers and financial counsellors in the RCT Sites 1-4 precludes the analysis described in the published Protocol [14]. For the 44 caregivers who engaged in Site 5, Table 4 shows that financial counselling established gains to household income including: (i) government benefits (Centrelink) totalling $11,007 per fortnight: an average of $355 (SD $447) for the n=31 caregivers specified; (ii) concessions totalling $12,700: an average of $508 for the 25 caregivers with amounts specified; (iii) grants totalling $7,330: an average of $564 (SD $404) for the 13 caregivers with amounts specified; (iv) brokerage totalling $1,680: an average of $129 for the 13 caregivers specified, and (v) a total of $12,800 debt waived for 3 caregivers. When these financial gains were averaged across the 44 caregivers, income from government entitlements increased $6,504 annually and families received payments totalling $784 from concessions, grants, brokerage, and debt waivers. Table 4 shows that other frequent activities included financial literacy (66%) and referrals to other agencies (41%), most commonly housing, employment, and emergency relief (see Table footnote).

3.3.2 Other (non-financial) impacts

As above, the lack of follow-up data across the RCT sites (1-4) precluded the analysis of non-financial impacts described in the published Protocol [14] and Supplementary Table 3. In Site 5, the Matthey Generic Mood Questionnaire (MGMQ) and Personal Wellbeing Index (PWI) were embedded into the financial counselling intake and follow-up surveys (described in Supplementary Table 3). They were subsequently added to the CFH screening from October 2021 after the first 37/69 caregivers were screened. As there were high levels of missing data on these measures, we describe them in text (not tabulated). To provide a consistent analytic sample, they are summarised for the 49 caregivers who did intake with the financial counsellor (relevant for 28 caregivers at CFH screening).

- At CFH screening:
  - 11/28 (39%) caregivers completed the MGMQ, of which 8/11 (73%) disclosed emotional distress (versus not).
  - Only 4/28 (8%) caregivers completed the PWI, mean 42.3 (standard deviation (SD) 4.9).
- At intake with the financial counsellor:

For peer review only - http://bmjopen.bmj.com/site/about/guidelines.xhtml

- 43/49 (88%) caregivers completed the MGMQ, of which 31/43 (72%) reported high emotional distress.
- 38/49 (78%) caregivers completed the PWI; mean 41.9 (SD 9.7).

- The follow-up survey was mostly completed by the financial counsellor based on client records and not in-person with the caregiver. As such, completion rates were low:
  - 7/49 (14%) caregivers completed the MGMQ; none reported emotional distress.
  - 11/49 (22%) caregivers completed the PWI; mean 57.2 (SD 11.2).

3.4 Short-term impacts identified by qualitative evaluation (Aim 2)

3.4.1 Caregiver characteristics (Site 5)

In Site 5, at the final appointment, the financial counsellor invited 39 caregivers to take part in the qualitative interview on behalf of the research team. Ten caregivers agreed and eight took part including one with an interpreter. Of the two who were not interviewed, one was unavailable at their scheduled time, and one was lost due to difficulties in finding an interpreter in their first language. Demographic characteristics were not collected due to ethical approval being only available for de-identified data sharing.

3.4.2 Caregiver perspectives (Site 5)

Caregivers who engaged with financial counselling were unanimous in finding the intervention beneficial. Four core themes (benefits) emerged and are presented with illustrative quotes, noting some overlap between themes.

<u>Theme 1</u>: Reduced stress and gratitude for emotional support and advocacy received.

Caregivers described their relationship with the financial counsellor as being empathetic and respectful. The supportive nature of this relationship helped reduce the acute sense of burden and isolation caregivers were experiencing.

> *"My financial counsellor was very supportive. She said you are not alone, there are people out there who are experiencing the same thing. So, she had a good talk to me, and after talking to her, I was able to put myself together." [CAREGIVER 4]*

Caregivers were grateful for the opportunity to share their problems with a trusted and supportive service provider who listened, and for the strong advocacy they received. They experienced unexpected relief from the sense of pressure and hopelessness they had been feeling before referral to the financial counsellor.

> *"… it's like you're sharing your feelings with someone. So I was just … I couldn't speak to anybody about it. So, she's the one who I am open with … open myself and explain my situation.*

For peer review only - http://bmjopen.bmj.com/site/about/guidelines.xhtml

And I got a very positive response from that time, so it was easy for me after that." [CAREGIVER 13]

Theme 2: Immediate solutions: crisis management and practical changes to organisation of finances.

Caregivers described several ways in which the financial counsellor assisted them. These included acting as a buffer between the caregiver and creditors; helping caregivers manage debt and bills, access entitlements to maximise their income and prevent a debt spiral; and supporting caregivers to prioritise bill payments according to their goals and needs.

"She was talking to me in a way that made me feel that she would take care of everything. 'Don't worry, I will have the payments divided into instalments' … She helped me organize myself. She showed me how to organize my bills and how to make payments." [CAREGIVER 2]

This proactive and practical support provided caregivers immediate reprieve.

"Because it was a big stress, for me, because always [Utility Company] send me notice – you're late to pay, you're late to pay. I can't pay anyway, I can't do it, and I was worry. You know, they send me always notice, notice … if they cut electricity, it's hard because winter, cold, kids. So hard … So, I was so happy when I get [help]." [CAREGIVER 8]

Theme 3: Increased knowledge and clarity.

Caregivers had increased awareness of the support and options available and that they were entitled to access. Caregivers described the personalised advice about finances provided by the financial counsellor.

"That's why it was really useful … she really helped with things that we have no idea existed. Can't find information on. So it was very informative." [CAREGIVER 3]

Caregivers valued the opportunity to ask questions when they did not understand something, and have their questions answered by a trusted service provider who understood their circumstances.

"What I felt when they helped me with how to make payments and how to organise myself. I told you; my parents are overseas. I thought it was like a family. My father teaching me how to make payments and how to organize myself. That's my feeling." [CAREGIVER 2]

Theme 4: Wellbeing, self-efficacy, and empowerment

Caregivers described improvements in their confidence and outlook, including more capacity and efficacy as a parent.

"But my social worker and financial counsellor has been able to uplift me to make that priority along with my family's needs and open my mind and eyes to see that everything has to be on time,

For peer review only - http://bmjopen.bmj.com/site/about/guidelines.xhtml

not to burden myself to make things worse. I know my daughter needs my attention, but I know now I have to do this first in order to be able to look after her the right way." [CAREGIVER 4]

Caregivers also described having increased control of their personal situation, more stability, and feeling less stranded and stressed.

"I just can say that it is very good that if woman like me will be aware that you can raise your voice and there is help … we should have more resources to have knowledge that we can go to this way. I think it's the best way if … we have more awareness for the woman who they are like me … that there is help. No matter what situation you are in, there is help." [CAREGIVER 5]

For peer review only

4. DISCUSSION

This Australian pilot study aimed to test the feasibility and short-term impacts of a systematic referral pathway between universal Child and Family Health (CFH) and financial counselling services. While financial hardship screening by CFH providers was broadly acceptable to caregivers of young children, a feasible referral model was established in only one of five sites. Challenges to implementation were exacerbated by the COVID-19 pandemic. Supplementary Figure 2 summarises the key elements, enabling ingredients and benefits of HWF. Feasibility was enabled when screening was viewed by CFH providers as adding value to practice; direct referral was embedded between services; research activities were minimal; strong working relationships between services were enabled by the research team; CFH providers conducted longer, home-based visits; and financial hardship was highly prevalent. Where the feasible model was developed, financial counselling created average gains in household income from government benefits of $125 p/week, plus additional payments totalling $784. This is substantial given that 80% of households earned less than $1,000 per week (which approximated the Australian government's poverty line for a family with two adults and two children). While we were unable to collect quantitative data on non-financial benefits, in qualitative interviews caregivers described benefits including reduced stress and gratitude, accessing immediate solutions to their financial issues, increased knowledge and clarity, and wellbeing, self-efficacy, and empowerment.

Australian policies are increasingly recognising the role of healthcare in addressing early adversities;[20] however, there is a dearth of research in Australia and internationally on the implementation and benefits of asking about early adversity in healthcare.[9,10] In the three pilot sites delivering universal CFH services, the 20%-41% of caregivers disclosing financial hardship was comparable with Australian population level data (also collected in 2020-21) showing that one in three families could not afford essential items.[3] In the feasible HWF model (Site 5), two-thirds of eligible caregivers engaged with financial counselling and the financial gains and sources (e.g.

predominately government benefits) were similar to those in the Scottish evaluations, where engagement ranged 45%-54%.[16,17] The Scottish history offers a way forward for extending the potential of the Australian model, such as engaging other early years services (e.g. antenatal or early education and care) and modifying the delivery of financial counselling through co-located or outreach services.[16]

Strengths of this study included the iterative, dynamic approach to piloting in five sites across two Australian states. Adaptation of the protocol during the COVID-19 pandemic enabled identification of design components that contributed to feasibility. Recognised by the dynamic sustainability framework,[21] this approach is integral to achieving feasible, impactful implementation – and is core to the sustainability of the Scottish program. Our study prioritised families who are often excluded from research [22] and used a pragmatic and high quality mixed methods approach. The qualitative implementation evaluation was critical for understanding the experiences of caregivers and providers, and together with the costs and resourcing used for the pilot, can inform the development and implementation of financial screening via healthcare on a larger scale in Australia and other high-income countries with comparable service systems.

Limitations include the small sample sizes and limited demographic data for describing the qualitative cohorts; the short-term follow-up; lack of control group; and lack of quantitative data on non-financial impacts. In Site 5, the pre-post evaluation could have provided a self-control design if pre-enrolment experiences of financial activities (e.g. counselling, use of *MoneySmart*, legal aid) were measured. Further, the high levels of missing data on emotional wellbeing measures reflected the difficulty in embedding new measures into CFH and financial counselling practice. Indeed, the latter does not typically inquire about caregivers' mental health needs because it can require a therapeutic response that is outside the scope of practice. Of the available data, there were high levels of emotional distress (low wellbeing) at screening and intake. While it was reassuring that the available measures (MGMQ and PWI) demonstrated higher emotional wellbeing at follow-up than intake, the sample sizes were too small to draw conclusions. The substantive challenges encountered in developing a feasible model of HWF (Sites 1-4), and collecting quantitative data on non-financial impacts to enable evaluation of a feasible model (Site 5) highlight the challenges that are inherent in conducting a definitive multisite effectiveness trial of HWF. Any implementation at scale will require careful and staged adaptations that test how HWF can benefit communities in the 'post-COVID-19' landscape. This includes matching of population characteristics to the healthcare intervention, and low burden evaluation through routine data collection. However, we anticipate that the HWF model would become more feasible in the post-pandemic context, especially as financial supports ended, and cost-of-living pressures increased.[23]

For peer review only - http://bmjopen.bmj.com/site/about/guidelines.xhtml

The intention of HWF is to use universal healthcare to screen and respond to financial hardship, to prevent financial crisis and its negative impacts on children and families. The sizeable proportion of caregivers disclosing financial hardship in the universal CFH services was commensurate with the low socioeconomic status of the participating sites. Even so, screening for financial hardship challenged many providers' perception of their model of care. The levels of financial hardship did not outweigh other negatives such as stigma, lack of knowledge of financial counselling, the burden of the research processes, or the pandemic disruption. There are parallels between this study and evaluation of the roll-out of family violence screening by CFH services in Victoria, which found that CFH providers selectively screened women about family violence.[24] Nurses avoided screening all women – typically those with lower incomes who are more likely to experience family violence – because of ongoing individual and system practice barriers experienced when identifying and responding to needs.[24] Unsurprisingly, in our pilot, implementing HWF was more feasible for CFH providers already providing higher intensity, outreach support. It was in a site where almost all families were experiencing financial hardship that the HWF model added sufficient value to providers and caregivers for use. In sites where universal CFH services are provided, a tiered care model of those screening to have psychosocial adversity being supported via an integrated child and family program (e.g. a 'one stop shop' hub) incorporating HWF model might increase acceptability and engagement with the program.

5. CONCLUSION

The challenge for the Australian health system is that interventions to address financial hardship traditionally sit "outside" the sector. This results in substantial health system inefficiencies and increased health burdens and costs.[25] Health services alone cannot redress the negative health impacts of financial hardship. The complexity of this challenge can only be meaningfully addressed by integrated, multi-sectoral, and multidisciplinary approaches. As inflation and cost of living increases, we need sustainable and cost-effective responses to financial hardship and its effects on families in the early years. There is an argument for reorienting universal services such as CFH to address the adverse social conditions facing families and driving inequitable outcomes for children and parents.[26,27]

For peer review only - http://bmjopen.bmj.com/site/about/guidelines.xhtml

LIST OF MAIN AND SUPPLEMENTARY FIGURES AND TABLES

MAIN

Figure 1. Healthier Wealthier Families data collection phases and research designs.
Table 1: Methods for the Healthier Wealthier Families implementation and quantitative evaluation**.**
Table 2: Methods for the Healthier Wealthier Families implementation and qualitative evaluation.
Table 3. Feasibility measures by Site.
Table 4: Activities and benefits of financial counselling in Site 5.

SUPPLEMENTARY

Supplementary Material 1: Australia's public health restrictions and economic responses during the COVID-19 pandemic.
Supplementary Figure 1. Participant flow of caregivers (CONSORT).
Supplementary Figure 2. Healthier Wealthier Families key elements, enabling ingredients, and benefits.
Supplementary Table 1: Core and subthemes identified by the implementation evaluation, with descriptions and illustrative quotes.
Supplementary Table 2: Enrolment characteristics of caregivers described by n (Sites 1-4) and n (%) (Site 5) unless specified.
Supplementary Table 3: Secondary outcome measures.

For peer review only

Table 1: Methods for the Healthier Wealthier Families implementation and quantitative evaluation

Site	1	2	3	4	5
**Design & reporting framework**	Pilot RCT (as per published Protocol)[14] CONSORT			Pilot RCT (simplified processes) CONSORT	Pre-post evaluation STROBE
**State (metropolitan or regional)**	Victoria (metropolitan)	Victoria (regional)	New South Wales (metropolitan)	New South Wales (regional)	Victoria (metropolitan)
**Screening period**	3 March to 3 April 2020 (1 month)	26 August 2020 to 23 November 2021 (14 months)	2 September 2020 to 6 April 2021 (4 months)	18 Oct 2021 to 16 May 2022 (7 months)	21 June 2021 to 23 May 2022 (12 months)
**CFH service tier**	Universal	Higher intensity	Universal	Universal	Higher intensity
**CFH providers screening p/week**	1-3	1	2	11	1-2
**Randomisation unit**	Individual			Individual	Not applicable
**Sample size**	180 participants (60 per site) with an anticipated 135 (75%) providing 6-month data. As Teare et al., (2014) report, this sample size is sufficient for informing the critical parameters when designing a definitive RCT (i.e., n=70/120 for continuous/binary outcomes).[28]			The target was a minimum of 60 enrolled caregivers per site (30 per arm).	The target was a minimum of 30 caregivers enrolled and referred to financial counselling.
**Participants**	Caregivers attending the participating CFH services were eligible for the study if they were the caregiver of a young child aged 0-5 years old; lived within the study's geographical and service boundaries; and reported at least one risk factor for financial hardship as screened by the CFH provider (see below). The study-designed screening items were drawn from national and local datasets describing the characteristics most likely to identify families with young children who were at risk of or experiencing poverty and deprivation,[29-31] and co-designed with CFH and financial counselling partners from Site 1 (described in the Protocol)[14]. For Sites 4-5, they were adapted following implementation evaluation and CFH provider preferences.<br>Caregivers were excluded if they (i) could not comprehend the recruitment invitation (e.g. due to substantial cognitive disability); (ii) were already enrolled and assigned a research participant ID; (iii) had no mechanism for contact (telephone or email); (iv) already an active client in a financial counselling service. For the RCT study designs, caregivers identified as high-risk for high impact consequences were not randomised. This group (termed "Priority 1") was referred directly to financial counselling.				
**Financial hardship criteria**	1. In the last year, because of money pressure, caregiver missed or put off: (a) Mortgage or rent repayments, (b) Electricity, gas, water bills, (c) Buying prescription medicines, (d) Paying home or car insurance.<br>2. No one in household has a paid job.<br>3. Family does not have at least $500 in savings for an emergency.			As per Sites 1-3, except Item 3 replaced with:<br>3. Other financial pressure.	As per Sites 1-3, plus the addition of:<br>4. Other financial pressure, plus description.
**Informed consent**	After being sent a welcome email with links to the secure study database, consent could be completed online by the caregiver, or by phone with the researcher.				Conducted with CFH provider following eligibility, recorded on study database.
**Intervention arm**	The purpose of financial counselling (the intervention) is to help clients maximise their income, reduce debt and help with budgeting. The intervention follows usual service delivery, which is tailored to a caregiver's personal and financial needs. For non-English speaking clients, it is usual practice to deliver the service via a multilingual counsellor, or with an independent telephone interpreter. Following allocation to the				

For peer review only

Table 1: Methods for the Healthier Wealthier Families implementation and quantitative evaluation

Site	1	2	3	4	5
	intervention, a referral with the participant's contact information was emailed to the financial counsellor intake address, and a financial counsellor assigned. The financial counsellor contacted the participant to arrange the first appointment, which included confirming contact and demographic details; ascertaining capacity to make financial decisions; discussing and prioritising financial needs; and planning for working together. Caregivers were asked to gather and send paperwork to financial counsellors or external agencies and assisted by the financial counsellor to complete forms in a timely and accurate manner. Financial counsellors often contact and negotiate with creditors on a caregiver's behalf. Each participant had as many appointments with the financial counsellor as they needed, over an unbounded period (unrelated to the study timeline).				
**Control arm**	The comparator received usual care plus money advice that was freely available on the federally-funded Australian Securities and Investments Commission MoneySmart website.				Not applicable.
**Randomisation procedure**	A statistician prepared the randomisation schedule using block randomisation. Participants were randomly assigned to either control or intervention arm with a 1:1 allocation following a computer-generated randomisation schedule stratified by site, using permuted blocks.				Not applicable
**Blinding (masking)**	Participants were asked not to disclose their randomisation status during questionnaires. The research managers and participants were aware of randomisation to enable allocation and intervention delivery. Financial counsellors were informed of intervention participants but not control participants.				Not applicable.
**Primary outcome [quantitative measures]:** ***Feasibility***	Number/proportion of:<br>• Potential participants who agreed to complete screening survey [CFH report based on service records].<br>• Eligible caregivers who consented to participate [CFH and study records].<br>• Participants who accessed, attended, and completed the intervention [financial counsellor report, caregiver report].<br>• Caregivers who completed the follow-up questionnaire [study completion records].				
**Secondary outcomes**	Please see Supplementary Table 3.				
**Outcome of feasibility trial for progressing to large-scale trial**	Definite Go was defined as: (i) ≥50% of eligible caregivers consenting to pilot trial; (ii) ≥60% of those in the intervention arm receiving the intervention; (iii) ≥60% retention of consented caregivers to 6-month follow-up questionnaire.<br>Definite Stop was defined as: (i) <30% of eligible caregivers consenting to pilot trial; (ii) <30% of those in the intervention arm receiving the intervention; (iii) <30% retention of consented caregivers to 6-months follow-up questionnaire.				
**Fidelity measures**	Financial counsellors were invited to complete a questionnaire for each intervention caregiver, designed to match usual data collection. It included issues/goals; frequency of caregiver contact and compliance; activities and advice; and known changes in financial status.				
**Data collection procedures**	Researchers contacted caregivers to invite them to complete baseline and follow-up questionnaires. Financial counsellors were invited to complete a "fidelity" questionnaire for each intervention caregiver who engaged with the service, to measure the activities and advice delivered.				Financial counsellor reported demographic, intake, and follow-up data for the pilot.
**Analysis**	Feasibility, caregiver characteristics and impact measures were described using proportions for categorical data and mean (standard deviation (SD)) for continuous data. Due to the small sample sizes, no statistical analyses (described in the Protocol)[14] were conducted.				

CONSORT: Consolidated Standards of Reporting Trials. STROBE: Strengthening The Reporting of Observational Studies in Epidemiology.

For peer review only - http://bmjopen.bmj.com/site/about/guidelines.xhtml

Table 2: Methods for the Healthier Wealthier Families implementation and qualitative evaluation

**Reporting framework**	Consolidated Criteria for Reporting Qualitative Research (COREQ)[32]
**Purpose**	To understand barriers and enablers to feasibility in Sites 2-5, and the impacts for caregivers in Site 5. Service changes due to the pandemic meant it was not possible to conduct the implementation evaluation in Site 1.
**Research team & reflexivity**	Four researchers (denoted (R)) conducted the implementation evaluation.
Personal characteristics	
Education and occupation	R1: Master of Public Health, project coordinator for the Healthier Wealthier Families project in Victoria.<br>R2: PhD, Postdoctoral researcher at the Centre for Community Child Health, Victoria.<br>R3: PhD, Postdoctoral fellow and project coordinator for the Healthier Wealthier Families project in NSW.<br>R4: Graduate, Research assistant for the Healthier Wealthier Families project in NSW.
Gender	All researchers identified as female.
Experience and training	R1 was trained in qualitative methods and had experience on several projects conducting qualitative interviews.<br>R2 had previously conducted and published peer reviewed qualitative research.<br>R3 had previously conducted qualitative research and was in the process of publishing their research.<br>R4 had previously conducted qualitative research working with families and children.
**Relationship with participants**	
Relationship established, participant knowledge of interviewer	• In Sites 2 & 3, R3 & R4 conducted interviews with the service providers. R3 & R4 had established relationships with the team in Site 3 from regular project meetings but no previous relationship with the provider in Site 2.<br>• In Sites 4 & 5, R2 conducted interviews with the service providers; there were no existing relationships. As R1 had previous interactions they did not conduct these interviews.<br>• In Site 5, R1 & R2 conducted the caregiver interviews; there were no existing relationships.
Interviewer characteristics	• The involvement of R1 & R3 in the conceptualisation and implementation of the project meant there was the potential for bias related to emotional investment.<br>• Reflexive journals were kept by R1 & R3 and used to document reflections after their interviews/focus group.
**Study design**	
Theoretical framework	The methodological orientation was reflexive thematic analysis.
Participant selection	
Sampling	Purposeful, convenience samples of service provider and caregiver participants were invited due to the small number of potential participants. Caregivers were remunerated with gift cards for their time in interviews (AU$40 per hour).
Method of approach	In Sites 2 & 3: The CFH management team and providers were invited to take part in the focus group and interviews by R3 via phone call. The financial counsellor was invited to take part in the interview by R4 via phone call.<br>In Sites 4 & 5: Caregiver participants were asked if they would be interested in participating in an interview at their last appointment with the financial counsellor. Those that had conversational English were approached to be interviewed. Those that did not have conversational English were approached with an interpreter in their first language. In Site 4, email addresses of the service providers who participated in the project were provided by the team leader. R2 emailed to invite participation in an interview. In Site 5, all service providers were invited to participate in an interview via email by R2.<br>In all Sites, participants (caregivers and service providers) were asked to complete a consent form before interview.

For peer review only - http://bmjopen.bmj.com/site/about/guidelines.xhtml

Table 2: Methods for the Healthier Wealthier Families implementation and qualitative evaluation

Sample size	• In Sites 2 & 3: R3 conducted a focus group with 5 CFH providers and 1 interview with a CFH provider.<br>• In Site 3, R4 conducted interviews with CFH providers and 1 financial counsellor.<br>• In Sites 4 & 5: R2 conducted interviews with 6 service providers 1 caregiver.<br>• In Site 5: R1 conducted interviews with 7 caregivers.<br>All service providers and clients identified as female. No other demographic information was collected.
Non-participation	• In Site 3, 5 service providers were invited to participate and none (0) declined.<br>• In Site 4, 10 service providers were invited to participate, 4 did not respond, 1 declined, 1 agreed but was sick on the day of the interview and was not rescheduled.<br>• In Site 5, 10 caregivers were interested in participating; however, two were not interviewed. One was unavailable at their scheduled interview time. One was not engaged due to difficulties in finding an interpreter in their first language.
Setting	
Setting of data collection, and recording	• Data in Sites 2 & 3 were collected by a focus group held face-to-face and individual interviews conducted by phone. These were recorded verbatim, transcribed by an external agency, cleaned, and saved as Word document.<br>• Data in Sites 4 & 5 were collected via phone or videoconferencing. These were electronically transcribed, cleaned, and saved as Word documents.
Presence of non-participants	None.
Description of sample	Site 2 was in regional Victoria, Site 3 was in metropolitan NSW, Site 4 was in regional NSW, Site 5 was in metropolitan Victoria (see Table 1).
Site Data collection	
Interview guide	Interview guides were developed for service provider and caregiver interviews using the Consolidated Framework for Implementation Research (CFIR)[19] and included prompts from the author/researchers. They were not piloted before use.
Repeat interviews	Not conducted.
Field notes	Some field notes were made by some of the research team after interviews and the focus group.
Duration	The focus group was 50 minutes long. Interviews with service providers and caregivers were 20-50 minutes long.
Data saturation	Due to the small number of participants in this pilot study, and who were subsequently interviewed, it is unclear whether data saturation occurred. We note that the concept of data saturation is contested in the literature.[33]
Transcripts returned	Transcripts were not returned to participants for comment.
**Data analysis**	
Number of data coders	The four researchers who conducted interviews and focus group also coded the data, in collaboration with the project lead who did not conduct any interviews or the focus group.
Description of coding tree	Data were systematically categorized into themes and codes during analysis.
Derivation of themes	Themes were derived from the data. R1, R2, R3 and the project lead met several times to discuss themes. Themes were then finalised by R1, R2 and the project lead.
Software	NVivo,[34] and Microsoft Word were used for analysis of data.
Participant checking	No.

For peer review only - http://bmjopen.bmj.com/site/about/guidelines.xhtml

Table 3. Feasibility measures by Site

Caregiver screening, eligibility and follow-up, n (%)	Pilot randomised controlled trial (RCT) Original (as per Protocol)[14]			Simplified	Pre-post evaluation
	Site 1	Site 2	Site 3	Site 4	Site 5
Site characteristics	Metro Vic Universal	Regional Vic Higher intensity	Metro NSW Universal	Regional NSW Universal	Metro Vic Higher intensity
Potential sample size	n/a[a]	107	117	411	69
Number of caregivers approached by CFH providers	61 (74.6)[a]	107 (100)	117 (100)	80 (19.5)	69 (100)
Of caregivers approached, n (%) who agreed to screening	47 (77.1)	107 (100)	70 (59.8)	64 (80.0)	67 (97.1)
Of caregivers approached, n (%) requested/preferred interpreter	0	0	10 (8.6)	0	15 (21.7)
Of caregivers screened, n (%) eligible (financial hardship)	17 (36.2)	20 (18.7)	29 (41.4)	13 (20.3)	66 (98.5)
Of eligible caregivers, n (%) interested in enrolling	14 (82.4)	9 (45.0)	26 (89.7)	9 (69.2)	64 (97.0)
Of eligible caregivers, n (%) who enrolled	1 (5.9)	4 (20.0)[b]	6 (20.7)[b]	2 (15.4)	49 (77)[c]
Of enrolled caregivers, n (%) who provided follow-up data	1	4	0	0	44 (90)[c]
Breakdown of financial hardship items, n (%) who agreed to screening					
1. Missed or put off:					
(a) Mortgage or rent repayments	6 (12.8)	0	15 (21.4)	2 (3.1)	26 (39.4)
(b) Electricity, gas, water bills	13 (27.7)	8 (7.5)	15 (21.4)	9 (14.1)	46 (68.7)
(c) Buying prescription medicines	5 (11.4)	5 (4.7)	12 (17.1)	1 (1.6)	20 (29.9)
(d) Paying home or car insurance	9 (20.0)	2 (1.9)	11 (15.7)	2 (3.1)	19 (28.4)
2. No one in household has a paid job	0	20 (18.7)	18 (25.7)	5 (7.8)	50 (74.6)
3. Does not have $500 in savings	8 (17.0)	10 (9.4)	14 (20.0)	Not collected	63 (95.5)
4. Other financial pressure	Not collected	Not collected	Not collected	4 (6.3)[d]	43 (81.1)[d]

CFH: Child and Family Health. Metro: metropolitan. NSW: New South Wales. Vic: Victoria.

[a] Data on potential caregivers provided for 3 of the 5 weeks of screening (44 screened of 59 approached).

[b] Enrolments included one caregiver in Site 2 and two caregivers in Site 3 who were immediately referred for priority indicators (not randomly allocated, see Methods in Table 1).

[c] Reported by the financial counsellor instead of caregivers.

[d] Asking about other financial pressures commenced in mid-July 2021, after 16 caregivers had been screened in Site 4 (i.e. denominator is n=64) and 14 caregivers had been screened in Site 5 (denominator is n=53). Other financial pressures included: affording essential items (predominately food, n=16); general financial hardship (e.g. low income, business strain, n=13); fines and debt (e.g. car fines, childcare and Centrelink debt, n=12); unpaid bills (e.g. medical, utilities, n=3); and not specified (n=3).

For peer review only - http://bmjopen.bmj.com/site/about/guidelines.xhtml

Table 4: Activities and benefits of financial counselling in Site 5

Actions and benefits recorded in routine data	No	Not applicable	Yes	If Yes, amount range where specified
Completion of an income & expenditure statement	29 (65.9)	12 (27.3)	3 (6.8)	p/fortnight: deficits of $103, $200, $240
Check caregiver is receiving full entitlements	6 (13.6)	0	38 (86.4)	p/fortnight: $0 to $1400
Accessed all concessions that caregiver is eligible for	3 (6.8)	13 (29.6)	28 (63.6)	$0 to $2200
Is caregiver aware of Centrepay	8 (18.2)	18 (40.9)	18 (40.9)	(n/a)
Negotiated moratorium	5 (11.4)	37 (84.1)	2 (4.6)	2 credit cards totalling $16,000, one unspecified
Established/negotiated payment plan/s with creditor/s	10 (22.7)	30 (68.2)	4 (9.1)	p/fortnight: $10, $180, $200
Negotiated debt reduction or waiver/s	3 (6.8)	38 (86.4)	3 (6.8)	$300, $500, $12000
Considered or accessed grant/s	14 (31.8)	15 (34.1)	15 (34.1)	$0 to $1330
Considered or accessed no interest loan	24 (54.6)	10 (22.7)	10 (22.7)	$0 to $5000
Accessed brokerage from with agency	26 (60.5)	4 (9.3)	13 (30.2)	$100 to $265
Considered or accessed superannuation	17 (38.6)	25 (56.8)	2 (5.6)	$0, $3000
Organised external referrals [a]	21 (47.7)	5 (11.4)	18 (40.9)	(n/a)
Conducted financial literacy activities [b]	10 (22.7)	5 (11.4)	29 (65.9)	(n/a)
**Achieved**				
Avoidance of bankruptcy	0	44 (100)	0	
Avoidance or curtailment of legal action	0	39 (88.6)	5 (11.4)	
Stabilised caregiver's housing situation	6 (14.0)	31 (72.1)	6 (14.0)	
Avoided loss of utilities	0	21 (47.7)	23 (52.3)	
Avoided loss of mobile phone and internet access	0	42 (97.7)	1 (2.3)	
Anything else the caregiver wanted to change [c]	10 (22.7)	1 (2.3)	33 (75.0)	

Centrelink is a Government service which provides support to Australians who face financial hardship. Centrepay is a free bill paying service that makes regular deductions from Centrelink payments. Fortnight: biweekly (14 days). (n/a): not applicable.

[a] Top 3: housing/rent assistance (n=7); employment assistance (n=6); emergency relief services (n=3).

[b] Top 3: financial wellbeing information (n=19, noting n=8 in language other than English); government MoneySmart website (n=18); budgeting with template (n=7).

[c] Top 3: Centrelink entitlements (n=11); material aid and emergency relief (both n=7).

For peer review only

ETHICAL APPROVAL

This research was approved by the Human Research Ethics Committees of The Royal Children's Hospital (HREC/57372/RCHM-2019; Sites 1, 2, 4-5), and South West Sydney Local Health District (2019/ETH13455; Site 3).

CONTRIBUTORSHIP STATEMENT

AP Conceptualization, Methodology, Validation, Formal analysis, Investigation, Resources, Data Curation, Writing - Original Draft Preparation, Writing - Review & Editing, Visualization, Supervision, Project administration, Funding acquisition
NW Conceptualization, Methodology, Software, Validation, Formal analysis, Investigation, Data Curation, Writing - Review & Editing, Supervision, Project administration
JB Methodology, Validation, Formal analysis, Investigation, Resources, Data Curation, Writing - Review & Editing, Project administration, Funding acquisition
AZ Conceptualization, Methodology, Writing - Review & Editing, Funding acquisition
DCS Conceptualization, Methodology, Writing - Review & Editing
SW Investigation, Writing - Review & Editing, Project administration
MS Methodology, Validation, Investigation, Resources, Data Curation, Writing - Review & Editing, Project administration
KT Methodology, Validation, Investigation, Resources, Data Curation, Writing - Review & Editing, Project administration
MM Methodology, Validation, Investigation, Resources, Writing - Review & Editing, Project administration
JC Methodology, Validation, Investigation, Resources, Data Curation, Writing - Review & Editing, Project administration
RB Methodology, Investigation, Resources, Data Curation, Writing - Review & Editing, Project administration
SC Methodology, Validation, Investigation, Resources, Data Curation, Writing - Review & Editing, Project administration
LB Methodology, Validation, Investigation, Resources, Data Curation, Writing - Review & Editing, Project administration
DS Methodology, Validation, Investigation, Resources, Data Curation, Writing - Review & Editing, Project administration
RR Methodology, Validation, Investigation, Resources, Data Curation, Writing - Review & Editing, Project administration
AW Methodology, Validation, Formal analysis, Investigation, Data Curation, Writing - Review & Editing
NS Validation, Formal analysis, Investigation, Writing - Review & Editing, Project administration
RG Resources, Writing - Review & Editing
SR Methodology, Resources, Writing - Review & Editing
LK Methodology, Writing - Review & Editing
RL Methodology, Resources, Writing - Review & Editing, Funding Acquisition
VE Resources, Writing - Review & Editing, Funding Acquisition
SW Conceptualization, Methodology, Resources, Writing - Review & Editing, Supervision, Funding acquisition
SG Conceptualization, Methodology, Resources, Writing - Review & Editing, Supervision, Funding acquisition

All authors made substantial contributions to the conception or design of the paper; drafted this paper or revised it critically for important intellectual content; approved the final version submitted; and agree to be accountable for all aspects of the research.

For peer review only - http://bmjopen.bmj.com/site/about/guidelines.xhtml

DATA SHARING STATEMENT

Data are available upon reasonable request to hwf.study@mcri.edu.au (subject to ethical and legal approvals).

COMPETING INTERESTS STATEMENT

None declared.

FUNDING STATEMENT

The Healthier Wealthier Families (HWF) pilot was supported by funding from the Helen Macpherson Smith Trust (Impact Grant #9523), The Corella Fund, the Murdoch Children's Research Institute (MCRI), Health@Business and University of New South Wales (UNSW) Medicine Collaboration Seed Funds Grant, the Population Child Health Group at the UNSW, Sydney Partnership for Health, Education, Research and Enterprise (SPHERE), and the Best-START SW at the Ingham Institute. The MCRI administered the philanthropic grants and provided infrastructural support (as study sponsor) to its staff but played no role in the conduct or analysis of the trial. Research at the MCRI is supported by the Victorian Government's Operational Infrastructure Support Program. Author AP was supported by The Erdi Foundation Child Health Equity (COVID-19) Scholarship; Author SW was supported by a National Health and Medical Research Council (NHMRC) Career Development Fellowship (#1158954); Author SG was supported by a NHMRC Practitioner Fellowship (#1155290).

ACKNOWLEDGEMENTS

We thank all families, children, Child and Family Health providers, and financial wellbeing providers who collaborated on Healthier Wealthier Families (HWF). We thank members of the Australian and International HWF Advisory Groups for their oversight and guidance, especially the team at Glasgow Centre for Population Health. We thank contributors who donated time in-kind over and above the project resources which made the pilot possible, and to the funders for their flexibility around project deliverables during the COVID-19 pandemic. The HWF pilot collaboration is led by the Centre for Community Child Health and BEST START-SW, in partnership with Maternal and Child Health Services, Child and Family Health Services, Uniting Vic.Tas, Upper Murray Family Care, Wesley Mission, the Melbourne Institute: Applied Economic & Social Research, the University of Melbourne, RMIT University, Western Sydney University, and the University of New South Wales.

REFERENCES

1. Cooper K, Stewart K. Does Household Income Affect children's Outcomes? A Systematic Review of the Evidence. Child Indicators Research. 2020;14:981-1005.
2. Shonkoff JP, Garner AS, Committee on Psychosocial Aspects of Child and Family Health, Committee on Early Childhood, Adoption, and Dependent Care, and Section on Developmental and Behavioral Pediatrics, Siegel BS, Dobbins MI, Earls MF, Garner AS, McGuinn L, Pascoe J, Wood DL. The lifelong effects of early childhood adversity and toxic stress. Pediatrics. 2012 Jan;129(1):e232-46.
3. Price AMH, Measey M-A, Hoq M, Rhodes A, Goldfeld S. Child and caregiver mental health during 12 months of the COVID-19 pandemic in Australia: findings from national repeated cross-sectional surveys. BMJ Paediatrics Open. 2022;6(1):e001390.
4. Poverty in Australia 2022: A snapshot is published by the Australian Council of Social Service, in partnership with UNSW Sydney.

For peer review only - http://bmjopen.bmj.com/site/about/guidelines.xhtml

5. Australian Early Development Census. An Australian Government Initiative. https://www.aedc.gov.au/.
6. Brinkman S, Gregory T, Harris J, Hart B, Blackmore S, Janus M. Associations between the early development instrument at age 5, and reading and numeracy skills at ages 8, 10 and 12: a prospective linked data study. Child Indicators Research. 2013;6(4):695-708.
7. Goldfeld S, O'Connor M, Chong S, Gray S, O'Connor E, Woolfenden S, et al. The impact of multidimensional disadvantage over childhood on developmental outcomes in Australia. International Journal of Epidemiology. 2018;47(5):1485-96.
8. The Health Foundation (2017). Healthy lives for people in the UK. Strategic plan. https://www.health.org.uk/publications/healthy-lives-for-people-in-the-uk.
9. Garg A, Cull W, Olson L, Boyd AF, Federico SG, Dreyer B, et al. Screening and Referral for Low-Income Families' Social Determinants of Health by US Pediatricians. Acad Pediatr. 2019;19(8):875-83.
10. Loveday S, Hall T, Constable L, Paton K, Sanci L, Goldfeld S, et al. Screening for Adverse Childhood Experiences in Children: A Systematic Review. Pediatrics. 2022;149(2).
11. Hernanz V, Malherbet F, Pellizzari M. Take-Up of Welfare Benefits in OECD Countries: A Review of the Evidence. Economics. 2004;https://doi.org/10.1787/525815265414.
12. Scutella R, Johnson DT. Understanding and Improving Data Quality Relating to Low-Income Households. FaHCSIA Policy Research Paper. 2005(24).
13. Wiggan J, Talbot C. The benefits of welfare rights advice: a review of the literature. National Association of Welfare Rights Advisors. 2006.
14. Price AMH, Zhu A, Nguyen HNJ, Contreras-Suárez D, Schreurs N, Burley J, et al. Study protocol for the Healthier Wealthier Families (HWF) pilot randomised controlled trial: testing the feasibility of delivering financial counselling to families with young children who are identified as experiencing financial hardship by community-based nurses. BMJ Open. 2021;11(5):e044488.
15. Burley J, Samir N, Price A, Parker A, Zhu A, Eapen V, et al. Connecting Healthcare with Income Maximisation Services: A Systematic Review on the Health, Wellbeing and Financial Impacts for Families with Young Children. Int J Environ Res Public Health. 2022;19(11).
16. Naven L, Egan J. Healthier, Wealthier Children: Learning from an Early Intervention Child Poverty Project; GCPH: Glasgow, UK. 2013.
17. Naven L, Withington R, Egan J. Maximising opportunities: final evaluation report of the Healthier, Wealthier Children (HWC) project. Glasgow: Glasgow Centre for Population Health.; 2012.
18. NHS Greater Glasgow and Clyde. Healthier Wealthier Children. https://www.nhsggc.scot/your-health/public-health/maternal-and-child-public-health/healthier-wealthier-children/.
19. The Consolidated Framework for Implementation Research (CFIR). Ann Arbor: CFIR, 2019. Available from the CFIR website https://cfirguide.org/constructs/.
20. Honisett S, Loftus H, Liu H, Montgomery A, De Souza D, Hall T, et al. Do Australian policies enable a primary health care system to identify family adversity and subsequently support these families—A scoping study. Health Promotion Journal of Australia. 2023;34(1):211-21.
21. Chambers DA, Glasgow RE, Stange KC. The dynamic sustainability framework: addressing the paradox of sustainment amid ongoing change. Implement Sci. 2013;8:117.
22. Bonevski B, Randell M, Paul C, Chapman K, Twyman L, Bryant J, et al. Reaching the hard-to-reach: a systematic review of strategies for improving health and medical research with socially disadvantaged groups. BMC medical research methodology. 2014;14(1):1-29.
23. J.P.Morgan Research Insights. Inflation and the Cost of Living: Are Consumers Spending Less? 14 September 2022 https://www.jpmorgan.com/insights/research/inflation-cost-of-living.
24. Hooker L, Taft A. Who is Being Screened for Intimate Partner Violence in Primary Care Settings? Secondary Data Analysis of a Cluster Randomised Trial. Maternal and Child Health Journal. 2021;25(10):1554-61.
25. Teager W, Fox S, Stafford N. How Australia can invest early and return more: A new look at the $15b cost and opportunity. Early Intervention Foundation, The Front Project and CoLab at the Telethon Kids Institute, Australia.; 2019.

For peer review only - http://bmjopen.bmj.com/site/about/guidelines.xhtml

26. Singh G, Zhu H, Cheung CR. Public health for paediatricians: Fifteen-minute consultation on addressing child poverty in clinical practice. Archives of disease in childhood - Education & practice edition. 2021;106(6):326-32.
27. Axford N, Berry V. Money Matters: Time for Prevention and Early Intervention to Address Family Economic Circumstances. Journal of Prevention. 2023.
28. Teare MD, Dimairo M, Shephard N, Hayman A, Whitehead A, Walters SJ. Sample size requirements to estimate key design parameters from external pilot randomised controlled trials: a simulation study. Trials. 2014;15(1):264.
29. Melbourne Institute: Applied Economics & Social Research. The Household, Income and Labour Dynamics In Australia Survey: Selected Findings from Waves 1 to 17. Report. Melbourne, Australia: The University of Melbourne, Melbourne Institute; 2019.
30. Australian Institute of Family Studies. (2018). Longitudinal Study of Australian Children Data User Guide – December 2018. Melbourne: Australian Institute of Family Studies. Accessed from https://growingupinaustralia.gov.au/data-and-documentation/data-user-guide.
31. Saunders P, Zhu A. Comparing Disadvantage and Well-being in Australian Families [online]. Australian Journal of Labour Economics. 2009;12(No. 1):21-39.
32. Tong A, Sainsbury P, Craig J. Consolidated criteria for reporting qualitative research (COREQ): a 32-item checklist for interviews and focus groups. International Journal for Quality in Health Care. 2007;19(6):349-57.
33. Braun V, Clarke V. To saturate or not to saturate? Questioning data saturation as a useful concept for thematic analysis and sample-size rationales. Qualitative Research in Sport, Exercise and Health. 2021;13(2):201-16.
34. QSR International Pty Ltd. (2018). NVivo (Version 12). [Computer Software]. https://www.qsrinternational.com/nvivo-qualitative-data-analysis-software/home [Internet].

For peer review only

For peer review only - http://bmjopen.bmj.com/site/about/guidelines.xhtml

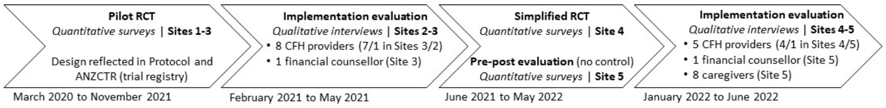

Figure 1. Healthier Wealthier Families data collection phases and research designs.

338x190mm (96 x 96 DPI)

LIST OF SUPPLEMENTARY CONTENT

Supplementary Material 1: Australia's public health restrictions and economic responses during the COVID-19 pandemic.
Supplementary Figure 1. Participant flow of caregivers (CONSORT).
Supplementary Figure 2. Healthier Wealthier Families key elements, enabling ingredients, and benefits.
Supplementary Table 1: Core and subthemes identified by the implementation evaluation, with descriptions and illustrative quotes.
Supplementary Table 2: Enrolment characteristics of caregivers described by n (Sites 1-4) and n (%) (Site 5) unless specified.
Supplementary Table 3: Secondary outcome measures.
Supplementary References

For peer review only

Supplementary Material 1: Australia's public health restrictions and economic responses during the COVID-19 pandemic

Australia's public health restrictions during the COVID-19 pandemic 2020-21: In Australia, stay-at-home orders ('lockdown') were the main measure of disease control until late 2021 when double-vaccination levels reached 70%-80%. Victoria and NSW were the states with the highest infection rates and the longest and most severe lockdowns. Initially, all Australian states and territories experienced a national, 10-week lockdown from 23 March to 1 June 2020, which disrupted the health and social service systems and required radical change from in-person to virtual service delivery within weeks. Many healthcare workforces, including CFH, were seconded to responsibilities such as COVID-19 testing clinics and, from early 2021, vaccination efforts. Victoria experienced a second, extended, and more severe, 20-week lockdown from 8 July-23 November 2020. There were several short (less than a week) lockdowns in the intervening months, before NSW experienced its second, extended period of lockdown, which began incrementally on 26 June 2021. Victoria experienced its fifth lockdown from 16-27 July 2021, before another extended lockdown (the sixth in total) began a week later on 5 August 2021. Lockdowns in both states ended in late October 2021.

Australia's economic response to the COVID-19 pandemic: To protect against the economic fallout of lockdown (including high un- and under-employment rates), the Australian federal government rapidly implemented a suite of short-term financial supports that became available by April 2020.[1,2] The supports included an unemployment supplement ('JobSeeker') which doubled recipients' social welfare benefits from $550 to $1100 a fortnight;[1] a wage supplement for eligible businesses to retain their workforce ('JobKeeper');[2] allowing early access to superannuation;[3] and free childcare for working families.[4] Banks and creditors also allowed loan repayments to be deferred for up to 10 months. These social policy changes represented some of the largest (albeit temporary) in Australia's history. Notably, the JobKeeper and JobSeeker supplements were so significant that, by September 2020, levels of poverty and housing stress in Australia were substantially lower than the levels directly preceding COVID-19.[5] However, by September 2021, as the financial supports were reduced and withdrawn, the proportions of Australians on the lowest income support payments (which are below the poverty line) were 25% higher than before the pandemic.[5]

Supplementary Figure 1: Participant flow of caregivers (CONSORT).

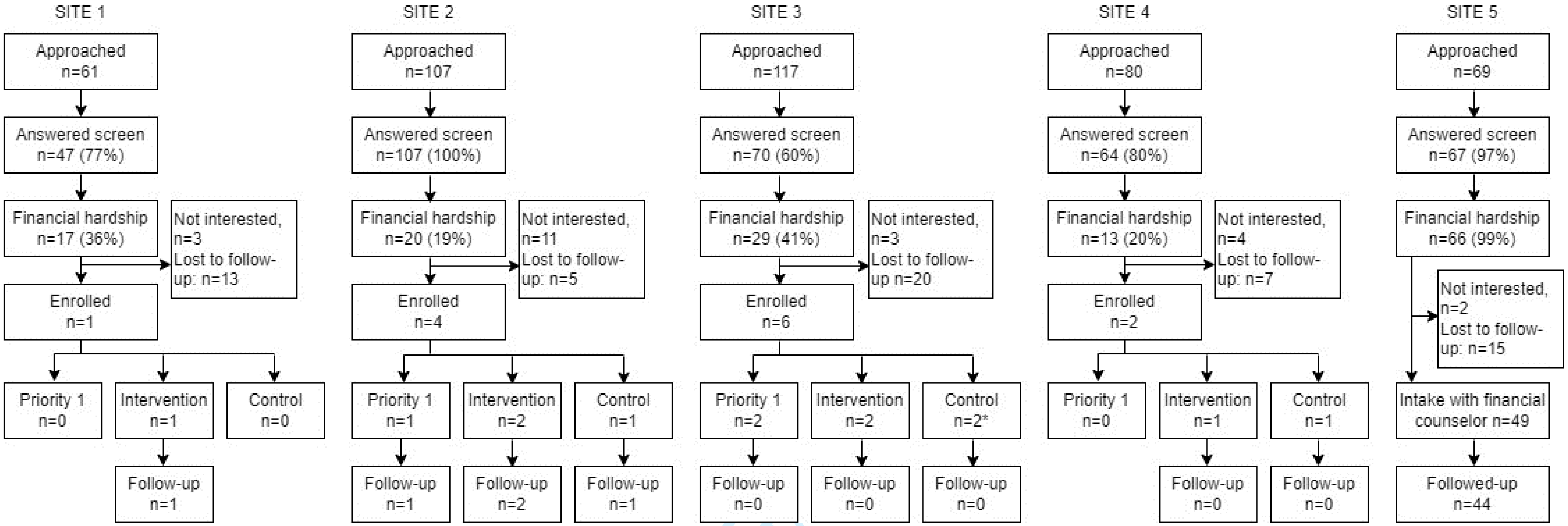

* In Site 3, the n=2 caregivers allocated to the Control arm were subsequently identified as high priority and referred to financial counselling.

For peer review only - http://bmjopen.bmj.com/site/about/guidelines.xhtml

Supplementary Figure 2: Healthier Wealthier Families key elements, enabling ingredients, and benefits.

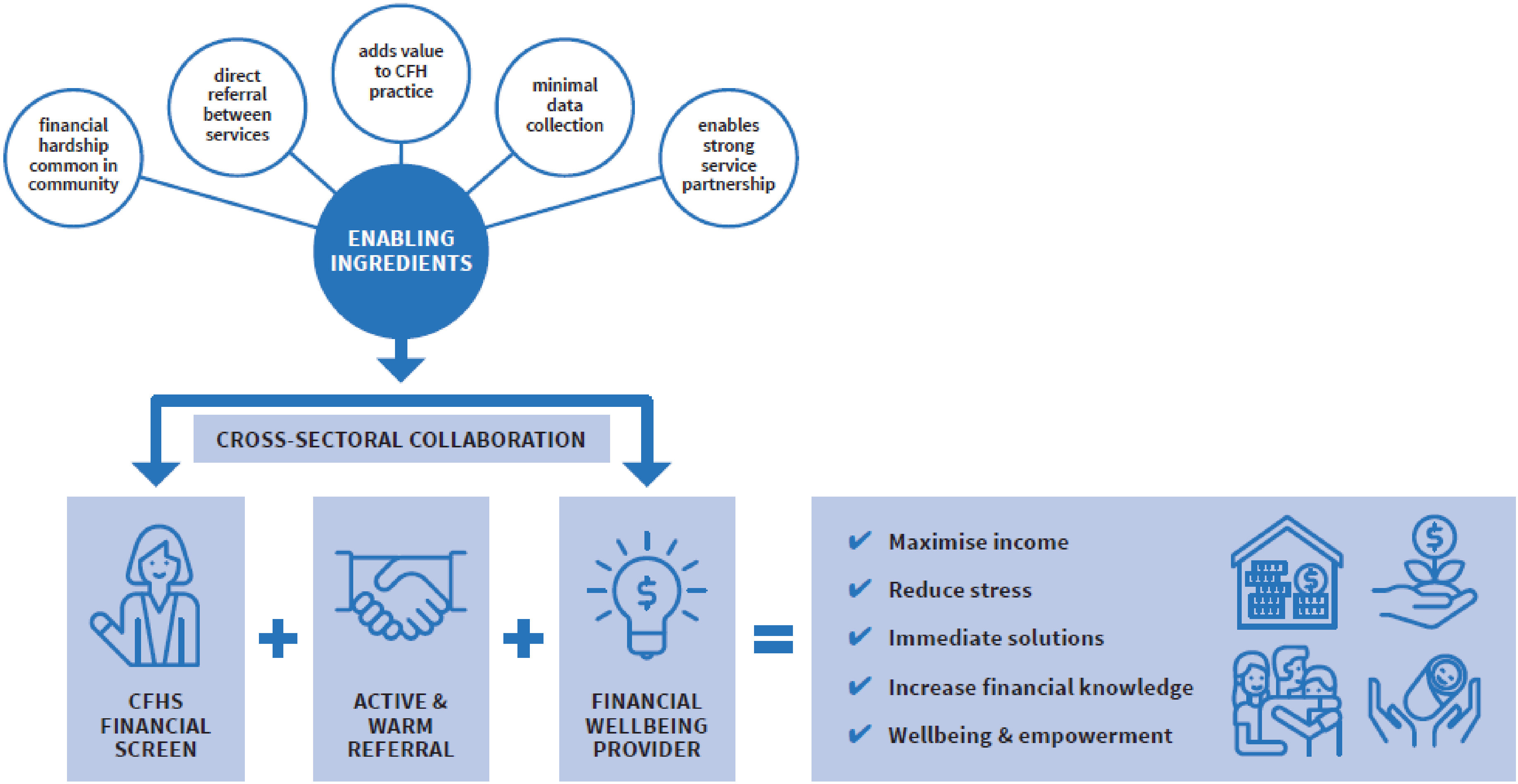

Supplementary Table 1: Core and sub themes identified by the implementation evaluation, with descriptions and illustrative quotes.

**Core and sub themes**	**Description of subthemes with illustrative quotes**
Theme 1. Experiences and perceptions of screening and referral processes	
1.1 Child and Family Health (CFH) providers had differing opinions about the usefulness of financial screening and relevance to their own practice, which were related to delivery of universal or higher intensity services.	Some CFH providers delivering universal care did not have time to use an additional screening tool in practice.<br>*"We only get 45 minutes to see the client…then whatever research project is at the end, and it's not wanting to do it, but it's the added extra work that we have to do on top of everything else that's crammed into that little small time frame…… And obviously these projects just go out the door for us, it's just not a priority."* [CFH PROVIDER, FOCUS GROUP PARTICIPANT, SITE 3]<br><br>Some CFH providers delivering universal services thought the screening tool was out of scope of practice and made it uncomfortable for them to use and made their clients feel uncomfortable.<br>*"…it's not the question that we as child family health bring up, like how are you financially, the financial word does not pop up during our screening to be honest…I find that a little bit uncomfortable to bring up during our clinics, which is very limited because… we have the normal check and the zillion questionaries just for mum and baby."* [CFH PROVIDER, FOCUS GROUP PARTICIPANT, SITE 3]<br><br>Some CFH providers of universal services viewed financial screening as part of providing holistic care.<br>"*…but it is our business if that's actually causing distress for the more, you know, impacting on their ability to parent their children. So I guess that comes back to the education part again, you know, about linking, you know, the fact that financial disadvantage is at the base of a lot of concerns that families may have, although it may not be the initial identifier."* [CFH PROVIDER, PARTICIPANT 21, SITE 3]<br><br>CFH providers offering higher intensity services viewed the identification and response to financial hardship as usual practice.<br>*"….. financial stress for a lot of my families is the biggest component of their day-to-day psychological health. I mean, other things are barriers definitely, but when it's around the financial counselling, it's definitely one of the biggest concerns. Just how we gonna get food into my child's mouth next week……You know, some of these fines people have, are in the thousands of dollars and if they can be reduced because of support of a financial counsellor … you know, it's basic necessities … food is back into the mouths of the children, and they are able to pay another bill. And so the mental health [difficulties] decreases, and their capacity rises." [CFH PROVIDER, PARTICIPANT 11, SITE 5].*
1.2. There were issues with engagement due to the research processes.	The research processes [RCT designs] were burdensome.<br>*"I guess the motivation, again, for doing it that way was that the researcher had more information to share with the clients to then go through and see…if they did meet the criteria. But again, knowing the community,…it was difficult to engage with some people because they don't answer their telephone." [CFH PROVIDER, PARTICIPANT 21, SITE 3]*<br><br>Eligibility screening added complexity for clients.<br>"*the family thought they would get some incentive some money support and would say, oh, yes, I would. And you go through all that and say, oh no you're not eligible and all this and thank you very much. It is like some of them they can come back what was the point of that." [CFH PROVIDER, FOCUS GROUP PARTICIPANT, SITE 3]*
1.3 The COVID-19 pandemic affected service provision and levels of financial hardship.	Financial hardship was reduced for many families due to the government supplements in 2020.<br>*"I found particularly in my area…that a lot of people have been very cashed up, which I wouldn't normally see…Well there's been a lot of extra payments - so there was the COVID payment. And now there's a lot more services coming along who have*

For peer review only - http://bmjopen.bmj.com/site/about/guidelines.xhtml

Core and sub themes	Description of subthemes with illustrative quotes
	a lot of money, are actually using that money to work with families which I hadn't seen before." [CFH PROVIDER, PARTICIPANT 19, SITE 2]<br><br>There were additional pressures on CFH Providers during the COVID-19 pandemic, which exacerbated the challenges of screening.<br>*"…being short staffed and feeling under pressure. A new thing will often get forgotten and left…It was a timing thing. For us, it wasn't great…Doing phone consults during COVID-19 as well, which was new to them and took up a lot of Nurses bandwidth. Using new things then was limited." [CFH PROVIDER, PARTICIPANT 17, SITE 4]*
Theme 2. Barriers to identifying financial hardship and accepting help	
2.1 Stigma surrounding financial hardship and accepting a referral to a Financial counsellor	Service providers identified a myriad of barriers surrounding why clients may not disclose financial hardship or accept support. The most frequently identified barrier was stigma regarding disclosure of financial hardship and a fear of being judged.<br>*"It's a big shame for clients to come forward and mention that they are not able to be in charge of their money, it's a huge stigma. So they won't be very open, there will be a lot of resistance." [CFH PROVIDER, PARTICIPANT 23, SITE 3]*<br><br>Service providers noted that building trust in new services or accepting a new referral can take time with clients. Referrals may not be readily received, even though significant need is identified.<br>*"…that building up of that rapport and that gradual. Some people you need to go gently gently with and – they may not accept it then and there but further down the track they may be more receptive to it". [SERVICE PROVIDER, PARTICIPANT 15, SITE 4]*<br><br>Providers delivering higher intensity (outreach) services noted that it may be more comfortable for clients to have conversations about financial difficulties at home.<br>*"I think there's a bit of stigma. I don't know, I find it works if you actually go out and see people. Yeah, yeah, yeah, they're more comfortable, they're probably going to tell you a little bit more, as well." [CFH PROVIDER, PARTICIPANT 19, SITE 2]*
2.2 Financial counselling was not well understood by CFH providers or families.	CFH providers noted their workforce's levels of understanding; not understanding could lead some providers to de-prioritise financial screening.<br>*"I don't think the CFHN had a full understand[ing]." [CFH PROVIDER, PARTICIPANT 22, SITE 3]*<br><br>Some service providers noted that clients lacked understanding about financial counselling, and the importance of building community awareness.<br>*"… I just don't think people have enough awareness of it to realize what a difference it can make. So I think just lack of knowledge is a barrier." [CFH PROVIDER, PARTICIPANT 17, SITE 4]*<br><br>Knowledge is important for CFH providers to be able to explain financial counselling to help clients engage with services.<br>*"It takes a very very very long time to be able to capture the audience and to get the audience to understand what kind of support it is that you want to give them. And there's a lot of confusion between financial counselling and financial advising and financial planners." [CFH PROVIDER, PARTICIPANT 23, SITE 3]*<br><br>Lack of understanding of how financial counselling can provide support is a significant barrier to accepting a referral. Without knowledge, clients may not have recognised that they could benefit from the service.<br>*"Unless it's money in my pocket, it's not help to me'. Can I access money out of this, or is it just … are they just looking at ways for me to try and not spend money?" [CFH PROVIDER, PARTICIPANT 15, SITE 4]*

For peer review only

Core and sub themes	Description of subthemes with illustrative quotes
2.3. Clients in crisis may have other priorities.	CFH providers noted that it can be difficult for clients in crisis to engage with services; however, when people do disclose, they usually need help immediately. Identifying need before crisis point (early intervention) can be highly beneficial as it is difficult for clients to engage in complex tasks like prioritising needs when under significant stress.<br>*"And the ones that are really in crisis often are so in crisis,… they can barely know what they're going to have for dinner, let alone plan." CFH PROVIDER, PARTICIPANT 17, SITE 4]*
2.4 Financial literacy and cultural/family considerations.	Some service providers suggested that it is important to understand the culture of financial literacy within a community to understand how to engage a family about financial hardship.<br>*"I'm mindful in this area, people are often coming from different cultural backgrounds where, you wouldn't openly talk about your finances. There may be a certain apprehension or fear … and sometimes just engaging with us initially without even going into finances." [CFH PROVIDER, PARTICIPANT 21, SITE 3]*<br>For some families, gender roles were relevant in discussions about finances.<br>*"…for some mothers … they have said before "I don't deal with the finances - that's my husband". So, they don't actually have any financial control." [CFH PROVIDER, PARTICIPANT 11, SITE 5]*
Theme 3. Factors that enabled engagement with families.	It is important that service providers are supported to understand the role of financial counselling, to build trust in a referral, to be able to explain the service to families, and support families to engage with financial counselling.<br>*"So I guess it's around how we make the link for families around that if they're in a non-judgemental, non-stigmatising way, that is that if there is some issues and we could help with some extra supports around finances, if we feel that that's actually impacting upon their ability to cope with life and cope with their baby, then, and we can help them make the link that I think they are more likely to do something about it. I just don't think that they're getting the value on the what's in it for them." [CFH PROVIDER, PARTICIPANT 20, SITE 3]*<br>Provide resources to families about financial hardship and financial counselling.<br>*"…most of our parents do like to do a bit of research on their own so anything that is in written form - whether it be an email or website or whatever - I think that would be helpful. This helps to empower them." [CFH PROVIDER, PARTICIPANT 18, SITE 4]*<br>Ensure accessible communication that is written in plain English and inclusive to all members of the community.<br>*"The community needs educating. Making it more open and more spoken about. we've got some resources too that fairly easily accessible, that people can go to and look at. And that's what I think with the financial struggles as well … that you need more community awareness. We need more .. like your Beyond Blue websites, like your PANDA websites." [CFH PROVIDER, PARTICIPANT 15, SITE 4]*<br>It is important that financial counselling services provide client-centred care, not service led. This was noted for methods of communication, e.g. communicating by text rather than email and doing phone appointments rather than in-person.<br>*"Doing phone appointments works for them." [FC PROVIDER, PARTICIPANT 9, SITE 5]*

For peer review only - http://bmjopen.bmj.com/site/about/guidelines.xhtml

Supplementary Table 2: Enrolment characteristics of caregivers described by n (Sites 1-4) and n (%) (Site 5) unless specified.

Caregiver characteristics	RCT allocation (Sites 1-4)			Pre-post evaluation (Site 5)	
	Priority 1	Intervention	Control	Engaged	Lost
	N=3	N=6	N=4	n=44	n=5
Age, in years					
Mean [range]	40.7 [34-46]	28.6 [23-39]	29.5 [21-42]	Age not collected	
Missing	0	1	0		
Main caregiver to children					
Yes	1	5	4	41 (93.2)	5 (100.0)
No	0	0	0	3 (6.8)	0
Missing	2	1	-	-	-
Number of children					
Mean [range]	3 [3-3]	1.4 [1-2]	1.7 [1-3]	2.2 [1-7]	2.4 [1-3]
Missing	2	1	1	-	-
Partnered					
Yes	0	3	3	26 (59.1)	4 (80.0)
No	1	2	1	2 (4.6)	1 (20.0)
Missing	2	1	-	16 (36.4)	-
Household member with disability or special health care needs					
Yes	1	0	1	21 (47.7)	2 (40.0)
No	0	5	3	23 (52.3)	3 (60.0)
Missing	2	1	-	-	-
Identifies as Aboriginal and/or Torres Strait Islander					
Yes	1	0	1	0	1 (20.0)
No	0	5	3	44 (100.0)	4 (80.0)
Missing	2	1	-	-	-
Born in Australia					
Yes	1	5	2	10 (22.7)	1 (20.0)
No	0	0	2	33 (75.0)	4 (80.0)
Missing	2	1	0	1 (2.3)	-
Speaks English at home					
Yes	2	5	3	20 (45.5)	1 (20.0)
No	0	0	1	24 (54.6)	4 (80.0)
Missing	1	1	-	-	-
Education status					
Primary school (Grade 6) or less	1	1	0	10 (22.7)	2 (40.0)
High school (Grade 12) or less	0	1	4	21 (47.7)	2 (40.0)
Diploma	1	2	0	5 (11.4)	0
Bachelor	0	1	0	8 (18.2)	1 (20.0)
Missing	1	1	-	-	-
In paid work					
Yes	0	0	2	1 (2.3)	0
Parental leave	0	1	2	2 (4.6)	0
No	3	4	0	41 (93.2)	5 (100.0)
Missing	-	1	-	-	-
Looked for work in last four weeks					
Yes	0	0	0	5 (11.4)	1 (20.0)
No	3	5	4	39 (88.6)	4 (80.0)
Missing	-	1	-	-	-
Would like more work					

For peer review only - http://bmjopen.bmj.com/site/about/guidelines.xhtml

Caregiver characteristics	RCT allocation (Sites 1-4)			Pre-post evaluation (Site 5)	
	Priority 1	Intervention	Control	Engaged	Lost
	N=3	N=6	N=4	n=44	n=5
Yes	2	0	1	13 (29.6)	1 (20.0)
No	-	4	2	24 (54.6)	4 (80.0)
Other	0	0	0	6 (13.6)[a]	0
Missing	1	2	1	1 (2.3)	-
Household income less than $50,000 p/annum					
Yes	2	2	2	36 (81.8)	3 (60.0)
No	1	1	0	7 (15.9)	2 (40.0)
Don't know	0	2	2	0	0
Missing	-	1	-	1 (2.3)	-

[a] Of six caregivers who responded 'other': n=2 were studying with the intention to gain employment and n=1 was planning to do the same; n=1 wanted to sell a failing business to return to paid employment; n=1 wanted to work but their Visa status would not allow it; and n=1 answered that they were caring for a newborn.

For peer review only

Supplementary Table 1: Secondary outcome measures

Site	1	2	3	4	5
**Design & reporting framework**	Pilot RCT (as per published Protocol)[6] CONSORT			Pilot RCT (simplified processes) CONSORT	Pre-post evaluation STROBE
**State (metropolitan or regional)**	Victoria (metropolitan)	Victoria (regional)	New South Wales (metropolitan)	New South Wales (regional)	Victoria (metropolitan)
**Screening period**	3 March to 3 April 2020 (1 month)	26 August 2020 to 23 November 2021 (14 months)	2 September 2020 to 6 April 2021 (4 months)	18 Oct 2021 to 16 May 2022 (7 months)	21 June 2021 to 23 May 2022 (12 months)
**CFH service tier**	Universal	Higher intensity	Universal	Universal	Higher intensity
**Randomisation unit**	Individual			Individual	Not applicable
**Secondary outcomes [quantitative measures]: *Impact***	Caregiver-reported 3- and 6-months post-enrolment:<br>1. Household income, sources of income and types of loans [questions adapted from HILDA Survey[7], Longitudinal Study of Australian Children (LSAC)[8]].<br>2. Meeting household costs from HILDA,[7] Nest Survey,[9] Community Understanding Poverty & Social Exclusion (CUPSE) [10]].<br>3. Caregiver general health [General Health Questionnaire 1 (GHQ1)[11,12] and EuroQol five-dimensional questionnaire (EQ5D)[13,14]].<br>4. Caregiver mental health [Depression, Anxiety, Stress Scale short-form (DASS-21)[15]].<br>5. Parenting efficacy [questions from LSAC[8]].<br>6. Use of financial services [study-designed questions].			Caregiver-reported at 6-months post-enrolment:<br>1. Meeting household costs (from original RCT design).[7,9,10]<br>Emotional wellbeing:<br>2. 2-item Matthey Generic Mood Questionnaire (MGMQ),[16] dichotomised into emotional distress versus not.<br>3. 8-item Personal Wellbeing Index (PWI), scored on a 10-point scale ("no satisfaction at all" to "completely satisfied"), summed to generate a total score; higher scores indicate greater wellbeing.[17]	Financial-counsellor reported at case-close or 6 months post-referral (whichever was earlier):<br>1. 2-item Matthey Generic Mood Questionnaire (MGMQ)[16].<br>2. 8-item Personal Wellbeing Index (PWI)[17].

For peer review only - http://bmjopen.bmj.com/site/about/guidelines.xhtml

SUPPLEMENTARY REFRENCES

1. Phillips B, Gray M, Biddle N. COVID-19 JobKeeper and JobSeeker impacts on poverty and housing stress under current and alternative economic and policy scenarios. Acton, ACT: Australian National University Centre for Social Research and Methods; 2020.
2. Cassells R, Duncan A. JobKeepers and JobSeekers: How many workers will lose and how many will gain? Bentley, WA: Curtin University; 2020.
3. Gandy S. Care expands for moms, babies. Nursing & Allied Healthweek. 1996;1(13):1.
4. Klapdor M. Parliament of Australia. COVID-19 Economic response—free child care. https://www.aph.gov.au/About_Parliament/Parliamentary_Departments/Parliamentary_Library/FlagPost/2020/April/Coronavirus_response-Free_child_care [Accessed 26 March 2021]. 2020.
5. Davidson P. A tale of two pandemics: COVID, inequality and poverty in 2020 and 2021 ACOSS/UNSW Sydney Poverty and Inequality Partnership, Build Back Fairer Series, Report No. 3, Sydney. 2022.
6. Price AMH, Zhu A, Nguyen HNJ, Contreras-Suárez D, Schreurs N, Burley J, et al. Study protocol for the Healthier Wealthier Families (HWF) pilot randomised controlled trial: testing the feasibility of delivering financial counselling to families with young children who are identified as experiencing financial hardship by community-based nurses. BMJ Open. 2021;11(5):e044488.
7. Melbourne Institute: Applied Economics & Social Research. The Household, Income and Labour Dynamics In Australia Survey: Selected Findings from Waves 1 to 17. Report. Melbourne, Australia: The University of Melbourne, Melbourne Institute; 2019.
8. Australian Institute of Family Studies. (2018). Longitudinal Study of Australian Children Data User Guide – December 2018. Melbourne: Australian Institute of Family Studies. Accessed from https://growingupinaustralia.gov.au/data-and-documentation/data-user-guide.
9. Sollis K. Measuring child deprivation and opportunity in Australia: applying the Nest Framework to develop a measure of deprivation and opporutnity for children using the Longitudinal Study of Australian Children. Canberra; 2019 February.
10. Saunders P, Zhu A. Comparing Disadvantage and Well-being in Australian Families [online]. Australian Journal of Labour Economics. 2009;12(No. 1):21-39.
11. Raat H, Botterweck AM, Landgraf JM, Hoogeveen WC, Essink-Bot M-L. Reliability and validity of the short form of the child health questionnaire for parents (CHQ-PF28) in large random school based and general population samples. J Epidemiol Community Health. 2005;59(1):75-82.
12. Waters E, Salmon L, Wake M, Hesketh K, Wright M. The Child Health Questionnaire in Australia: reliability, validity and population means. Australian and New Zealand Journal of Public Health. 2000;24(2):207-10.
13. Group TE. EuroQol-a new facility for the measurement of health-related quality of life. Health policy. 1990;16(3):199-208.
14. Viney R, Norman R, King MT, Cronin P, Street DJ, Knox S, et al. Time trade-off derived EQ-5D weights for Australia. Value in Health. 2011;14(6):928-36.
15. Henry JD, Crawford JR. The short-form version of the Depression Anxiety Stress Scales (DASS-21): construct validity and normative data in a large non-clinical sample. British Journal of Clinical Psychology. 2005;44(2):227-39.
16. Price AMH, Middleton M, Matthey S, Goldfeld S, Kemp L, Orsini F. A comparison of two measures to screen for mental health symptoms in pregnancy and early postpartum: the Matthey Generic Mood Questionnaire and the Depression, Anxiety, Stress Scales short-form. Journal of Affective Disorders. 2021;281:824-33.
17. Cummins R. International Well Being Group: Personal Wellbeing Index. Melbourne: Australian Centre on Quality of Life, Deakin University. 2004.

For peer review only - http://bmjopen.bmj.com/site/about/guidelines.xhtml

Reporting checklist for randomised trial.

		Reporting Item	Page Number
**Title and Abstract**			
Title	#1a	Identification as a randomized trial in the title.	1[1]
Abstract	#1b	Structured summary of trial design, methods, results, and conclusions	2
**Introduction**			
Background and objectives	#2a	Scientific background and explanation of rationale	4
Background and objectives	#2b	Specific objectives or hypothesis	5
**Methods**			
Trial design	#3a	Description of trial design (such as parallel, factorial) including allocation ratio.	6, Table 1
Trial design	#3b	Important changes to methods after trial commencement (such as eligibility criteria), with reasons	7, Table 1
Participants	#4a	Eligibility criteria for participants	Table 1
Participants	#4b	Settings and locations where the data were collected	6
Interventions	#5	The experimental and control interventions for each group with sufficient details to allow replication, including how and when they were actually administered	Table 1
Outcomes	#6a	Completely defined prespecified primary and secondary outcome measures, including how and when they were assessed	Table 1
Outcomes	#6b	Any changes to trial outcomes after the trial commenced, with reasons	Table 1
Sample size	#7a	How sample size was determined.	Table 1
Sample size	#7b	When applicable, explanation of any interim analyses and stopping guidelines	n/a
Randomization - Sequence generation	#8a	Method used to generate the random allocation sequence.	Table 1
Randomization - Sequence generation	#8b	Type of randomization; details of any restriction (such as blocking and block size)	Table 1
Randomization - Allocation concealment mechanism	#9	Mechanism used to implement the random allocation sequence (such as sequentially numbered containers), describing any	Table 1

[1] NB, originally designed as an RCT, which was infeasible, the design changed throughout the study and the title reflects this with 'mixed methods'.

For peer review only

		Reporting Item	Page Number
		steps taken to conceal the sequence until interventions were assigned	
Randomization - Implementation	#10	Who generated the allocation sequence, who enrolled participants, and who assigned participants to interventions	Table 1
Blinding	#11a	If done, who was blinded after assignment to interventions (for example, participants, care providers, those assessing outcomes) and how.	Table 1
Blinding	#11b	If relevant, description of the similarity of interventions	Table 1
Statistical methods	#12a	Statistical methods used to compare groups for primary and secondary outcomes	Table 1
Statistical methods	#12b	Methods for additional analyses, such as subgroup analyses and adjusted analyses	n/a
**Results**			
Participant flow diagram (strongly recommended)	#13a	For each group, the numbers of participants who were randomly assigned, received intended treatment, and were analysed for the primary outcome	Supp Figure 1
Participant flow	#13b	For each group, losses and exclusions after randomization, together with reason	8, Table 3
Recruitment	#14a	Dates defining the periods of recruitment and follow-up	Table 1, Figure 1
Recruitment	#14b	Why the trial ended or was stopped	6 (Site 1)
Baseline data	#15	A table showing baseline demographic and clinical characteristics for each group	Supp Table 2
Numbers analysed	#16	For each group, number of participants (denominator) included in each analysis and whether the analysis was by original assigned groups	8, Supp Figure 1
Outcomes and estimation	#17a	For each primary and secondary outcome, results for each group, and the estimated effect size and its precision (such as 95% confidence interval)	n/a
Outcomes and estimation	#17b	For binary outcomes, presentation of both absolute and relative effect sizes is recommended	n/a
Ancillary analyses	#18	Results of any other analyses performed, including subgroup analyses and adjusted analyses, distinguishing pre-specified from exploratory	n/a
Harms	#19	All important harms or unintended effects in each group (For specific guidance see CONSORT for harms)	n/a
**Discussion**			
Limitations	#20	Trial limitations, addressing sources of potential bias, imprecision, and, if relevant, multiplicity of analyses	14

For peer review only

		Reporting Item	Page Number
Generalisability	#21	Generalisability (external validity, applicability) of the trial findings	14
Interpretation	#22	Interpretation consistent with results, balancing benefits and harms, and considering other relevant evidence	13-14
Registration	#23	Registration number and name of trial registry	2
**Other information**			
Interpretation	#22	Interpretation consistent with results, balancing benefits and harms, and considering other relevant evidence	13-15
Registration	#23	Registration number and name of trial registry	2
Protocol	#24	Where the full trial protocol can be accessed, if available	3
Funding	#25	Sources of funding and other support (such as supply of drugs), role of funders	3

None The CONSORT checklist is distributed under the terms of the Creative Commons Attribution License CC-BY. This checklist can be completed online using https://www.goodreports.org/, a tool made by the EQUATOR Network in collaboration with Penelope.ai

For peer review only
